# Supplementary material for: Unequal gains from remote work during COVID-19 between spouses: Evidence from longitudinal data in Singapore
Source: PLoS One. 2025 May 20;20(5):e0324113. doi: 10.1371/journal.pone.0324113 (PMC12091887; doi:10.1371/journal.pone.0324113)
Supplement: S1 Text — (DOCX) [file pone.0324113.s002.docx]

**S1 Text: Labor Market in Singapore**

According to *2020 Labour Force in Singapore,*^1^ out of the total population of 1,152,800 of male Singaporean residents aged 25-64 in 2020, 92.0% were in the labor force. Among the total of 1,218,800 female Singaporean residents within the same age range, 76.6% were in the labor force. Leaving aside the unemployed persons (i.e., active job-seekers), employment rate for Singaporean residents aged 25-64 in 2020 was 87.9% out of a total for men and 73.2% for women. Among the employed male residents, approximately 92.5% worked full-time and 7.5% worked part time. For female residents, about 85.8% worked full-time and 14.2% in part-time positions. Overall, the Singapore’s labor market in 2020 portrayed clear gender differences—with men exhibiting greater labor force participations, larger employment rates, and better job security proxied by their attachment to full-time positions. As for the occupation-specific distributions, we observe that out of the total of 1,198,100 employed male Singaporeans *across all ages* in 2020, 60.4% were in high-income, white-collar occupations (managers/ administrators/ professionals/ assoc. professionals/ technicians); 13.3% in the remaining white-collar occupations (clerical support, service & sales); 4.7% in skilled manual positions (craftsman and trade workers); and 21.8% in semi-skilled or low-skilled manual jobs (plant and machine operators/assemblers/cleaners/laborers/other related workers). Among the total of 1,024,600 employed females *across all ages* in the same year, 59.3% were in high-income, white-collar occupations; 30.1% in the remaining white-collar occupations; 0.8% in skilled manual positions; and 9.8% in semi-skilled or low-skilled manual jobs. While the heavy feminization of the clerical and services & sales positions was consistent with the occupational distribution in other countries, Singapore’s workforce tended to be predominantly white-collar—with high shares for both genders compared to their counterparts in other developed nations. Of note, statistics from the Ministry of Manpower does not divide the occupation-specific distributions by age groups.^1^ Thus, the total here differs from the one reported in the previous paragraph that falls within ages 25-64. Regarding the industry-specific distributions, out of the total of 1,198,100 males across all ages in 2020, 11.3% worked in the manufacturing industry; 5.8%, construction; 81.7%, services; and 1.3%, others. Among the total of 1,024,600 females, 7.6% operated in manufacturing sector; 2.8%, construction; 89.1%, services; and 0.6%, others. While Singapore had more male workers in the manufacturing and construction sectors, the services sector encompassed more females. Overall, Singaporean residents primarily operated in the services sector—regardless of gender. The evolution of the occupation- and industry- distributions across gender between 2010-2020 are displayed in **S1** **Table** and **S2** **Table**.

**References**

1. Ministry of Manpower. Report: Labour Force In Singapore 2020 [Internet]. 2021 [cited 2024 Jan 17]. Available from: https://stats.mom.gov.sg/Pages/Labour-Force-In-Singapore-2021.aspx
