# Supplementary material for: Unequal gains from remote work during COVID-19 between spouses: Evidence from longitudinal data in Singapore
Source: PLoS One. 2025 May 20;20(5):e0324113. doi: 10.1371/journal.pone.0324113 (PMC12091887; doi:10.1371/journal.pone.0324113)
Supplement: S2 Text — (DOCX) [file pone.0324113.s003.docx]

**S2 Text: Sensitivity Analyses - Using a Broader Definition of the Remote Work Indicator**

Our data contained information on individuals’ remote work statuses—whether working fully from home, mostly from home, half from home and half outside the home, mostly outside the home or fully outside the home. From this, we created a binary indicator of individuals fully worked from home—to be used as the main explanatory variable. We intentionally used a rigid definition of remote work in order to distinguish the labor market outcomes of individuals who fully transitioned into working from home from that of the rest (no or partial WFH)—who would have been subject to more work-related disruptions or health-risks following the COVID-19 outbreak.

In this exercise, we have taken on a broader definition of remote work, setting the remote work variable to indicate both full WFH and partial WFH individuals. **S8** **Table** displays results from the main difference-in-differences estimation—same as the one shown in **Table 4** in the manuscript. Comparing the results to that of Table 4, we see several points of divergence.

In columns (4)-(6) of both **Table 4** and **S8** **Table**, WFH (fully or partially) during the Lockdown leads to an increased monthly income—but only for men and not for women. Yet, while the income gain is still significant in the post-lockdown phase for the full WFH workers (in **Table 4** in the manuscript), the gain dissipates when we include partial WFH workers in the table below. The income gain for remote workers in both tables comes from increased hourly wages, not due to increased work hours. Yet, while the productivity gain seen during the Lockdown heightens in the post-Lockdown phase for the full WFH workers (columns 1-3 of **Table 4**), it reduces—although still statistically significant—when we include partial WFH in the definition (columns 1-3 in **S8** **Table**). Of note, the wage gain is not a result of the increased demands for remotely-conducted occupations: We have controlled for any changes in the occupation-specific demands by including occupation-specific time trends in all estimations. The change in the work environment and productivity seems to be what is driving the change in hourly wage. Despite that WFH is generally associated with a reduction of work hours in both tables, such a reduction applies only to female full WFH workers (columns 7-9 in **Table 4**). When we include partial WFH, the reduction in the work hours is experienced by both genders.

Overall, we observe that the partial WFH workers’ labor market outcomes are somewhat different from that of the full WFH—with smaller gains in income and a greater reduction in work hours for partial WFH. Including partial WFH workers in our binary explanatory variable on the remote work arrangements would diffuse the effect of full WFH.
