# Supplementary material for: Unequal gains from remote work during COVID-19 between spouses: Evidence from longitudinal data in Singapore
Source: PLoS One. 2025 May 20;20(5):e0324113. doi: 10.1371/journal.pone.0324113 (PMC12091887; doi:10.1371/journal.pone.0324113)
Supplement: S3 Text — (DOCX) [file pone.0324113.s004.docx]

**S3 Text: Exploring the Links between WFH and Childcare/Chores**

To empirically test our concerns regarding the link (or the substitutability) between WFH and household responsibilities, per your advice, we inspect the effects of WFH on time spent on **childcare** at time t, using the same covariate and fixed-effects specifications as in equation (1) in the manuscript. The results are displayed in **S9-1** **Table**. From columns (1)-(3), we observe that the WFH is positively associated with the childcare time. The more of the work is done remotely from home, the more childcare time one spends—and this is especially true for women. During the lockdown and in the post-lockdown period, shown in columns (4)-(6), we observe that the WHF is positively associated for men who adopted full-remote work arrangements, while is negatively associated with women who partially or fully adopted WFH. One possible interpretation may be that the WFH and childcare are substitutes for mothers, while the two activities are complements for fathers. The blurred boundary between home and work-space—facilitated by the adoption of WFH—took a toll on childcare time for mothers. For fathers, working from home incentivized them to do take on more childcare duties. Regardless of the interpretations, the divergent relationship between WFH and childcare load across gender is noteworthy and is suggestive of the non-mechanical linkage between the two activities (i.e., an increase in one does not automatically lead to a decrease in the other). A traditional view that WFH and household production are substitutes would be validated if the estimated coefficients of the WFH in **S9-1** **Table** are negative.

Next, we estimated the effect of WFH on time spent on **chores** at time t, as shown in **S9-2 Table** below. While the WFH is once again positively associated with chore time in columns (1)-(3), the link between WFH and chore time is noisier. Specifically, for mothers (column 3), both respondents who work mostly outside or mostly from home (i.e., those conducting work partially from home, albeit at varying rates) incur greater chore times compared to those who work fully from home. Yet, this link holds true only for mothers and not fathers. There seems to be no systematic association between the fathers’ remote work adoption and chore time. During the lockdown period as shown in columns (4)-(6)), we observe that WFH is strictly and positively related to chore time. This pattern applies for both fathers and mothers—suggesting that the extent of their engagements with household responsibilities increased during the lockdown compared to the previous years. In the post-lockdown period, the systematic link between WFH and chore time subsides for both genders. Same as before, the results on Table A9-2 seem to indicate that WFH and chores are not perfect substitutes—thereby weakening the traditionally-believed mechanical link between the two activities.
