# Supplementary material for: Unequal gains from remote work during COVID-19 between spouses: Evidence from longitudinal data in Singapore
Source: PLoS One. 2025 May 20;20(5):e0324113. doi: 10.1371/journal.pone.0324113 (PMC12091887; doi:10.1371/journal.pone.0324113)
Supplement: S1 Table — (DOCX) [file pone.0324113.s005.docx]

| **S1 Table. Employed Singaporean Residents Aged 15+ by Occupation and Gender, 2010-2020 (in 000)** | | | | | | | | |  |  |  |
| --- | --- | --- | --- | --- | --- | --- | --- | --- | --- | --- | --- |
| *PANEL 1: MALES* |  |  |  |  |  |  |  |  |  |  |  |
| Occupation | 2010 | 2011 | 2012 | 2013 | 2014 | 2015 | 2016 | 2017 | 2018 | 2019 | 2020 |
| Managers & Administrators | 232.7 | 243.5 | 238.8 | 239.8 | 233.3 | 235.2 | 220.2 | 230.6 | 219.2 | 231.0 | 231.4 |
| Professionals | 207.4 | 183.2 | 198.9 | 195.5 | 205.3 | 209.3 | 215.1 | 229.0 | 236.0 | 238.0 | 260.5 |
| Assoc. Professionals & Technicians | 167.1 | 182.6 | 184.6 | 195.1 | 202.7 | 214.6 | 229.9 | 224.8 | 242.5 | 243.2 | 231.7 |
| Clerical Support Workers | 52.9 | 57.2 | 61.4 | 65.1 | 66.4 | 63.3 | 60.0 | 58.4 | 59.9 | 56.3 | 51.7 |
| Service & Sales Workers | 108.9 | 107.4 | 115.7 | 112.1 | 110.7 | 113.0 | 115.6 | 108.3 | 115.5 | 111.7 | 107.6 |
| Craftsmen & Related Trades Workers | 78.0 | 82.1 | 81.7 | 74.9 | 75.2 | 74.2 | 69.8 | 67.1 | 60.9 | 58.8 | 53.7 |
| Plant & Machine Operators & Assemblers | 126.3 | 131.7 | 124.8 | 125.0 | 126.4 | 128.7 | 132.6 | 136.0 | 130.4 | 136.8 | 137.2 |
| Cleaners, Laborers & Related Workers | 66.6 | 62.9 | 64.7 | 67.3 | 72.1 | 65.6 | 65.6 | 68.4 | 66.2 | 64.4 | 65.3 |
| Others | 66.7 | 68.2 | 67.7 | 67.6 | 68.9 | 67.9 | 69.7 | 67.0 | 66.6 | 62.4 | 59.0 |
| Total | 1,106.60 | 1,118.80 | 1,138.10 | 1,142.30 | 1,161.00 | 1,171.80 | 1,178.40 | 1,189.40 | 1,197.20 | 1,202.50 | 1,198.10 |
| *PANEL 2: FEMALES* |  |  |  |  |  |  |  |  |  |  |  |
| Occupation | 2010 | 2011 | 2012 | 2013 | 2014 | 2015 | 2016 | 2017 | 2018 | 2019 | 2020 |
| Managers & Administrators | 119.7 | 123.5 | 120.2 | 120.7 | 118.0 | 120.6 | 117.3 | 118.3 | 122.4 | 133.9 | 136.9 |
| Professionals | 168.3 | 165.2 | 171.1 | 176.4 | 178.0 | 194.8 | 204.7 | 208.6 | 213.3 | 231.4 | 243.7 |
| Assoc. Professionals & Technicians | 136.3 | 154.5 | 155.8 | 164.3 | 188.9 | 193.7 | 208.2 | 211.4 | 222.5 | 224.7 | 226.5 |
| Clerical Support Workers | 190.7 | 192.1 | 204.3 | 206.6 | 202.0 | 204.4 | 184.9 | 189.5 | 181.1 | 180.9 | 163.9 |
| Service & Sales Workers | 125.6 | 127.4 | 136.8 | 132.6 | 133.6 | 138.5 | 146.8 | 139.7 | 147.2 | 146.0 | 144.7 |
| Craftsmen & Related Trades Workers | 8.5 | 7.4 | 8.8 | 7.3 | 6.9 | 9.7 | 9.4 | 8.9 | 9.1 | 8.9 | 8.7 |
| Plant & Machine Operators & Assemblers | 26.3 | 25.9 | 23.9 | 21.9 | 19.5 | 17.4 | 18.6 | 15.5 | 17.5 | 14.6 | 14.5 |
| Cleaners, Laborers & Related Workers | 78.9 | 82.8 | 80.6 | 82.9 | 93.8 | 94.7 | 95.1 | 92.1 | 90.2 | 85.1 | 84.2 |
| Others | 2.0 | 1.4 | 0.9 | 1.1 | 1.9 | 2.1 | 1.9 | 1.9 | 3.2 | 2.4 | 1.4 |
| Total | 856.4 | 880.1 | 902.5 | 913.8 | 942.5 | 976 | 986.9 | 985.9 | 1,006.50 | 1,027.90 | 1,024.60 |
| Source: Ministry of Manpower (2021). 2020 Labour Force in Singapore, Table 35. | | | | | |  |  |  |  |  |  |
