# Supplementary material for: Unequal gains from remote work during COVID-19 between spouses: Evidence from longitudinal data in Singapore
Source: PLoS One. 2025 May 20;20(5):e0324113. doi: 10.1371/journal.pone.0324113 (PMC12091887; doi:10.1371/journal.pone.0324113)
Supplement: S2 Table — (DOCX) [file pone.0324113.s006.docx]

| **S2 Table. Employed Singaporean Residents Aged 15+ by Industry and Gender, 2020 (in 000)** | | |
| --- | --- | --- |
| Industry | Males | Females |
| Manufacturing | 213.0 | 135.1 |
| Construction | 97.2 | 68.9 |
| Services | 1891.3 | 978.6 |
| Wholesale & Retail Trade | 335.1 | 167.6 |
| Transportation & Storage | 214.8 | 162.2 |
| Accommodation & Food Services | 128.9 | 55.9 |
| Information & Communication | 111.4 | 66.4 |
| Financial & Insurance Services | 207.9 | 100.1 |
| Real Estate Services | 49.8 | 23.9 |
| Professional Services | 181.4 | 96.6 |
| Administrative & Support Services | 128.3 | 75.3 |
| Public Administration & Education | 293.9 | 155.9 |
| Health & Social Services | 139.9 | 31.8 |
| Arts, Entertainment & Recreation | 34.4 | 17.1 |
| Other Community, Social & Personal Services | 65.4 | 25.8 |
| Others | 21.2 | 15.5 |
| Total | 2222.6 | 1198.1 |
| Source: Ministry of Manpower (2021). 2020 Labour Force in Singapore, Table 36. | | |
