# Supplementary material for: Unequal gains from remote work during COVID-19 between spouses: Evidence from longitudinal data in Singapore
Source: PLoS One. 2025 May 20;20(5):e0324113. doi: 10.1371/journal.pone.0324113 (PMC12091887; doi:10.1371/journal.pone.0324113)
Supplement: S3 Table — (DOCX) [file pone.0324113.s007.docx]

**S3 Table. Occupational Categories in the Survey**

| **Professionals** | |  | **Clerical Support Workers** |  |
| --- | --- | --- | --- | --- |
| 1 | Legislator, Senior Official and Chief Executives | 19 | Clerical Supervisor |  |
| 2 | Administrative and Commercial Manager | 20 | General and Keyboard Clerk |  |
| 3 | Production and Specialised Services Manager | 21 | Customer Services Officers and Clerk |  |
| 4 | Hospitality, Retail and related Services Manager | 22 | Numerical and Material-Recording Clerk |  |
| 5 | Science and Engineering Professional | 23 | Others ***PLEASE SPECIFY*** |  |
| 6 | Health Professional | **Service and Sales Workers** | |  |
| 7 | Teaching and Training Professional | 24 | Personal Service Worker | |
| 8 | Business and Administration Professional | 25 | Sales Worker | |
| 9 | Information and Communications Technology Professional | 26 | Personal Care Worker | |
| 10 | Legal, Social and Cultural Professionals | 27 | Protective Services Worker | |
| 11 | Others ***PLEASE SPECIFY*** | 28 | Customer Service Worker | |
| **Associate Professionals And Technicians** | | 29 | Others ***PLEASE SPECIFY*** | |
| 12 | Physical and Engineering Science associate professionals | 30 Occupation unknown/ Other | |  |
| 13 | Health Associate Professional |  |  |  |
| 14 | Business and Administration Associate Professional |  |  |  |
| 15 | Legal, Social, Cultural and related Associate Professional |  |  |  |
| 16 | Information and Communications Technician |  |  |  |
| 17 | Teaching Associate Professional |  |  |  |
| 18 | Others ***PLEASE SPECIFY*** |  |  |  |
