# Supplementary material for: Unequal gains from remote work during COVID-19 between spouses: Evidence from longitudinal data in Singapore
Source: PLoS One. 2025 May 20;20(5):e0324113. doi: 10.1371/journal.pone.0324113 (PMC12091887; doi:10.1371/journal.pone.0324113)
Supplement: S4 Table — (DOCX) [file pone.0324113.s008.docx]

**S4 Table. Remote Work Preferences Among Female Respondents in November 2020**

| Preferences | Count | % |
| --- | --- | --- |
| Strongly favoring | 107 | 37.54 |
| Somewhat favoring | 103 | 36.14 |
| No preference | 36 | 12.63 |
| Somewhat not favoring | 26 | 9.12 |
| Strongly not favoring | 13 | 4.56 |
| N | 285 |  |

Notes: Respondents are asked: “Given a choice, would you be in favor of a permanent work-from-home arrangement?”
