# Supplementary material for: Unequal gains from remote work during COVID-19 between spouses: Evidence from longitudinal data in Singapore
Source: PLoS One. 2025 May 20;20(5):e0324113. doi: 10.1371/journal.pone.0324113 (PMC12091887; doi:10.1371/journal.pone.0324113)
Supplement: S5 Table — (DOCX) [file pone.0324113.s009.docx]

**S5 Table. Comparison of Baseline Characteristics in the Sample and National Statistics of Married Resident Mothers Aged 25-59**

|  | Sample (%) | National (%) |
| --- | --- | --- |
| Chinese | 93.19 | 74.63 |
| Number of children |  |  |
| 0 | 14.36 | 17.24 |
| 1 | 32.83 | 24.25 |
| 2 | 43.01 | 37.92 |
| 3 | 7.40 | 15.31 |
| 4 | 2.09 | 3.97 |
| 5 | 0.00 | 1.31 |
| Occupation (excluding non-employed)^+^ |  |  |
| Non-professionals | 21.24 | 40.75 |
| Professionals | 78.76 | 59.25 |
| Have a college degree^++^ | 71.58 | 58.19 |
| Monthly income (excluding non-employed)^+^ |  |  |
| Less than $2,000 | 8.97 | 18.46 |
| $2,000-$4,000 | 42.71 | 34.21 |
| $4,000-$6,000 | 34.03 | 21.48 |
| $6,000-$8,000 | 20.34 | 9.84 |
| Greater than $8000 | 4.71 | 16.01 |
| Total | 1,337 | 734,083 |

^+^ National statistic based on women of all ages and marital status.

^++^ National statistic based on married women of all ages

Note: National statistics for age, race, number of children and education are taken from the Census of Population 2020. National statistics on Occupation and Monthly income were taken from the 2020 Labour Force in Singapore by the Ministry of Manpower (2021). Statistics for occupation and income are recalculated to include only employed female workers. The “professionals” category in occupation includes professionals, associate professionals, and technicians.
