# Supplementary material for: Unequal gains from remote work during COVID-19 between spouses: Evidence from longitudinal data in Singapore
Source: PLoS One. 2025 May 20;20(5):e0324113. doi: 10.1371/journal.pone.0324113 (PMC12091887; doi:10.1371/journal.pone.0324113)
Supplement: S6 Table — (DOCX) [file pone.0324113.s010.docx]

**S6 Table. Average Working Hours by Month and Remote Work Arrangements**

|  | July 2018 | | May 2020 | | Nov 2020 | |  |
| --- | --- | --- | --- | --- | --- | --- | --- |
|  | Male | Female | Male | Female | Male | Female | |
| All | 46.86 | 42.59 | 41.05 | 37.37 | 45.80 | 41.31 | |
| Fully outside | 50.00 | 42.77 | 47.14 | 40.43 | 49.85 | 41.96 | |
| Mostly outside | 47.34 | 42.22 | 43.38 | 46.00 | 47.68 | 41.82 | |
| Half from home and half outside | 46.98 | 41.70 | 41.36 | 37.35 | 43.92 | 46.43 | |
| Mostly from home | 44.92 | 43.41 | 37.45 | 38.46 | 40.78 | 40.27 | |
| Fully from home | 45.48 | 42.61 | 37.96 | 35.68 | 40.32 | 34.97 | |
| N | 384 | 335 | 384 | 335 | 381 | 332 | |
