# Supplementary material for: Unequal gains from remote work during COVID-19 between spouses: Evidence from longitudinal data in Singapore
Source: PLoS One. 2025 May 20;20(5):e0324113. doi: 10.1371/journal.pone.0324113 (PMC12091887; doi:10.1371/journal.pone.0324113)
Supplement: S7 Table — (DOCX) [file pone.0324113.s011.docx]

| **S7 Table.** **Assessment of Pre-Trends: Monthly Income and Monthly Hours Worked** | | | | | | |
| --- | --- | --- | --- | --- | --- | --- |
|  | (1) | (2) | (3) | (4) | (5) | (6) |
|  | Y= monthly income | | | Y= monthly hours worked | | |
|  | All | Male | Female | All | Male | Female |
|  | |  |  |  |  |  |
| **TREATED x TIME PERIODS** | |  |  |  |  |  |
| Working Remotely x  Apr-Jul 2018 | -123.27 | -65.01 | -145.05 | -0.01 | 0.01 | -0.01 |
|  | (84.05) | (139.69) | (96.16) | (0.00) | (0.00) | (0.00) |
| Working Remotely x  March 2020 | 254.48** | 464.77*** | 61.84 | -0.01 | 0.01 | -0.01 |
|  | (102.08) | (160.77) | (113.37) | (0.00) | (0.00) | (0.00) |
| Working Remotely x  May 2020 | 252.51** | 455.35** | 45.01 | -12.27* | -10.96 | -15.55* |
|  | (117.90) | (184.53) | (135.75) | (6.82) | (8.87) | (9.17) |
| Working Remotely x  June 2020 | 194.95 | 400.39* | -17.13 | -21.06*** | -15.31* | -30.77*** |
|  | (140.08) | (216.86) | (163.04) | (6.38) | (8.26) | (8.81) |
| Working Remotely x  Nov 2020 | 233.44 | 477.46** | 59.78 | -37.29*** | -21.88* | -52.60*** |
|  | (154.67) | (224.86) | (195.22) | (9.88) | (11.17) | (15.55) |
| **TIME PERIODS** |  |  |  |  |  |  |
| Apr-Jul 2018 | -10.36 | 16.74 | -54.42 | 0.01 | 0.01 | 0.01 |
|  | (58.69) | (80.01) | (74.07) | (0.00) | (0.00) | (0.00) |
| Mar-20 | -146.37*** | -182.01*** | -88.44 | 0.01 | -0.01 | 0.01 |
|  | (50.39) | (69.05) | (59.50) | (0.00) | (0.00) | (0.00) |
| May-20 | -205.96*** | -265.69*** | -108.84 | -16.47*** | -19.12*** | -12.16* |
|  | (63.06) | (78.05) | (84.30) | (4.77) | (5.78) | (6.45) |
| Jun-20 | -182.67*** | -256.33*** | -70.95 | -4.85 | -10.87** | 4.98 |
|  | (59.73) | (79.12) | (77.10) | (4.63) | (5.30) | (6.51) |
| Nov-20 | 78.07 | 76.79 | 89.38 | 3.42 | -2.48 | 12.95* |
|  | (64.94) | (85.14) | (83.98) | (5.01) | (5.67) | (7.43) |
| **TREATED** |  |  |  |  |  |  |
| Working Remotely  (1 yes 0 no) | -254.96** | -491.57*** | -38.18 | 8.04 | -2.07 | 21.18** |
|  | (113.99) | (189.19) | (126.94) | (6.13) | (7.48) | (9.37) |
|  |  |  |  |  |  |  |
| Individual FE | Yes | Yes | Yes | Yes | Yes | Yes |
| N | 4308 | 2301 | 2007 | 4308 | 2301 | 2007 |
| Notes: Panel A and Panel B are estimated separately. Remote is a dichotomous variable for whether the respondent worked fully from home in May 2020 during the COVID-19 lockdown. The reference time period is Dec 2019, prior to the COVID-19 pandemic. Standard errors, shown in the parentheses, are clustered at the household level. | | | | | | |
