# Supplementary material for: Unequal gains from remote work during COVID-19 between spouses: Evidence from longitudinal data in Singapore
Source: PLoS One. 2025 May 20;20(5):e0324113. doi: 10.1371/journal.pone.0324113 (PMC12091887; doi:10.1371/journal.pone.0324113)
Supplement: S8 Table — (DOCX) [file pone.0324113.s012.docx]

| **S8 Table.** **Effects of Remote Work Arrangements on Hourly Wage, Monthly Income, and Monthly Work Hours** | | | | | | | | | | |
| --- | --- | --- | --- | --- | --- | --- | --- | --- | --- | --- |
|  | (1) | (2) | (3) | (4) | (5) | (6) | (7) | (8) | (9) |  |
|  | Y=Hourly Wage | | | Y=Monthly Income | | | Y=Monthly Hours Worked | | | |
|  | All | Male | Female | All | Male | Female | All | Male | Female |  |
| (reference = Pre-Lockdown) | | |  |  |  |  |  |  |  |  |
| **Working Remotely x Lockdown** | 10.58*** | 7.32* | 14.33*** | 277.11*** | 276.40* | 172.54 | -9.56** | -9.18* | -12.46** |  |
|  | (2.99) | (3.81) | (4.30) | (102.09) | (154.73) | (112.08) | (3.79) | (5.07) | (5.57) |  |
| **Working Remotely x Post- lockdown** | 9.62*** | 9.96* | 11.28*** | 166.14 | 172.51 | 88.48 | -23.37*** | -17.54* | -31.88*** |  |
|  | (3.58) | (5.67) | (4.18) | (125.45) | (197.76) | (146.67) | (6.85) | (9.87) | (10.55) |  |
|  |  |  |  |  |  |  |  |  |  |  |
| Working Remotely  (1 yes 0 no) | 2.74 | 3.80 | 0.25 | -262.13** | -264.74 | -157.81 | -3.55 | -3.06 | 0.19 |  |
|  | (3.36) | (4.62) | (4.41) | (109.82) | (176.11) | (114.74) | (6.08) | (8.69) | (9.51) |  |
|  |  |  |  |  |  |  |  |  |  |  |
| Lockdown | 6.80** | 9.76** | 3.68 | -160.93* | -174.33 | -99.12 | -3.74 | -10.12* | 3.03 |  |
|  | (2.65) | (3.96) | (2.60) | (84.83) | (119.75) | (103.83) | (4.18) | (5.43) | (5.65) |  |
| Post-lockdown | 7.71** | 10.22** | 4.20 | 127.67 | 190.33 | 83.95 | 4.99 | -1.18 | 12.34 |  |
|  | (3.25) | (4.45) | (3.61) | (95.03) | (135.55) | (116.78) | (6.15) | (7.93) | (7.59) |  |
|  |  |  |  |  |  |  |  |  |  |  |
| Individual FE & Occu. FE | Yes | Yes | Yes | Yes | Yes | Yes | Yes | Yes | Yes |  |
| Occu. time trends | Yes | Yes | Yes | Yes | Yes | Yes | Yes | Yes | Yes |  |
| Control variables | Yes | Yes | Yes | Yes | Yes | Yes | Yes | Yes | Yes |  |
| N | 4308 | 2301 | 2007 | 4308 | 2301 | 2007 | 4308 | 2301 | 2007 |  |
| Notes: Remote is a binary variable for whether the respondent worked fully or partially from home in May 2020 during the COVID-19 lockdown. The reference time period is ‘Pre-lockdown’, between April-July 2018 and December 2019, prior to the COVID-19 pandemic. ‘Lockdown’ refers to March and June 2020, while ‘Post-lockdown’ refers to November 2020, six months after the end of the lockdown. Standard errors, shown in the parentheses, are clustered at the household level. | | | | | | | | | | |
| *p<0.1 **p<0.05 ***p<0.01 | | | | | | | | | | |
