# Supplementary material for: Unequal gains from remote work during COVID-19 between spouses: Evidence from longitudinal data in Singapore
Source: PLoS One. 2025 May 20;20(5):e0324113. doi: 10.1371/journal.pone.0324113 (PMC12091887; doi:10.1371/journal.pone.0324113)
Supplement: S9 Table — (DOCX) [file pone.0324113.s013.docx]

| **S9 Table. Effect of Remote Work Arrangements (Categorical) on Time Spent on Childcare** | | | | | | | | |
| --- | --- | --- | --- | --- | --- | --- | --- | --- |
|  | (1) | (2) | (3) | (4) | (5) | (6) | |  |
|  | Y=Childcare (min/hr) | | | | | | | |
|  | All | Male | Female | All | Male | Female | |  |
| *Work Arrangements(Reference = Work Fully Outside)* | | | |  |  |  | |  |
| Mostly outside | 0.64 | 0.23 | 1.70** | 0.69 | 0.83 | 3.58* | |  |
|  | (0.50) | (0.66) | (0.83) | (0.92) | (1.02) | (1.89) | |  |
| Half from home | 1.22** | 0.47 | 2.29** | 1.59* | 1.08 | 3.54** | |  |
|  | (0.60) | (0.63) | (1.01) | (0.96) | (1.09) | (1.53) | |  |
| Mostly from home | 2.93*** | 1.26* | 4.57*** | 2.60** | -0.43 | 6.62** | |  |
|  | (0.63) | (0.67) | (1.01) | (1.20) | (1.36) | (3.35) | |  |
| Working remotely (Fully from home) | 2.64*** | 1.45** | 4.03*** | 1.29 | 0.57 | 3.59*** | |  |
|  | (0.59) | (0.59) | (0.97) | (0.90) | (0.88) | (1.37) | |  |
| *Lockdown Policy (Reference = Pre-Lockdown)* | | | |  |  |  | |  |
| Lockdown | - | - | - | -0.33 | 0.08 | 0.04 | |  |
|  | - | - | - | (0.65) | (0.62) | (1.15) | |  |
| Post-lockdown | - | - | - | -0.16 | 0.48 | -0.20 | |  |
|  | - | - | - | (0.79) | (0.96) | (1.11) | |  |
| (*Reference = Pre-Lockdown*, *Working Fully Outside)* | | | |  |  |  | |  |
| **Mostly outside x Lockdown** | - | - | - | 0.27 | -0.64 | -1.54 | |  |
|  | - | - | - | (0.90) | (0.89) | (1.99) | |  |
| **Half from home x Lockdown** | - | - | - | 0.07 | -0.75 | -0.73 | |  |
|  | - | - | - | (0.96) | (0.98) | (1.44) | |  |
| **Mostly from home x Lockdown** | - | - | - | 0.93 | 2.09 | -1.55 | |  |
|  | - | - | - | (1.19) | (1.44) | (3.38) | |  |
| **Working remotely (Fully from home) x Lockdown** | - | - | - | 1.69** | 1.37* | -0.15 | |  |
|  | - | - | - | (0.81) | (0.79) | (1.23) | |  |
|  |  |  |  |  |  |  | |  |
| **Mostly outside x Post-lockdown** | - | - | - | -0.27 | -0.78 | -2.93 | |  |
|  | - | - | - | (1.26) | (1.30) | (2.56) | |  |
| **Half from home x Post-lockdown** | - | - | - | -1.04 | -0.83 | -2.63* | |  |
|  | - | - | - | (0.99) | (1.18) | (1.49) | |  |
| **Mostly from home x Post-lockdown** | - | - | - | -0.89 | 2.03 | -4.92 | |  |
|  | - | - | - | (1.33) | (1.66) | (4.12) | |  |
| **Working remotely (Fully from home) x Post-lockdown** | - | - | - | 0.05 | 2.14* | -2.97* | |  |
|  | - | - | - | (1.26) | (1.23) | (1.73) | |  |
|  |  |  |  |  |  |  | |  |
| Individual FE, Occupation FE, Time FE | Yes | Yes | Yes | Yes | Yes | Yes | |  |
| Occupation FE x Time FE | Yes | Yes | Yes | Yes | Yes | Yes | |  |
| Control variables | Yes | Yes | Yes | Yes | Yes | Yes | |  |
| N | 4308 | 2301 | 2007 | 4308 | 2301 | 2007 | |  |
| *p<0.1 **p<0.05 ***p<0.01 | | | | | | |  | |
