# Supplementary material for: Unequal gains from remote work during COVID-19 between spouses: Evidence from longitudinal data in Singapore
Source: PLoS One. 2025 May 20;20(5):e0324113. doi: 10.1371/journal.pone.0324113 (PMC12091887; doi:10.1371/journal.pone.0324113)
Supplement: S10 Table — (DOCX) [file pone.0324113.s014.docx]

| **S10 Table. Effect of Remote Work Arrangements (Categorical) on Time Spent on Chores** | | | | | | |
| --- | --- | --- | --- | --- | --- | --- |
|  | (1) | (2) | (3) | (4) | (5) | (6) |
|  | Y=Chores (min/hr) | | | | | |
|  | All | Male | Female | All | Male | Female |
| *Work Arrangements(Reference = Work Fully Outside)* | | | | | | |
| Mostly outside | 0.34 | -0.26 | 1.07* | 0.21 | -0.41 | 0.73 |
|  | (0.34) | (0.34) | (0.65) | (0.72) | (0.68) | (1.56) |
| Half from home | -0.02 | -0.32 | 0.26 | -0.72 | -0.88 | -0.32 |
|  | (0.30) | (0.37) | (0.44) | (0.46) | (0.55) | (0.71) |
| Mostly from home | 0.68* | 0.13 | 1.34** | -0.23 | -0.57 | 0.45 |
|  | (0.38) | (0.38) | (0.63) | (0.49) | (0.64) | (0.85) |
| Working remotely (Fully from home) | 0.26 | -0.15 | 0.81 | -0.70* | -0.68 | -0.17 |
|  | (0.31) | (0.36) | (0.51) | (0.39) | (0.44) | (0.67) |
| *Lockdown Policy (Reference = Pre-Lockdown)* |  |  |  |  |  |  |
| Lockdown | - | - | - | -0.19 | -0.43 | 0.15 |
|  | - | - | - | (0.31) | (0.32) | (0.66) |
| Post-lockdown | - | - | - | -0.01 | -0.26 | 0.24 |
|  | - | - | - | (0.36) | (0.39) | (0.57) |
| (*Reference = Pre-Lockdown*, *Working Fully Outside)* |  |  |  |  |  |  |
| **Mostly outside x Lockdown** | - | - | - | 0.45 | 0.30 | 0.90 |
|  | - | - | - | (0.68) | (0.56) | (1.45) |
| **Half from home x Lockdown** | - | - | - | 0.96** | 0.49 | 1.01 |
|  | - | - | - | (0.46) | (0.49) | (0.78) |
| **Mostly from home x Lockdown** | - | - | - | 1.59** | 0.99 | 1.92* |
|  | - | - | - | (0.63) | (0.66) | (1.14) |
| **Working remotely (Fully from home) x Lockdown** | - | - | - | 1.38*** | 0.86** | 1.17 |
|  | - | - | - | (0.41) | (0.38) | (0.76) |
|  |  |  |  |  |  |  |
| **Mostly outside x Post-lockdown** | - | - | - | -0.08 | 0.01 | -0.19 |
|  | - | - | - | (0.82) | (0.87) | (1.68) |
| **Half from home x Post-lockdown** | - | - | - | 0.75 | 0.85 | 0.37 |
|  | - | - | - | (0.58) | (0.54) | (0.88) |
| **Mostly from home x Post-lockdown** | - | - | - | 0.37 | 0.75 | -0.29 |
|  | - | - | - | (0.54) | (0.65) | (0.87) |
| **Working remotely (Fully from home) x Post-lockdown** | - | - | - | 0.42 | 0.70 | -0.22 |
|  | - | - | - | (0.47) | (0.46) | (0.72) |
|  |  |  |  |  |  |  |
| Individual FE, Occupation FE, Time FE | Yes | Yes | Yes | Yes | Yes | Yes |
| Occupation FE x Time FE | Yes | Yes | Yes | Yes | Yes | Yes |
| Control variables | Yes | Yes | Yes | Yes | Yes | Yes |
| N | 4308 | 2301 | 2007 | 4308 | 2301 | 2007 |
| *p<0.1 **p<0.05 ***p<0.01 | | | | | |  |
