# Supplementary material for: Unequal gains from remote work during COVID-19 between spouses: Evidence from longitudinal data in Singapore
Source: PLoS One. 2025 May 20;20(5):e0324113. doi: 10.1371/journal.pone.0324113 (PMC12091887; doi:10.1371/journal.pone.0324113)
Supplement: S11 Table — (DOCX) [file pone.0324113.s015.docx]

| **S11 Table. Descriptive Statistics for Labor Market Outcomes by Detailed WFH Categories** | | | | | | |
| --- | --- | --- | --- | --- | --- | --- |
|  | Male | | | Female | | |
|  | Pre-Lockdown | Lockdown | Post- Lockdown | Pre-Lockdown | Lockdown | Post- Lockdown |
| *Outcome variables, disaggregated* | | |  |  |  |  |
| Hourly wages (SGD), for full WFH | 34.95 | 50.17 | 61.37 | 27.83 | 44.83 | 41.57 |
|  | (18.10) | (50.87) | (59.86) | (19.89) | (88.03) | (52.50) |
| Monthly income (SGD), for full WFH | 6275.86 | 6604.27 | 7000.00 | 4410.90 | 4510.60 | 4645.16 |
|  | (3248.60) | (3591.88) | (3704.35) | (2165.03) | (2443.45) | (2761.54) |
| Monthly Hours Worked, for full WFH | 181.92 | 164.36 | 161.30 | 170.43 | 155.11 | 139.87 |
|  | (29.01) | (66.22) | (75.37) | (57.27) | (91.01) | (79.97) |
| Hourly wages (SGD), for partial or non-WFH | 28.19 | 36.28 | 37.20 | 23.38 | 31.01 | 31.08 |
|  | (23.20) | (55.19) | (42.20) | (10.68) | (40.81) | (27.98) |
| Monthly income (SGD), for partial or non-WFH | 4983.26 | 4779.18 | 5627.91 | 3925.17 | 3859.05 | 4322.22 |
|  | (2804.37) | (2817.60) | (3111.57) | (1708.60) | (1785.45) | (2124.79) |
| Monthly Hours Worked, for partial or non-WFH | 190.80 | 180.16 | 185.57 | 170.28 | 164.68 | 171.05 |
|  | (53.38) | (82.82) | (72.77) | (26.16) | (64.88) | (79.64) |
| *Independent variable, disaggregated* | | |  |  |  |  |
| Working Fully Outside (%) | 24.48 | 25.52 | 35.17 | 11.04 | 12.74 | 23.80 |
|  | (43.02) | (43.62) | (47.81) | (31.37) | (33.35) | (42.65) |
| Working Mostly Outside (%) | 8.33 | 9.20 | 17.06 | 5.37 | 6.17 | 10.24 |
|  | (27.66) | (28.92) | (37.67) | (22.57) | (24.07) | (30.36) |
| Working Half from Home (%) | 13.80 | 14.67 | 22.31 | 13.73 | 13.83 | 23.80 |
|  | (34.51) | (35.40) | (41.69) | (34.44) | (34.54) | (42.65) |
| Working Mostly from Home (%) | 15.63 | 16.06 | 15.75 | 13.74 | 15.62 | 23.49 |
|  | (36.33) | (36.73) | (36.47) | (33.20) | (36.32) | (42.46) |
| Working Fully from Home (%) | 37.76 | 34.55 | 9.71 | 56.12 | 51.64 | 18.67 |
|  | (48.51) | (47.57) | (29.65) | (49.66) | (50.00) | (39.03) |
| Total person-wave observations | 768 | 1152 | 381 | 670 | 1005 | 332 |
