# Supplementary material for: Unequal gains from remote work during COVID-19 between spouses: Evidence from longitudinal data in Singapore
Source: PLoS One. 2025 May 20;20(5):e0324113. doi: 10.1371/journal.pone.0324113 (PMC12091887; doi:10.1371/journal.pone.0324113)
Supplement: S12 Table — (DOCX) [file pone.0324113.s016.docx]

| **Table S12. Mediating Role of Household Responsibilities on Hourly Wage, Monthly Income, and Monthly Hours Worked, Detailed** | | | | | | | | | | | |
| --- | --- | --- | --- | --- | --- | --- | --- | --- | --- | --- | --- |
|  | (1) | (2) | (3) | (4) | (5) | (6) | (7) | (8) | (9) | |  |
|  | Y=Hourly Wage | | | Y=Monthly Income | | | Y=Monthly Hours Worked | | | |  |
|  | All | Male | Female | All | Male | Female | All | Male | Female | |  |
| *Panel A: Role of Childcare (minutes/hr)* | | | | | | | | | |  |  |
|  |  |  |  |  |  |  |  |  |  | |  |
| **Remote x Lockdown x Childcare** | -0.16 | -0.75 | 0.13 | -16.44 | 25.95 | -17.92 | 0.70 | 0.82 | 0.95* | |  |
|  | (0.93) | (0.80) | (1.17) | (14.18) | (30.97) | (15.90) | (0.47) | (1.15) | (0.52) | |  |
| **Remote x Post-Lockdown x Childcare** | -0.35 | 0.52 | -0.37 | -25.53* | 7.45 | -27.75 | 0.36 | -0.15 | 1.14 | |  |
|  | (0.94) | (1.35) | (1.22) | (17.16) | (32.13) | (20.26) | (0.93) | (1.58) | (1.16) | |  |
|  |  |  |  |  |  |  |  |  |  | |  |
| Remote x Lockdown | 7.93* | 9.70** | 5.72 | 370.87*** | 298.03 | 210.68 | -12.80*** | -9.42 | -18.79*** | |  |
|  | (4.59) | (4.15) | (6.60) | (140.54) | (228.93) | (168.11) | (4.37) | (6.49) | (6.57) | |  |
| Remote x Post-Lockdown | 16.05** | 13.86* | 14.50 | 474.20** | 514.32* | 307.23 | -34.45*** | -16.46 | -56.24*** | |  |
|  | (6.81) | (8.28) | (9.81) | (182.33) | (305.30) | (228.09) | (10.61) | (13.60) | (16.21) | |  |
| Remote x Childcare | 0.30 | 0.77 | 0.10 | 17.63 | -26.19 | 18.94 | -0.62 | -0.94 | -0.94* | |  |
|  | (0.82) | (0.82) | (1.01) | (14.07) | (29.32) | (16.02) | (0.44) | (1.11) | (0.49) | |  |
| Lockdown x Childcare | 1.00*** | 0.36 | 1.20*** | -8.32 | -44.63* | -5.53 | -1.03*** | -0.81 | -1.62*** | |  |
|  | (0.32) | (0.64) | (0.27) | (5.66) | (24.59) | (4.66) | (0.29) | (0.84) | (0.33) | |  |
| Post-Lockdown x Childcare | 0.90*** | 0.17 | 1.21*** | -11.15 | -47.59* | -3.34 | -1.15** | -0.55 | -2.03*** | |  |
|  | (0.33) | (0.63) | (0.28) | (7.14) | (25.52) | (8.00) | (0.53) | (1.00) | (0.63) | |  |
| Remote | -2.46 | -4.93 | -0.72 | -371.65*** | -303.78 | -208.78 | 8.45 | -1.85 | 23.72** | |  |
|  | (6.37) | (6.08) | (9.63) | (130.28) | (215.44) | (159.81) | (5.65) | (8.17) | (9.44) | |  |
| Lockdown | 5.28* | 9.77*** | 3.23 | -93.35 | -44.55 | -10.58 | -0.89 | -10.01* | 9.52 | |  |
|  | (2.91) | (3.59) | (3.09) | (79.13) | (128.40) | (93.03) | (4.21) | (5.72) | (6.09) | |  |
| Post-lockdown | 4.70 | 8.97** | 1.94 | 163.53 | 292.01* | 103.33 | 9.01 | -2.78 | 22.83*** | |  |
|  | (3.54) | (3.65) | (5.01) | (100.35) | (161.48) | (114.56) | (6.28) | (7.52) | (8.67) | |  |
| Childcare | -1.01*** | -0.36 | -1.24*** | 3.71 | 38.02 | 0.305 | 0.46* | 0.56 | 0.58*** | |  |
|  | (0.32) | (0.62) | (0.24) | (4.77) | (24.65) | (2.77) | (0.26) | (0.93) | (0.21) | |  |
| *Panel B: Role of Chores (minutes/hr)* |  |  |  |  |  |  |  |  |  | |  |
|  |  |  |  |  |  |  |  |  |  | |  |
| **Remote x Lockdown x Chores** | -0.58 | 0.92 | -2.28** | -78.52* | 22.48 | -88.26* † | 0.01 | 0.03 | 2.39 | |  |
|  | (0.97) | (2.08) | (1.00) | (47.35) | (102.78) | (44.89) | (1.77) | (2.64) | (2.26) | |  |
| **Remote x Post-Lockdown x Chores** | -1.63 | 2.72 | -4.14** | -125.99** | -44.03 | -125.98** † | -4.41 | -2.57 | -0.24 | |  |
|  | (2.62) | (8.08) | (1.82) | (61.89) | (127.14) | (58.13) | (5.94) | (7.39) | (7.62) | |  |
|  |  |  |  |  |  |  |  |  |  | |  |
| Remote x Lockdown | 9.44** | 4.95 | 14.88** | 424.63*** | 389.85 | 262.20 | -10.14* | -6.16 | -19.28** | |  |
|  | (4.33) | (5.61) | (7.09) | (151.01) | (252.04) | (170.99) | (5.29) | (6.80) | (7.49) | |  |
| Remote x Post-Lockdown | 18.30*** | 15.36 | 23.16*** | 516.66** | 552.61* | 343.87 | -25.31* | -16.08 | -49.29*** | |  |
|  | (7.00) | (14.44) | (7.97) | (202.65) | (325.13) | (241.20) | (13.60) | (15.92) | (18.57) | |  |
| Remote x Chores | 0.53 | -0.24 | 1.85* | 72.17 | -56.43 | 86.49** | 0.32 | -0.86 | -2.09 | |  |
|  | (0.96) | (1.99) | (0.95) | (46.94) | (106.27) | (43.20) | (1.82) | (2.61) | (2.39) | |  |
| Lockdown x Chores | 0.61 | -1.29** | 0.44 | 5.05 | -2.88 | 2.01 | -0.19 | 0.20 | -1.85* | |  |
|  | (0.47) | (0.59) | (0.45) | (19.80) | (31.82) | (14.36) | (0.85) | (1.07) | (0.94) | |  |
| Post-Lockdown x Chores | 0.28 | -0.11 | 1.28 | -4.16 | -9.90 | 0.66 | -1.33 | -1.34 | -2.99 | |  |
|  | (0.78) | (1.04) | (0.81) | (23.04) | (36.99) | (15.91) | (1.71) | (2.10) | (1.85) | |  |
| Remote | -2.29 | -1.89 | -5.31 | -396.73*** | -310.96 | -247.74 | 4.94 | -3.77 | 22.45** | |  |
|  | (4.63) | (7.43) | (5.03) | (146.56) | (261.06) | (164.31) | (6.43) | (8.69) | (10.29) | |  |
| Lockdown | 10.82*** | 13.43*** | 7.26** | -132.52 | -182.69 | -33.16 | -5.00 | -13.12** | 5.87 | |  |
|  | (3.16) | (4.58) | (2.79) | (88.02) | (118.02) | (103.17) | (4.64) | (5.84) | (5.95) | |  |
| Post-lockdown | 6.85* | 9.25* | 2.17 | 144.73 | 161.97 | 119.75 | 7.55 | -1.47 | 21.27** | |  |
|  | (3.60) | (4.84) | (3.77) | (112.05) | (154.68) | (119.73) | (6.40) | (7.46) | (8.81) | |  |
| Chores | 0.27 | 0.99* | 0.02 | -0.37 | -0.44 | -10.84 | 0.06 | 0.31 | 1.65 | |  |
|  | (0.32) | (0.55) | (0.47) | (0.30) | (32.40) | (13.01) | (0.42) | (1.51) | (1.14) | |  |
| *Panel C: Role of Whether Respondents Receive Any Help for Household Work (1 yes 0 no)* | | | | | | | | | | |  |
|  |  |  |  |  |  |  |  |  |  | |  |
| **Remote x Lockdown x Receiving any help** | 11.72* | 5.48 | 18.11* | 319.80 | 537.84 | 229.92 | 0.03 | 10.56 | -12.43 † | |  |
|  | (6.16) | (7.32) | (9.43) | (227.79) | (363.85) | (257.60) | (8.01) | (10.50) | (11.05) | |  |
| **Remote x Post-Lockdown x Receiving any help** | 2.05 | -13.57 | 15.06 | 581.18* | 574.47 | 644.64* | 30.12 | 18.04 | 37.85 | |  |
|  | (10.63) | (19.90) | (9.72) | (308.33) | (476.32) | (379.75) | (23.81) | (23.61) | (40.53) | |  |
|  |  |  |  |  |  |  |  |  |  | |  |
| Remote x Lockdown | 1,21 | 3.73 | -2.46 | 90.80 | 104.62 | -83.97 | -9.43 | -12.52 | -5.50 | |  |
|  | (3.25) | (4.65) | (4.27) | (173.53) | (280.64) | (196.46) | (5.91) | (8.01) | (8.02) | |  |
| Remote x Post-Lockdown | 13.75 | 28.35* | 2.54 | -92.73 | 147.47 | -407.90 | -55.16** | -30.96 | -77.52** | |  |
|  | (8.62) | (16.53) | (6.92) | (223.39) | (342.28) | (278.16) | -21.45 | (19.05) | (38.78) | |  |
| Remote x Receiving any help | -2.55 | 3.71 | -7.94 | -321.22 | -606.12* | -34.28 | -5.53 | -19.41* | 8.65 | |  |
|  | (5.73) | (8.57) | (6.02) | (207.62) | (347.99) | (263.35) | (9.43) | (11.63) | (14.56) | |  |
| Lockdown x Receiving any help | -2.50 | -1.80 | -6.46 | -72.80 | -119.24 | -93.00 | -4.99 | -10.49 | 2.11 | |  |
|  | (3.36) | (3.92) | (5.69) | (137.72) | (185.81) | (164.45) | (6.60) | (7.85) | (10.34) | |  |
| Post-Lockdown x Receiving any help | -1.49 | 1.46 | -6.51 | -208.21 | -394.98** | -32.43 | -8.25 | -15.91 | -0.45 | |  |
|  | (3.67) | (4.55) | (5.22) | (141.28) | (196.89) | (184.90) | (10.67) | (12.17) | (15.56) | |  |
| Remote | 0.28 | -4.35 | 4.46 | -79.37 | -69.47 | -15.87 | 8.97 | 5.64 | 12.92 | |  |
|  | (4.72) | (7.73) | (4.82) | (156.93) | (289.03) | (197.97) | (8.33) | (9.80) | (13.01) | |  |
| Lockdown | 10.70*** | 11.94*** | 11.96*** | -86.29 | -132.30 | 28.81 | -3.11 | -8.15 | -0.10 | |  |
|  | (2.66) | (3.43) | (3.85) | (98.44) | (132.20) | (120.03) | (5.58) | (6.93) | (8.38) | |  |
| Post-lockdown | 8.55*** | 8.45** | 9.58** | 248.06** | 364.60** | 139.96 | 8.42 | 3.43 | 13.35 | |  |
|  | (2.80) | (3.66) | (3.95) | (107.83) | (147.79) | (137.31) | (9.02) | (9.86) | (13.02) | |  |
| Receiving any help | -8.92 | -5.86 | -14.02 | -151.45 | 16.90 | -300.65 | -0.65 | -4.32 | 3.44 | |  |
|  | (7.99) | (12.84) | (9.31) | (186.23) | (246.43) | (249.12) | (8.22) | (10.37) | (11.90) | |  |
| *Panel D: Role of Whether Respondents have Remotely-Working Spouses (1 yes 0 no)* | | | | | | | | | | |  |
|  |  |  |  |  |  |  |  |  |  | |  |
| **Remote x Lockdown x Spouse working remotely** | 14.40** | 9.12 | 15.70* | 446.46** | 706.87** | 167.06 † | 1.95 | 11.34 | -4.47 † | |  |
|  | (6.03) | (6.36) | (8.55) | (223.62) | (351.66) | (273.34) | (7.91) | (9.66) | (10.73) | |  |
| **Remote x Post-Lockdown x Spouse working remotely** | 22.66* | 29.35* | 11.55 | 579.22* | 990.47** | -18.33 † | 19.18 | 8.77 | 9.29 | |  |
|  | (12.49) | (18.36) | (12.36) | (308.11) | (497.07) | (318.97) | (29.69) | (27.62) | (35.49) | |  |
|  |  |  |  |  |  |  |  |  |  | |  |
| Remote x Lockdown | 0.68 | 1.63 | 0.46 | 57.84 | -21.11 | -0.44 | -8.89* | -11.49 | -8.28 | |  |
|  | (2.88) | (4.06) | (3.85) | (129.20) | (258.03) | (130.63) | (4.77) | (7.14) | (6.11) | |  |
| Remote x Post-Lockdown | 5.28 | 4.45 | 7.48 | -7.91 | -71.41 | -18.83 | -36.30*** | -16.12 | -46.98*** | |  |
|  | (5.53) | (9.60) | (6.72) | (171.87) | (321.25) | (193.96) | (13.09) | (13.54) | (17.44) | |  |
| Remote x Spouse working remotely | -13.68* | -19.52 | -6.78 | 642.15*** | -917.59** | -298.03 | -6.16 | -15.72 | 10.13 | |  |
|  | (6.99) | (10.08) | (8.11) | (239.13) | (398.32) | (274.12) | (13.36) | (14.64) | (18.25) | |  |
| Lockdown x Spouse working remotely | -0.51 | -1.65 | 4.57 | -174.82 | -156.78 | -86.70 | -9.04* | -10.60* | -9.20 | |  |
|  | (3.42) | (4.37) | (4.86) | (124.78) | (161.67) | (192.87) | (5.06) | (6.11) | (8.66) | |  |
| Post-Lockdown x Spouse working remotely | 1.11 | 0.34 | 2.13 | -221.20 | -285.91 | -5.06 | -31.98** | -46.88** | -5.98 | |  |
|  | (4.80) | (5.93) | (6.44) | (166.98) | (220.49) | (229.76) | (14.49) | (18.05) | (20.57) | |  |
| Remote | 5.70 | 8.17 | 3.40 | 26.62 | 94.06 | 61.07 | 6.59 | -0.37 | 11.98 | |  |
|  | (3.79) | (5.88) | (4.45) | (146.43) | (299.16) | (138.74) | (8.26) | (10.49) | (10.70) | |  |
| Lockdown | 9.45*** | 11.58*** | 7.35*** | -64.48 | -127.60 | 3.73 | -2.59 | -9.35* | 3.28 | |  |
|  | (2.81) | (4.27) | (2.67) | (85.13) | (120.30) | (98.54) | (4.36) | (5.13) | (6.05) | |  |
| Post-lockdown | 7.91*** | 10.08** | 5.14* | 207.83** | 239.48* | 161.22 | 9.97 | 5.62 | 13.64 | |  |
|  | (3.02) | (4.43) | (2.93) | (95.79) | (138.43) | (112.92) | (6.69) | (7.54) | (8.45) | |  |
| Spouse working remotely | 4.58 | 3.78 | 2.27 | 195.46 | 267.15 | -18.95 | 11.00 | 24.47** | -8.69 | |  |
|  | (3.79) | (4.81) | (5.60) | (127.02) | (169.63) | (188.28) | (9.76) | (10.96) | (16.59) | |  |
| Individual FE, Occupation FE, Time FE | Yes | Yes | Yes | Yes | Yes | Yes | Yes | Yes | Yes | |  |
| Occupation FE x Time FE | Yes | Yes | Yes | Yes | Yes | Yes | Yes | Yes | Yes | |  |
| Control variables | Yes | Yes | Yes | Yes | Yes | Yes | Yes | Yes | Yes | |  |
| N | 4308 | 2301 | 2007 | 4308 | 2301 | 2007 | 4308 | 2301 | 2007 | |  |

Notes: Panels A-D are run separately because the indicators of household responsibilities are highly collinear. Each component of the interaction terms are also included in the regressions, but are not shown in the table. The reference time period is ‘Pre-lockdown’, between April-July 2018 and December 2019, prior to the COVID-19 pandemic. ‘Lockdown’ refers to March and June 2020, while ‘Post-lockdown’ refers to November 2020, six months after the end of the lockdown. † denotes that the male-female differences in the estimated coefficients are statistically significant (p<0.05). Standard errors, shown in the parentheses, are clustered at the household level. *p<0.1 **p<0.05 ***p<0.01
