# Supplementary material for: Unequal gains from remote work during COVID-19 between spouses: Evidence from longitudinal data in Singapore
Source: PLoS One. 2025 May 20;20(5):e0324113. doi: 10.1371/journal.pone.0324113 (PMC12091887; doi:10.1371/journal.pone.0324113)
Supplement: S13 Table — (DOCX) [file pone.0324113.s017.docx]

| **S13 Table.** **Effects of Remote Work Arrangements on Hourly Wage, Monthly Income, and Monthly Work Hours** | | | | | | | | | |
| --- | --- | --- | --- | --- | --- | --- | --- | --- | --- |
|  | (1) | (2) | (3) | (4) | (5) | (6) | (7) | (8) | (9) |
|  | Y=Hourly Wage | | | Y=Monthly Income | | | Y=Monthly Hours Worked | | |
|  | All | Male | Female | All | Male | Female | All | Male | Female |
| (reference = Pre-Lockdown) | | |  |  |  |  |  |  |  |
| **Working Remotely x Lockdown** | 8.74*** | 6.88* | 9.97** | 308.28*** | 487.28*** | 92.39 | -10.02*** | -7.64 | -13.15*** |
|  | (3.13) | (3.67) | (4.82) | (115.39) | (185.12) | (125.69) | (3.71) | (4.95) | (4.88) |
| **Working Remotely x Post- lockdown** | 14.81*** | 19.40** | 13.25** | 294.41** | 536.94** | 87.64 | -35.19*** | -20.43* | -49.74*** |
|  | (4.80) | (8.69) | (5.37) | (149.60) | (220.02) | (179.49) | (9.46) | (10.97) | (14.97) |
|  |  |  |  |  |  |  |  |  |  |
| Working Remotely (1 yes 0 no) | -2.05 | -2.33 | -2.59 | -305.18*** | -507.96*** | -62.20 | 6.49 | -4.17 | 18.99** |
|  | (3.49) | (5.24) | (4.26) | (113.57) | (193.19) | (122.96) | (5.44) | (7.10) | (9.10) |
|  |  |  |  |  |  |  |  |  |  |
| Lockdown | 8.85*** | 10.24*** | 7.24*** | -160.01** | -247.58** | -7.34 | -5.40 | -11.39** | 0.92 |
|  | (2.50) | (3.82) | (2.17) | (76.40) | (109.36) | (80.85) | (3.74) | (4.56) | (4.99) |
| Post-lockdown | 7.24*** | 8.56** | 4.63* | 91.71 | 82.23 | 151.23 | 4.34 | -2.80 | 13.00* |
|  | (2.71) | (3.94) | (2.80) | (85.56) | (122.13) | (95.95) | (5.46) | (6.12) | (7.32) |
|  |  |  |  |  |  |  |  |  |  |
| Individual FE | Yes | Yes | Yes | Yes | Yes | Yes | Yes | Yes | Yes |
| Occupation FE, Time FE, Occupation-specific Time Trends | No | No | No | No | No | No | No | No | No |
| Control variables | Yes | Yes | Yes | Yes | Yes | Yes | Yes | Yes | Yes |
| Mean Dependent Variable | 35.43 | 37.37 | 33.24 | 4900.88 | 5488.48 | 4227.20 | 172.83 | 180.36 | 164.19 |
| N | 4308 | 2301 | 2007 | 4308 | 2301 | 2007 | 4308 | 2301 | 2007 |
| Notes: Remote is a dichotomous variable for whether the respondent worked fully from home in May 2020 during the COVID-19 lockdown. The reference time period is ‘Pre-lockdown’, between April-July 2018 and December 2019, prior to the COVID-19 pandemic. ‘Lockdown’ refers to March and June 2020, while ‘Post-lockdown’ refers to November 2020, six months after the end of the lockdown. † denotes that the male-female differences in the estimated coefficients are statistically significant (p<0.05). Standard errors, shown in the parentheses, are clustered at the household level. | | | | | | | | | |
| *p<0.1 **p<0.05 ***p<0.01 | | | | | | | | | |
