# Supplementary material for: Unequal gains from remote work during COVID-19 between spouses: Evidence from longitudinal data in Singapore
Source: PLoS One. 2025 May 20;20(5):e0324113. doi: 10.1371/journal.pone.0324113 (PMC12091887; doi:10.1371/journal.pone.0324113)
Supplement: Attachment — Submitted filename: referee_report_mar2024.pdf [file pone.0324113.s018.pdf]

Unequal Gains from Remote Work during COVID-19 between Spouses: Evidence from Longitudinal Data in Singapore

Referee report

In this paper the authors conducted an ad hoc survey targeting married women in Singapore, for whom they collect longitudinal information at 6 points in time (from 2018 to November 2020) on both themselves and their male partners. The aim is to analyse the effect of the COVID-19 pandemic and the subsequent period on monthly wages and to see if there have been differences in the effects of the pandemic between teleworkers and non-teleworkers across genders. The authors found a significant salary income growth among remotely-working male spouses, but not among female spouses.

The paper addresses a relevant topic related to the effects of the COVID-19 pandemic on wages and gender inequality. It is an interesting paper, and it is, overall, well written.

I have several comments related to the organisation of the paper and the need for clarification of some aspects. The motivation in the introduction should also be improved. There are aspects that in my opinion are key to properly motivate the article (such as telework figures in Singapore, the effects of telework on productivity, etc. ....) that should be explained in the Introduction (see my specific comments below).

Despite having a general favourable opinion of the paper, my main concern lies with the estimation results. Firstly, the authors analyse monthly salaries, but they do not control for the number of working hours despite having information on this issue. Secondly, it is no clear if the analysed individuals are employed consistently across all periods. For me, it would be surprising that all individuals and their partners were consistently employed throughout. Third, the definition of teleworkers is very rigid (only those workers who fully work from home), which implies that their main explanatory variable (working from home) is almost zero before the pandemic. Lastly, there are some inconsistencies between the coefficients obtained in the estimations for the total sample (pooled) and the separate estimations for men and women (see for example the coefficient of the variable "lockdown" in the three samples in Table 4). The coefficient is negative for the pooled sample (implying that during the lockdown the monthly wages of non-teleworkers decreased). However, when separate estimates are made for men and women, the coefficient is positive for both, indicating just the opposite. There can also be observed several inconsistencies for other coefficients. For these reasons, I believe that the authors should carefully review the regression results. I therefore consider that the article requires major revisions.

MAIN COMMENTS (ORGANIZED FOLLOWING THE MANUSCRIPT STRUCTURE):

INTRODUCTION

1) In the introduction I miss some figures (based on official statistics) on telework in Singapore before and after the pandemic, differentiating by gender.
2) Although explained on page 14 of the manuscript, it would be useful to mention also in the introduction that the authors exploit a non-official ad-hoc survey approved by the National University of Singapore Institutional Review Board. Additionally, the purpose of the survey should be stated.

3) In the introduction, it should be specified that the salary income is measured on a monthly basis. I guess that the observed increase 'in monthly salary' reported by the authors is related to an increase in the number of working hours (that authors do not explain nor mention).
4) On page 4, last paragraph, the authors state, as an additional contribution of their article, the fact that "The current literature on the labor market impact of Covid19 primarily relies on evidence from Western or larger economies. Such evidence may not apply to non-Western smaller economies given the international heterogeneity in the institutional responses to Covid19, extents of adoption of remote work, and sociocultural contexts". I suggest adding a short sentence highlighting the main differences between Western and non-Western countries in the fight against COVID-19.
5) Figure 1 is mentioned on page 5, last paragraph, and the authors say that by the fourth quarter of 2020 the economy had almost bounced back to pre-pandemic activity levels ("By the last quarter of 2020, the economy had almost bounced back to pre-pandemic activity levels, which coincided with a steep decline in number of daily confirmed cases to mostly single digits (see Figure 1)". However, looking at Figure 1, it appears that the growth rate of GDP in the fourth quarter of 2020 is negative (could it be a visual effect on the horizontal axis?). I assume that the growth rates shown in Figure 1 are year-on-year growth rates, but it would be useful to make this explicit in the figure. In addition, it would be useful to add employment growth, differentiated by gender.

SECTION 2 – BACKGROUND

6) I find it a bit confusing to mix the background section, which is usually a literature review, with aspects of the specific contribution of the article as well as the hypothesis statement of the paper. I suggest revising this section so that it is more standard with what is usually done in academic articles. A simple option would be to change the title of Section 2. For me, it would be more natural (it is a personal taste) to focus the background section on literature review and specify the hypotheses in the Estimation section, before the equation 1, to better link how the three hypotheses of the article will be tested through equation 1.
7) On page 8, the authors say "In case of Singapore, based on the qualitative evidence that the share of respondents who reported productivity increases following the introduction of remote work rose from 15.1% in April 2020 to 22.5% in June 2020, and the share of respondents who were concerned about productivity declines fell from 78% to 40%". In my opinion this should be in the Introduction to support the motivation of the article. Also, in relation to Hypothesis 1, the authors should explain the mechanism by which they assume that teleworking implies an improvement in wages. This should be explained more clearly.
8) On page 9, first paragraph, the labour market in Singapore is partially discussed. Personally, I do not know the characteristics of the labour market in Singapore. I think that for the international reader, especially those from western countries, it would be very useful to provide some figures on the labour market from a gender perspective in Singapore. What is the employment rate of men and women? How is employment distributed between full-time and part-time between men and women? It would also be useful to show a table, even if only in the appendix, with the distribution of employment by occupations and industries differentiated by gender, in order to see which occupations and industries are more feminised in the case of Singapore.
9) As the authors partially mention, women were more likely to leave employment or reduce hours worked than men. This is a phenomenon that has been noted especially for the literature

based on western countries. Did this happen in Singapore as well ? Can the authors provide some figures to contextualise this phenomenon for Singapore? How did the fall in GDP affect the employment of both men and women?

10) A personal taste: I find the transition to section 3 a bit rough. Perhaps it would be appropriate to end with a few brief lines to give way more naturally to the data section.

SECTION 3 – DATA AND METHODS

11) When explaining the database, it is necessary to specify that the survey is based on ad-hoc survey (I imagine that was conducted by the researchers in the context of a research project endorsed by the National University of Singapore). Additionally, it is necessary to specify the purpose of the survey. Given that the survey started in 2018 it was impossible to know at that time that there was going to be a pandemic in 2020. What was the original objective of the survey?
12) It is important to describe briefly the type of information collected in the survey.
13) As the face-to-face surveys in 2018 were conducted on the street and only among women indicates (I imagine) that there is a proportion of female respondents who are NOT working. Were the surveys conducted during "non-working" hours? The authors state that the criteria for being surveyed are "Participants met the following inclusion criteria: currently married in 2018; either a Singaporean citizen or married to a Singaporean citizen, and able to read, write and speak in English". Therefore, being employed is not included as a criterion. Given that the survey collects wage information, were all female respondents employed in 2018? How is the distribution of the sample in terms of percentage of employment compared to what is observed in Singapore's official statistics? Were the husbands also employed?
14) In Table 1, it is clear that the first interview was face-to-face, but the verbal explanation on page 12, last paragraph, should also be stated.
15) I consider that the elaboration of an ad-hoc survey is in itself a contribution of the article and the fact that the authors conduct a survey is because the type of information collected in official surveys in Singapore is not sufficient for the purposes of the article. I do not know the characteristics of official surveys in Singapore, but at least in European countries the type of information collected by the authors is included in labour force surveys and other surveys such as living conditions surveys. Could the authors explain what kind of additional information their survey provides compared to official statistics in Singapore?
16) On page 13, second paragraph, the authors say “Waves 3 and 4 included retrospective questions which collected information from periods before and shortly after the lockdown”. However, looking at Table 1, I wonder if it could be a typo since Table 1 says that information in the Wave 3 is retrospective. Also, Table 1 states that the data for May 2020 is "actual", while the text states that it is retrospective.
17) **Dependent variable.** My main concern comes from the dependent variable because it measures the monthly wage and not the hourly wage. Given that women may have decided (voluntarily or not) to reduce their working hours due to the pandemic, it would be rational to observe a decrease in their monthly wages (because they work fewer hours). How have the hours worked by men and women changed after the pandemic in Singapore according to official statistics? I think it would be important to make a differentiated analysis for full-time and part-time workers. I guess that the survey collects information on hours worked (Table A3 in the appendix). Information on hours worked is essential for the analysis carried out by the

authors. Additionally, to contextualise the salary it would be important to provide the average salary of men and women in Singapore (according to official statistics).

18) **It is not clear what happens as regards employment situation across the 6 waves**. Reading the paper, it seems that during the 6 waves all the people surveyed remain employed, but this would be surprising, even in times of economic expansion there are always flows in the labour market which mean that in certain periods people are not employed. How is it possible to collect monthly wages for 6 periods for married women and their husbands? Are both of them employed during all 6 periods?

19) **Classification of teleworkers.** The authors define a teleworker as a worker who works fully from home. Because of this definition, the percentage of teleworkers before the lockdown is so low (close to zero in 2018, Figure A1). In my opinion, this is a very rigid definition. Do the results hold when partially relaxing the definition of teleworkers (including at least those who report working mostly from home)?

20) The authors say that during the pandemic, there were people who were able to hold multiple jobs. What percentage of people in the survey are affected by this situation? What is the incidence of moonlighting in Singapore according to official statistics?

21) The essential or non-essential nature of the industry where workers are employed is a very relevant variable for the study of the economic consequences of the pandemic on the labour market. However, the database does not include information on industries. I guess that this is because the questionnaire was originally designed for other purposes before the pandemic (in 2018). While the authors recognize this lack of information, they should put more effort to explain why they consider that not having information on industry would not affect their estimates. Is there any paper specifically addressing the case of Singapore that supports this statement?

22) The authors classify occupations as “professionals” and "non-professional”. I guess that this classification is made to differentiate between teleworkable and non-teleworkable occupations. In my opinion, technicians are also teleworkable occupations.

23) Analysed sample: **The analysed sample is unclear.** The authors say that their final sample consists of 4638 observations, involving 781 people (362 women and 419 men). I find the description of the analysed sample very confusing as on page 12 they say that the potential participants are 3038, of which 657 people finally met the criteria to participate. It is understood that the people who finally fill in all the waves are 378 (which I understand are women and they report information about their male spouses). If women report information about men, how is it possible that there is more information from men (419) than from women (362)?

24) Since this is an ad-hoc survey, it is very relevant to present information on whether or not the sample is representative of the total population. This is done in Table A2 (Comparison of Baseline Characteristics in the Sample and National Statistics of Married Resident Mothers Aged 30-34). However, it is striking that it compares only its sample of 25-59 year olds with the population aged 30-34. Such a table should compare similar age groups.

25) Table 2. It would be necessary to show the data displayed in Table 2 for the 3 periods. It is also necessary to include the number of hours worked in each of the periods.

RESULTS

26) When analysing the results displayed in Table 4 (DID model estimates) it would be useful to start by commenting on the independent coefficients of “working remotely”, “lockdown” and “post-lockdown”, and then comment on the corresponding interactions terms.
27) However, **I find the estimation results in Table 4 rather strange.** For example, the coefficient of the “lockdown” variable is 189.070 for the male sample, 116.632 for the female sample, but the corresponding coefficient for the pooled sample is -50.246. How can the authors explain this difference?
28) I also find striking the coefficient of "working remoteley". Figure 2 implies that the monthly wage of teleworkers is higher than the wage of non-teleworkers (before and after the pandemic). However, in the estimates in Table 2, the coefficient of "working remotely" (which measures the difference between teleworkers' and non-teleworkers' wages in the pre-pandemic period), is negative for both men and women. I understand that once the other explanatory factors are controlled for, the sign may change, but this is something the authors should explain adequately in the manuscript.
29) The authors say that “In our total sample (column 1), we see that fully remote work arrangements under lockdown is associated with an increase in monthly earned income by $245.60. The income further increases by $397.02 even after the lockdown is lifted (i.e., November 2020).” What is the theoretical explanation that causes this wage increase? I guess that this finding is related to the number of working hours, but the authors do not explain this result.
30) On the other hand, the effect of the lockdown for teleworkers is the sum of the “lockdown” coefficient plus the interaction term with teleworking (-50 + 245) while for non-teleworkers the effect of the lockdown is just the “lockdown” coefficient (-50). Thus, the pandemic has caused an increase of teleworkers’ wages and a decrease for the non-teleworkers. Again, I think this result is related to the number of working hours. Teleworkers were able to work during the lockdown and non-teleworkers probably reduced the number of working hours (or even do not worked at all) due to the mandatory closures.
31) The authors claim “Time fixed effects are in line with macroeconomic data showing a large dip in economic activity during the lockdown before recovering in November 2020”. However, this statement is not seen in Table 4. For the pooled sample, the “lockdown” coefficient is negative, which would be in line with the previous statement, but for the separate samples, the coefficient of “lockdown” indicates the opposite, as I have mentioned above.
32) Since it is necessary to revise estimations corresponding to the results presented in Table 4, **it would also be necessary to revise the estimates presented in Tables 5 to 7.**

MINOR COMMENTS:

- Homegeneise the expression Covid19. In some cases it appears as COVID-19 and in others as Covid19.
- Page 17. It says Figure 1, it should be Figure A1 (online Appendix).
