# Supplementary material for: Unequal gains from remote work during COVID-19 between spouses: Evidence from longitudinal data in Singapore
Source: PLoS One. 2025 May 20;20(5):e0324113. doi: 10.1371/journal.pone.0324113 (PMC12091887; doi:10.1371/journal.pone.0324113)
Supplement: Attachment — Submitted filename: referee_report_sub3_oct2024.pdf [file pone.0324113.s022.pdf]

Unequal Gains from Remote Work during COVID-19 between Spouses: Evidence from Longitudinal Data in Singapore

Referee report

October 2024

The manuscript has improved from the previous version, and the authors have addressed the majority of my comments and suggestions. I thank the authors for their detailed responses. I have some additional comments, clarifications, and corrections of several inconsistencies that require further revision.

<u>**Table 2**</u>:

As I suggested in my previous report, the most relevant information for the paper's objectives should appear in the main text. In particular, part of the information that is now displayed in the new Table S10 and Table S9 should be included in Table 2. I agree that Table 2 is too long, but in my opinion the numbers in parenthesis (are they SD? it is not indicated what they are) could be eliminated. It is not usually to display the SD for frequencies. Logically, this comment does not apply to the endogenous variables. Hence, the endogenous variables should appear disaggregated for teleworkers and non-teleworkers (the first 6 rows from Table S10). Also, the time dedicated to childcare and chores should appear disaggregated for teleworkers and non-teleworkers.

The explanation offered by the authors as regards the decrease in the percentage of teleworkers in the lockdown period and the post-lockdown period is not convincing. There is not only a switch from "Working Fully from Home" to "Working Mostly from Home", but the most preoccupant part is that their data show an increase in the percentage of workers who declare "working Fully outside" or "mostly outside" (from 32.81% in the pre-lockdown period to 34.72% in lockdown and 52.23% in post-lockdown among males). Something similar occurs for females since the percentage of women who declare "working fully outside" or "mostly outside" increases from 16.41% in the pre-lockdown period to 18.91% in the lockdown and 34.04% in the post-lockdown period. This characteristic of their sample selection should be indicated as an additional limitation of their conclusions. Additionally, the authors start their motivation in the abstract with the sentence, "The COVID-19 outbreak and the rise of remote work generated distinct labor market outcomes for workers based on their occupation and the ability to adapt to changes", when their data show precisely the contrary pattern.

The decrease in the percentage of people working from home is coherent with the occupational changes observed during the lockdown and the post-lockdown periods. In their data, the percentage of professionals and associates professionals (the most teleworkable occupations according to the international empirical evidence) decreases during the lockdown and the post-lockdown period. In contrast, the percentage of service workers clearly increases. Once more, this characteristic of their sample selection is contrary to the empirical evidence of the pandemic on the labour market: the pandemic provoked employment losses in non-teleworkable occupations. Could it be a problem of attrition in their database? The authors claim that they

exclude individuals who were not employed in any of the 6 waves from the panel. Does the same apply to “missing individuals”? In that case, it could be feasible that some individuals who have been excluded from the panel are employed individuals (maybe teleworkers) who were not available to respond to the survey.

I miss a brief explanation (similar to what the authors explained in the responses to my previous report) regarding the huge increase in the percentage of women having an infant aged two years or younger (from 1% to 40% during lockdown/post-lockdown).

On page 24, the authors claim that “For instance, while similar shares of men and women were in professional (about 80%) and associate professional (10%) jobs, more women were in clerical jobs (8% as opposed to 2% among men) which could be easily adopt full remote work arrangements.” This statement is not true since the international literature has shown that professionals and associate professionals are more teleworkable than clerical workers.

As regards the comments in Table 2, it should be noted that the gender gap in the time devoted to childcare has been reduced during the lockdown and the post-lockdown, while the gender gap in the time devoted to chores has increased.

Finally, I consider that Table 2 should contain tests of differences in means between males and females.

Explanatory variables:

Page 27: After reading the explanation of the independent variables included in the vector Xit, it seems that it includes industries. However, in the conclusions section, the authors clarify that they do not have information on industry. This aspect should also be clarified in the description of the Xit vector.

Results:

There are some inconsistencies in the interpretation of the results. As I indicated in my second report, the interpretation of the “Remote work” coefficient in Table 4 is not correct. Since the reference category in estimates is the pre-lockdown period, the coefficient of “remote work” measures the difference in hourly wages, monthly income, and monthly hours worked between teleworkers and non-teleworkers in the pre-lockdown period. Please revise the comments on this coefficient.

The interpretation of the coefficients of the interaction terms (third paragraph on page 33) seems somewhat confusing. In my opinion, it would be more precise to start the explanation with the authors' comments in the second paragraph on page 34, where they correctly interpret the magnitude of the effect of the lockdown (and the post-lockdown) on teleworkers and non-

teleworkers. Attending to the authors' correct statement on page 34, during the lockdown period, male WFH workers experience income gains of $204.93 (and $596.46 during post-lockdown), while non-WFH workers experience income losses by -$182.50 (and income gains by $150.04 in the post-lockdown). The interaction term "Working remotely x Lockdown" allows to test the null hypothesis that the effect of the lockdown does not depend on the type of workers (teleworkers vs. non-teleworkers). The coefficient of the interaction term tests whether the lockdown has had a differential effect on teleworkers and non-teleworkers. Hence, the value of the interaction term ($387.43 for men) measures the (statistically significant) differential effect of the lockdown on the monthly wages between male teleworkers and non-teleworkers ($204.93 - (-$182.50)=$387.43). Comments on other interaction terms and those in Table 5 have to be revised in the same line.

On page 38, the authors claim, “This was consistent with the pattern seen in Table 4, once again suggesting the sub-par nature of jobs that were done from home before COVID-19 and the global popularization of remote work”. This explanation should be indicated when analysing the results in Table 4.

Regarding Table 6, the authors indicate that “The standalone components of the 3-way interaction were included in the estimations but were not shown”. The table, including the 3-way interactions, should be displayed as a supplementary table.

On page 38, the authors highlighted, “Interestingly, among women, those working mostly outside also experienced a rise in monthly income during the lockdown.” Nevertheless, the coefficient is not statistically significant, so they cannot conclude the above statement.

On page 44, in the discussion section, it is not clear where the figures for “the increased monthly income generated by adopting WFH arrangements under lockdown/post-lockdown constitutes 8.2-9.5% growth for men while comprising mere 1.2-1.5% (statistically insignificant) growths for women” come from.

On page 46, the authors state that “Despite the above-mentioned differences across gender, the mediator analyses lends support for *Hypothesis 3* regarding the role of chore burdens, availability of external help for housework, and availability of WFH spouses on dictating the extent of income and productivity(wage) gains of the remotely working individuals.” It would also be convenient to indicate that they do not find evidence regarding the role of childcare.

Among the study's limitations, it should also be recognized that the sample is underrepresented in terms of employment.

Other comments:

- It is necessary to homogenise the monetary units across the paper, which appear as SGD, S$, or $.
- On page 22, I guess there is a typo: substitute "they were fully WFH: For both genders," with "they were fully WFH. For both genders,"
- Review the web links in the references list. For instance, the link https://www.ilo.org/resource/brief/working-home-estimating-worldwide-potential does not exist any more.
- The authors refer to references 8 and 10 on page 3. I am afraid that the reference 10 (A D Michael;Groen,Jeffrey. Bureau of Labor Statistics. [cited 2024 Apr 26]. Telework during the COVID-19 pandemic: estimates using the 2021 Business Response Survey : Monthly Labor Review: U.S. Bureau of Labor Statistics) does not say nothing about Singapore.
- The wording of the last sentence in the first paragraph on page 7 is confusing. I guess there is a typo, and I wonder if it should be two sentences instead of one.
- The dates of the second interview on page 15 (second paragraph) are inconsistent: May 2020 vs. May-June 2020.
- Page 17: Substitute "61.44% of the total of 3,960 females" for "61.44% of the total of 3,960 female person-wave observations".
- It should be indicated under Tables which statistical test is used to contrast the equality of the coefficients of the male and female subsamples.
- On page 41, indicate panel A, panel B, and panel C when commenting on the results from Table 6 (the word "panel" is missing in some of the parenthesis).
