# Supplementary material for: Unequal gains from remote work during COVID-19 between spouses: Evidence from longitudinal data in Singapore
Source: PLoS One. 2025 May 20;20(5):e0324113. doi: 10.1371/journal.pone.0324113 (PMC12091887; doi:10.1371/journal.pone.0324113)
Supplement: Attachment — Submitted filename: referee_report_20feb2025.pdf [file pone.0324113.s024.pdf]

Unequal Gains from Remote Work during COVID-19 between Spouses: Evidence from Longitudinal Data in Singapore

Referee report

February 2025

The manuscript has improved from the previous version, and the authors have addressed the majority of my comments and suggestions. I thank the authors for their detailed responses. I have some additional comments, clarifications, and corrections of several inconsistencies that require further revision.

Main comments:

1) In the descriptive data in Table 2, the authors should comment that in pre-lockdown, lockdown and post-lockdown, WFH workers earn more than non-WFH workers (both in terms of monthly salary and hourly wage). This analysis is especially relevant because the estimates in Table 4 show the opposite result. According to the estimates in Table 4, before the lockdown period WFH workers earned less than non-WFH workers. Authors should provide an explanation for this contrary result between the descriptive analysis and their estimates.

2) Due to the above comment, I am concerned about the econometric model specification (equation 1, page 27) that has significant consequences for interpreting the results. I am afraid there is a misunderstanding in interpreting the coefficients of the “lockdown” and “post-lockdown” variables. Apart from the “lockdown” dummy (equal to 1 for waves 3, 4, and 5) and the “post-lockdown” dummy (equal to 1 for wave 6), the estimates include time-fixed effects, occupation dummies, and the interactions between occupation and time-fixed effects. Regarding time-fixed effects, I guess the authors have included 5 dummies for the 6 waves, and the reference category is wave 1 (April-July 2018). If this is the case, the “lockdown” coefficient would measure the differential only against the first wave and not against the entire pre-lockdown period, as the authors point out. But additionally, since the “lockdown” dummy includes 3 waves (3, 4, and 5), the coefficient of “lockdown” measures the difference between wave 4 and wave 1 (because part of the effect of the lockdown is captured by the time-fixed effects that are not displayed in Table 4). Furthermore, the model includes interactions between occupation (4 categories?) and time-fixed effects (they do not indicate which category is the reference in estimates; I guess that is the first one, “Professionals”). These interaction terms make the interpretation of the coefficients even more complicated, since now, the reference category of the occupation also conditions the reference category for the period. The authors must clarify this point. If my interpretation were proper, all comments should be reviewed.

3) As I indicated in my previous report, it is also important that authors carefully review the interpretation of the interaction terms as there continue to be inconsistencies in their comments. I point out a single example, but it is necessary to review all of them. Page 46: “In the post-lockdown period, female WFH workers’ monthly hours dropped by as much as 49.34 hours/month”. According to estimates showed in Table 4, in the post-lockdown period, female WFH workers’ monthly hours dropped by as much as 36.49 hours/month, and an increase in hours work of 12.85 for female non-WFH. Hence, in the post-lockdown period the difference

between WFH workers y non-WFH workers is -49.34 hours/month. Logically, this interpretation would also need to change if my second comment were right.

4) Page 43: The authors compare their estimated coefficients with the official mean wages for men and women in Singapore (SGD $4,719 and SGD $4,374, respectively). This is not correct, their coefficients are only comparable with the average values of their data, which, as the authors themselves point out, are not comparable with the total universe because their data is a subsample of married men and women.

Other comments:

5) Page 3. Indicate the source for the reported data for Netherlands (47.4%), Denmark (53.0%), and U.S. (50%).
6) Page 4. Substitute “mediating roles of housework” by “mediating roles of household responsibilities” in the sentence “Lastly, we explore potential explanations for these gender differences by exploring the mediating roles of housework.”
7) Page 7. Substitute “the rate of deceleration of GPD slowed down” by “the rate of decline of GPD slowed down”.
8) Page 7, Figure 1. Labels in horizontal axis in panel A and panel B should be homogenised. In addition, the numbers in vertical axis appear with % in panel A but not in panel B.
9) Page 15, first paragraph. Delete “hourly wages” in “(monthly salary income, monthly work hours, hourly wages)” (the questionary does not ask about hourly wages).
10) Page 16. The authors claim that they interviewed 660 persons, and then they dropped 5 persons that were not married. Then, they drop those not employed persons across the six waves. If the number of women in the final sample is 335, this implies that they dropped 320 women (49% of 655). The final sample for men is 384, meaning they dropped 271 men (41% of 655). Nevertheless, in the Conclusions section, the authors claim that “the number of respondents we dropped from our sample due to unemployment in 2020 comprised only 8.3% (360 person-wave observations across six survey waves, or 60 unique individuals)of the total sample of 4,308 person-wave observations.”
11) Page 27, equation 1. The variables Lockdown and Postlockdown should not include temporal subscripts.
12) Page 25. Typo. “another: A plurality” should be “another. A plurality”.
13) Page 52. Typo: Include a space in “60 unique individuals)of”
