# Supplementary material for: Unequal gains from remote work during COVID-19 between spouses: Evidence from longitudinal data in Singapore
Source: PLoS One. 2025 May 20;20(5):e0324113. doi: 10.1371/journal.pone.0324113 (PMC12091887; doi:10.1371/journal.pone.0324113)
Supplement: Attachment — Submitted filename: Response to Reviewers - R&R1st_final.docx [file pone.0324113.s021.docx]

**RESPONSE TO REVIEWERS**

PLOS ONE

Dear Editor,

Many thanks to you and the reviewers for helpful comments on the paper, Ref: **PONE-D-24-02155**, “Unequal Gains from Remote Work during COVID-19 between Spouses: Evidence from Longitudinal Data in Singapore.” In order to address accommodate all comments and suggestions, we have undertaken major revisions to all sections of the paper.

**Academic Editor: José Alberto Molina**

*Thank you for submitting your manuscript to PLOS ONE. After careful consideration, we feel that it has merit but does not fully meet PLOS ONE’s publication criteria as it currently stands. Therefore, we invite you to submit a revised version of the manuscript that addresses the points raised during the review process. Please include the following items when submitting your revised manuscript:*

*-A rebuttal letter that responds to each point raised by the academic editor and reviewer(s). You should upload this letter as a separate file labeled 'Response to Reviewers'.*

*-A marked-up copy of your manuscript that highlights changes made to the original version. You should upload this as a separate file labeled 'Revised Manuscript with Track Changes'.*

*-An unmarked version of your revised paper without tracked changes. You should upload this as a separate file labeled 'Manuscript'.*

*-If you would like to make changes to your financial disclosure, please include your updated statement in your cover letter. Guidelines for resubmitting your figure files are available below the reviewer comments at the end of this letter.*

*1. When submitting your revision, we need you to address these additional requirements.*

*Please ensure that your manuscript meets PLOS ONE's style requirements, including those for file naming. The PLOS ONE style templates can be found at* 
[*https://ddec1-0-en-ctp.trendmicro.com:443/wis/clicktime/v1/query?url=https%3a%2f%2fjournals.plos.org%2fplosone%2fs%2ffile%3fid%3dwjVg%2fPLOSOne%5fformatting%5fsample%5fmain%5fbody.pdf&umid=d595e888-7493-410c-a420-0c83e1f209b7&auth=8d3ccd473d52f326e51c0f75cb32c9541898e5d5-f7be46a4b3c543781ac30e939380323191ae18e3*](https://ddec1-0-en-ctp.trendmicro.com:443/wis/clicktime/v1/query?url=https%3a%2f%2fjournals.plos.org%2fplosone%2fs%2ffile%3fid%3dwjVg%2fPLOSOne%5fformatting%5fsample%5fmain%5fbody.pdf&umid=d595e888-7493-410c-a420-0c83e1f209b7&auth=8d3ccd473d52f326e51c0f75cb32c9541898e5d5-f7be46a4b3c543781ac30e939380323191ae18e3)

*and* 
[*https://ddec1-0-en-ctp.trendmicro.com:443/wis/clicktime/v1/query?url=https%3a%2f%2fjournals.plos.org%2fplosone%2fs%2ffile%3fid%3dba62%2fPLOSOne%5fformatting%5fsample%5ftitle%5fauthors%5faffiliations.pdf&umid=d595e888-7493-410c-a420-0c83e1f209b7&auth=8d3ccd473d52f326e51c0f75cb32c9541898e5d5-87faa588aaa1d911ceaada18a13f693781898e26*](https://ddec1-0-en-ctp.trendmicro.com:443/wis/clicktime/v1/query?url=https%3a%2f%2fjournals.plos.org%2fplosone%2fs%2ffile%3fid%3dba62%2fPLOSOne%5fformatting%5fsample%5ftitle%5fauthors%5faffiliations.pdf&umid=d595e888-7493-410c-a420-0c83e1f209b7&auth=8d3ccd473d52f326e51c0f75cb32c9541898e5d5-87faa588aaa1d911ceaada18a13f693781898e26)

**Response:**

We have made sure our manuscript meets the LOS ONE’s style requirements.

*2. Thank you for stating in your Funding Statement: 
This project was supported by funding from the Lee Kuan Yew School of Public Policy, National University of Singapore [R-603-000-347-115; A-0003976-00-00; R-603-000-237-133].*

*Please provide an amended statement that declares all the funding or sources of support (whether external or internal to your organization) received during this study, as detailed online in our guide for authors at https://ddec1-0-en-ctp.trendmicro.com:443/wis/clicktime/v1/query?url=http%3a%2f%2fjournals.plos.org%2fplosone%2fs%2fsubmit%2dnow&umid=d595e888-7493-410c-a420-0c83e1f209b7&auth=8d3ccd473d52f326e51c0f75cb32c9541898e5d5-818317a14a2838fce05fa09144a6ff017a57711d.  Please also include the statement “There was no additional external funding received for this study.” in your updated Funding Statement. 
Please include your amended Funding Statement within your cover letter. We will change the online submission form on your behalf.*

**Response:**

We have revised the funding statement as follows:

This project was supported by three distinct *Start-up Grants at LKY School of Public Policy, National University of Singapore [Internal].*

- *A-0003976-00-00: Used to purchase analytical software programs that enabled data analyses and to finance the publication fees.*
- *R-603-000-347-115*: Used for data collection (primary source)
- *R-603-000-237-133*: Used for data collection

There was no additional external funding received for this study.

*3. In the online submission form, you indicated that The raw data for this study cannot be made publicly available due to restrictions on use of collected data under the IRB protocol. Aggregated data will be available (contact Corresponding Author via email: zeewan.lee@nus.edu.sg) for interested researchers. All PLOS journals now require all data underlying the findings described in their manuscript to be freely available to other researchers, either a. In a public repository, b. Within the manuscript itself, or c. Uploaded as supplementary information.
This policy applies to all data except where public deposition would breach compliance with the protocol approved by your research ethics board. If your data cannot be made publicly available for ethical or legal reasons (e.g., public availability would compromise patient privacy), please explain your reasons on resubmission and your exemption request will be escalated for approval.*

**Response:**

Thank you. We would like to seek an exemption to the data sharing policy as the public deposition of data will breach compliance with the protocol approved by the National University of Singapore’s IRB (Ref #: LN-17-048). More specifically, our IRB protocol precludes us from publicly releasing the dataset due to sensitive data collected including dates/frequency of sexual activity and fertility. But we would be happy to share anonymized, aggregated data with masked sexual activity/fertility variables with researchers upon request. We have revised the data sharing statement.

*4. When completing the data availability statement of the submission form, you indicated that you will make your data available on acceptance. We strongly recommend all authors decide on a* ***data sharing plan*** *before acceptance, as the process can be lengthy and hold up publication timelines. Please note that, though access restrictions are acceptable now, your entire data will need to be made freely accessible if your manuscript is accepted for publication. This policy applies to all data except where public deposition would breach compliance with the protocol approved by your research ethics board. If you are unable to adhere to our open data policy, please kindly revise your statement to explain your reasoning and we will seek the editor's input on an exemption. Please be assured that, once you have provided your new statement, the assessment of your exemption will not hold up the peer review process.*

**Response:**

Please see our response to Q3 above. We have revised the data sharing statement.

*5. Please include captions for your Supporting Information files at the end of your manuscript, and update any in-text citations to match accordingly. Please see our Supporting Information guidelines for more information:* [*https://journals.plos.org/plosone/s/supporting-information*](https://journals.plos.org/plosone/s/supporting-information)*.*

**Response:**

We have updated the Supporting Information files and revised the captions.

**Reviewer #1**

*The paper examines whether the gains and losses from adopting WFH on monthly income vary by gender using the mandated lockdown as an exogenous shock. Employing the difference in differences estimator, the authors find that the lockdown increases earned income for men who work from home, but not for women. They argue that these gender differences may come from the differential childcare and housework burden.*

**Response:**

Your comments have been tremendously helpful in improving the paper. We have endeavored to address all your comments, and will be happy to address any remaining comments or suggestions that you may have.

*MAJOR POINTS*

*R1C1. I was wondering if data contain information on hours worked because it is crucial when we interpret the results.*

**Response:**

Yes our data has information on actual hours worked per week—as reported by respondents.

*R1C2. With this information, we can understand whether income gains come from an increase in wage rate or in hours worked. At the end of section 2.1, Hypothesis 1 seems to be based on the idea that WFH improves productivity, which should have positive impacts on income through wages. However, the story would be different if it comes from a change in hours worked, which may be more consistent with the story about the within-household division of housework. These should be clearly mentioned and investigated if data is available.*

**Response:**

We have now re-run all estimations so that we show the impact of WFH on monthly income, monthly hours worked, and hourly wage. As shown in **Table 4** in the manuscript, we find that the income increase (columns 4-6) for the WFH workers is driven by the increased wage rate (columns 1-3)—and not by increased in work hours. The work hours for WFH respondents actually dropped—a lot more steeply and significantly for women—while they increased for non-WFH respondents (columns 7-9). Of note, the wage gain of WFH respondents is not a result of the increased demands for remotely-conducted occupations—as we have controlled for any changes in the occupation-specific demands by including occupation-specific time trends in all estimations. Changes in productivity/performance seem to be what is driving the change in the hourly wage of the WFH respondents. We have updated all tables in the manuscript to display three outcome variables (monthly income, monthly hours worked, and hourly wage) and revised the **Estimation (Section 4**), **Results (Section 5**), and **Discussion (Section 6**) accordingly.

*R1C3. The results in section 5.2 state that WFH increases income for men but not for women relative to non-remote workers. However, based on the observation in Figure 2, income trajectories for teleworkers are not different by gender, at least in the short run. What is different is the one for non-teleworkers – female non-teleworkers experience less income loss in the short run after the lockdown. This, again, matters in interpreting the result because this result could suggest that the impact of WFH may be the same by gender, while the impact of the lockdown and/or the pandemic on non-teleworkers may be different by gender. For example, male non-teleworkers might have to reduce their hours worked more than women due to some characteristics of jobs. If this is true, then the argument might be “wages for non-teleworkers do not decline for women but do for men,” which is unrelated to the effectiveness of WFH. The current analysis cannot rule out this possibility.*

**Response:**

Figure 2 is now located in the **Panel B of Figure 2.** There has been a slight change in the income trajectory of WFH workers in wave 6. The change is driven due to the change in the sample. Specifically, our final sample changed from what we used in the original submission due to **our dropping** **all observations of respondents** if they (1) did not work in waves 1-2 regardless, and (1) worked in waves 1-2 but did not work at all for the entirety of 2020 (waves 3-6). At the time of the initial submission, we made the error of only dropping their person-wave observations with missing information. We decided to remove these individuals (all their observations in 6 waves) entirely from the sample, as we wanted to set a necessary sample eligibility rule to be that they have records of income/wage before- and after- the start of the COVID-19/Lockdown. Please refer to **Section 3.1 Data** for more details in sample selection.

In the updated Figure 2, Panel B, we see some differences between the male WFH and female WFH workers. For instance, the male WFH workers experience a sudden increase in income at the start of the Lockdown (March 2020), and a soft dip in May. But their upward trend of income continues into June and November 2020. In contrast, the income of female WFH workers do not increase at the start of the Lockdown (March), and starts rising slightly in June 2020. But the increase is not sustained in November 2020. In short, the income gain seems to benefit male WFH workers more than their female counterparts. In the updated figure, the income trajectories of non-WFH males and non-WFH females seem similar—though the non-WFH male respondents’ income seems to upshoot more rapidly in November 2020 than that of the non-WFH women. Of course, the graphical assessments in **Figure 2** should be taken with a grain of salt, as the WFH vs non-WFH comparisons by gender are done without accounting for covariates, individual/time/occupation fixed-effects, and occupation-specific time trends. We add these discussions in **Section 5.1.**

Per your advice, we have examined the effect of remote work arrangements and lockdown not only on monthly income but monthly work hours and hourly wages. We then interpreted the findings from our diff-in-diff estimation (**Table 4**) more carefully—focusing on the points of divergence between male WFH vs. female WFH workers as well as between male non-WFH vs. female non-WFH workers in **Section 5.2** and **in Section 6: Discussion.** Instead of focusing only on the coefficient of the interaction term (remote work x time), we also provided interpretations of the stand-alone coefficient of the ‘remote work’ identifier (effects on WFH individuals pre-lockdown) and of the ‘lockdown/post-lockdown’ identifiers (effects on non-WFH individuals during lockdown/post-lockdown). We have also added interpretations on the magnitude and the direction of the effects of lockdown on WFH workers (coefficient of lockdown + coefficient of the interaction term), and compared them with those of non-WFH workers (coefficient of lockdown).

Responding to the scenario you stated (“*For example, male non-teleworkers might have to reduce their hours worked more than women due to some characteristics of jobs. If this is true, then the argument might be “wages for non-teleworkers do not decline for women but do for men,” which is unrelated to the effectiveness of WFH.*”): Based on the updated results in **Table 4**, it is indeed true that male non-WFH workers reduced work hours in lockdown/post-lockdown by 4.11 hrs/month while female non-WFH workers’ hours increased by 12.85 in the same periods. Yet, wage increased significantly for both non-WFH males and females ($9.35/hr in lockdown/post-lockdown for men; $6.42/hr in lockdown/post-lockdown for women)—thereby rejecting the scenario you proposed. Overall, the effect of WFH arrangements differs across gender—especially in terms of monthly income and monthly hours worked. In contrast, the effect of non-WFH arrangements stay more consistent across gender, except on work hours. We included these discussions in **Section 5.2** and **Section 6**.

*R1C4. Still, it might be possible to state that the notion of the “effect of WFH” includes the above-mentioned possibility. Even in this case, this possibility should be mentioned, and how the readers can understand the effect of WFH in this scenario should be given so that they can understand the policy implications in a more coherent way.*

**Response:**

The possibility you have mentioned has been ruled out in our revised estimations. Please see our interpretation of the new results and the rationale for rejecting the possibility in our response to Q3 above. They are included in **Section 5.1** and **Section 6** of the manuscript as well.

*R1C5. In the analysis in section 5.3, the potential mediating roles of household responsibilities are discussed. What is the definition of the time use variable in this analysis? Namely, is this time spent on childcare at time t, or the pre-lockdown level? This is an important difference because if the former is true, then the effect seems mechanical because increasing time spent on childcare almost mechanically reduces the hours worked, which in turn decreases income. The reason why the result is insignificant for men then may be because men don’t spend time on housework. I would suggest that the authors may want to check the effects of WFH on time spent on childcare and housework in separate regressions with the same specification, which would make these stories clear. Again, the point is distinguishing between “productivity decline” and “reduced hours worked” mentioned above.*

**Response:**

As you rightfully pointed out, the traditional understanding—or the ‘mechanical’ nature—of time allocation between work and home production (i.e., childcare/chores) is that the two activities are substitutes. Yet, the theory was formulated for the traditional mode of work—done outside of home. When the work is done from home, when the boundaries blur between the work-space and the home-space, the strict substitutability of the two activities may also break down. For instance, it may be possible to do a bit of childcare while working—e.g., an analyst father may be able to run his codes on the home laptop while he plays with his child. The two activities, albeit not complements, can take place simultaneously. On a related note, with the rise of remote work, the total time allocated to work and home production could have increased—as individuals could save time that used to be spent on commuting and interacting with colleagues in the office. Then, an increased time spent on childcare would not necessarily reduce time spent on WFH if this parent were to allocate the newly-saved commuting time on childcare.

**Exploring the links between WFH and Childcare/Chores:**

To empirically test the link—or the substitutability—between WFH and household responsibilities, per your advice, we have also inspected the effects of WFH on time spent on **childcare** at time *t*, using the same covariate and fixed-effects specifications.

The results are displayed in **S9-1** **Table** below. From columns (1)-(3), we observe that the WFH is positively associated with the childcare time. The more of the work is done remotely from home, the more childcare time one spends—and this was especially true for women. During the lockdown and in the post-lockdown period, shown in columns (4)-(6), we observe that the WFH is positively associated for men who adopted full-remote work arrangements, while is negatively (insignificant) associated with women who partially or fully adopted WFH. One possible interpretation may be that the two activities are complements for fathers while largely uncorrelated for mothers (if anything, negative). The blurred boundary between home and work-space—facilitated by the adoption of WFH—took a toll on childcare time for mothers. For fathers, working from home incentivized them to do take on more childcare duties. **Regardless of the interpretations, the divergent relationship between WFH and childcare load across gender is noteworthy and is suggestive of the non-mechanical linkage between the two activities.** A traditional view that WFH and household production are substitutes would be validated if the estimated coefficients of the WFH in **S9-1 Table** are negative.

| **S9-1 Table. Effect of Remote Work Arrangements (Categorical) on Time Spent on Childcare** | | | | | | | | |
| --- | --- | --- | --- | --- | --- | --- | --- | --- |
|  | (1) | (2) | (3) | (4) | (5) | (6) | |  |
|  | Y=Childcare (min/hr) | | | | | | | |
|  | All | Male | Female | All | Male | Female | |  |
| *Work Arrangements(Reference = Work Fully Outside)* | | | |  |  |  | |  |
| Mostly outside | 0.64 | 0.23 | 1.70** | 0.69 | 0.83 | 3.58* | |  |
|  | (0.50) | (0.66) | (0.83) | (0.92) | (1.02) | (1.89) | |  |
| Half from home | 1.22** | 0.47 | 2.29** | 1.59* | 1.08 | 3.54** | |  |
|  | (0.60) | (0.63) | (1.01) | (0.96) | (1.09) | (1.53) | |  |
| Mostly from home | 2.93*** | 1.26* | 4.57*** | 2.60** | -0.43 | 6.62** | |  |
|  | (0.63) | (0.67) | (1.01) | (1.20) | (1.36) | (3.35) | |  |
| Working remotely (Fully from home) | 2.64*** | 1.45** | 4.03*** | 1.29 | 0.57 | 3.59*** | |  |
|  | (0.59) | (0.59) | (0.97) | (0.90) | (0.88) | (1.37) | |  |
| *Lockdown Policy (Reference = Pre-Lockdown)* | | | |  |  |  | |  |
| Lockdown | - | - | - | -0.33 | 0.08 | 0.04 | |  |
|  | - | - | - | (0.65) | (0.62) | (1.15) | |  |
| Post-lockdown | - | - | - | -0.16 | 0.48 | -0.20 | |  |
|  | - | - | - | (0.79) | (0.96) | (1.11) | |  |
| (*Reference = Pre-Lockdown*, *Working Fully Outside)* | | | |  |  |  | |  |
| **Mostly outside x Lockdown** | - | - | - | 0.27 | -0.64 | -1.54 | |  |
|  | - | - | - | (0.90) | (0.89) | (1.99) | |  |
| **Half from home x Lockdown** | - | - | - | 0.07 | -0.75 | -0.73 | |  |
|  | - | - | - | (0.96) | (0.98) | (1.44) | |  |
| **Mostly from home x Lockdown** | - | - | - | 0.93 | 2.09 | -1.55 | |  |
|  | - | - | - | (1.19) | (1.44) | (3.38) | |  |
| **Working remotely (Fully from home) x Lockdown** | - | - | - | 1.69** | 1.37* | -0.15 | |  |
|  | - | - | - | (0.81) | (0.79) | (1.23) | |  |
|  |  |  |  |  |  |  | |  |
| **Mostly outside x Post-lockdown** | - | - | - | -0.27 | -0.78 | -2.93 | |  |
|  | - | - | - | (1.26) | (1.30) | (2.56) | |  |
| **Half from home x Post-lockdown** | - | - | - | -1.04 | -0.83 | -2.63* | |  |
|  | - | - | - | (0.99) | (1.18) | (1.49) | |  |
| **Mostly from home x Post-lockdown** | - | - | - | -0.89 | 2.03 | -4.92 | |  |
|  | - | - | - | (1.33) | (1.66) | (4.12) | |  |
| **Working remotely (Fully from home) x Post-lockdown** | - | - | - | 0.05 | 2.14* | -2.97* | |  |
|  | - | - | - | (1.26) | (1.23) | (1.73) | |  |
|  |  |  |  |  |  |  | |  |
| Individual FE, Occupation FE, Time FE | Yes | Yes | Yes | Yes | Yes | Yes | |  |
| Occupation FE x Time FE | Yes | Yes | Yes | Yes | Yes | Yes | |  |
| Control variables | Yes | Yes | Yes | Yes | Yes | Yes | |  |
| N | 4308 | 2301 | 2007 | 4308 | 2301 | 2007 | |  |
| *p<0.1 **p<0.05 ***p<0.01 | | | | | | |  | |

Next, we estimated the effect of WFH on time spent on **chores** at time t, as shown in **S9-2 Table** below. While the WFH is once again positively associated with chore time in columns (1)-(3), the link between WFH and chore time is noisier. Specifically, for mothers (column 3), both respondents who work mostly outside or mostly from home (i.e., those conducting work partially from home, albeit at varying rates) incur greater chore times compared to those who work fully from home. Yet, this link holds true only for mothers and not fathers. There seems to be no systematic association between the fathers’ remote work adoption and chore time. During the lockdown period as shown in columns (4)-(6)), we observe that WFH is strictly and positively related to chore time—albeit this pattern is only significant among the male WFH workers and not females. This suggests that the extent of their engagements with household responsibilities increased during the lockdown compared to the previous years, at least for men. In the post-lockdown period, the systematic link between WFH and chore time subsides for both genders. **Same as before, the results on S9-2 Table seem to indicate that WFH and chores are not perfect substitutes—thereby weakening the traditionally-believed mechanical link between the two activities**.

| **S9-2 Table. Effect of Remote Work Arrangements (Categorical) on Time Spent on Chores** | | | | | | |
| --- | --- | --- | --- | --- | --- | --- |
|  | (1) | (2) | (3) | (4) | (5) | (6) |
|  | Y=Chores (min/hr) | | | | | |
|  | All | Male | Female | All | Male | Female |
| *Work Arrangements(Reference = Work Fully Outside)* | | | | | | |
| Mostly outside | 0.34 | -0.26 | 1.07* | 0.21 | -0.41 | 0.73 |
|  | (0.34) | (0.34) | (0.65) | (0.72) | (0.68) | (1.56) |
| Half from home | -0.02 | -0.32 | 0.26 | -0.72 | -0.88 | -0.32 |
|  | (0.30) | (0.37) | (0.44) | (0.46) | (0.55) | (0.71) |
| Mostly from home | 0.68* | 0.13 | 1.34** | -0.23 | -0.57 | 0.45 |
|  | (0.38) | (0.38) | (0.63) | (0.49) | (0.64) | (0.85) |
| Working remotely (Fully from home) | 0.26 | -0.15 | 0.81 | -0.70* | -0.68 | -0.17 |
|  | (0.31) | (0.36) | (0.51) | (0.39) | (0.44) | (0.67) |
| *Lockdown Policy (Reference = Pre-Lockdown)* |  |  |  |  |  |  |
| Lockdown | - | - | - | -0.19 | -0.43 | 0.15 |
|  | - | - | - | (0.31) | (0.32) | (0.66) |
| Post-lockdown | - | - | - | -0.01 | -0.26 | 0.24 |
|  | - | - | - | (0.36) | (0.39) | (0.57) |
| (*Reference = Pre-Lockdown*, *Working Fully Outside)* |  |  |  |  |  |  |
| **Mostly outside x Lockdown** | - | - | - | 0.45 | 0.30 | 0.90 |
|  | - | - | - | (0.68) | (0.56) | (1.45) |
| **Half from home x Lockdown** | - | - | - | 0.96** | 0.49 | 1.01 |
|  | - | - | - | (0.46) | (0.49) | (0.78) |
| **Mostly from home x Lockdown** | - | - | - | 1.59** | 0.99 | 1.92* |
|  | - | - | - | (0.63) | (0.66) | (1.14) |
| **Working remotely (Fully from home) x Lockdown** | - | - | - | 1.38*** | 0.86** | 1.17 |
|  | - | - | - | (0.41) | (0.38) | (0.76) |
|  |  |  |  |  |  |  |
| **Mostly outside x Post-lockdown** | - | - | - | -0.08 | 0.01 | -0.19 |
|  | - | - | - | (0.82) | (0.87) | (1.68) |
| **Half from home x Post-lockdown** | - | - | - | 0.75 | 0.85 | 0.37 |
|  | - | - | - | (0.58) | (0.54) | (0.88) |
| **Mostly from home x Post-lockdown** | - | - | - | 0.37 | 0.75 | -0.29 |
|  | - | - | - | (0.54) | (0.65) | (0.87) |
| **Working remotely (Fully from home) x Post-lockdown** | - | - | - | 0.42 | 0.70 | -0.22 |
|  | - | - | - | (0.47) | (0.46) | (0.72) |
|  |  |  |  |  |  |  |
| Individual FE, Occupation FE, Time FE | Yes | Yes | Yes | Yes | Yes | Yes |
| Occupation FE x Time FE | Yes | Yes | Yes | Yes | Yes | Yes |
| Control variables | Yes | Yes | Yes | Yes | Yes | Yes |
| N | 4308 | 2301 | 2007 | 4308 | 2301 | 2007 |
| *p<0.1 **p<0.05 ***p<0.01 | | | | | |  |

Given the complex and multidirectional link between WFH and childcare/chore time, we have decided to **keep our measures of childcare/chore at time t in Table 6** as in the original submission**.** In addition, **per your advice, we have changed the childcare/chore to be the average of waves 1-2 and re-run the estimations**. The results are shown in **Table 6 (ver 2)** below. The results are consistent with that of Table 6 in the manuscript. We now have added these discussions to **Section 6,** as well as in the **Supporting Information** under **S9-1 Table** and **S9-2 Table**.

| **Table 6 (ver 2). Mediating Role of Household Work on Hourly Wage, Monthly Income, and Monthly Hours Worked** | | | | | | | | | |
| --- | --- | --- | --- | --- | --- | --- | --- | --- | --- |
|  | (1) | (2) | (3) | (4) | (5) | (6) | (7) | (8) | (9) |
|  | Y=Hourly Wage | | | Y=Monthly Income | | | Y=Monthly Hours Worked | | |
|  | All | Male | Female | All | Male | Female | All | Male | Female |
| *Panel A: Role of Childcare (minutes/hr)* ҂ | | |  |  |  |  |  |  |  |
| **Remote x Lockdown x Childcare** | 0.13 | -0.53 | 0.33 | -15.34 | 31.31 | -17.98 | 0.32 | 0.62 | 0.52 |
|  | (1.37) | (0.79) | (1.65) | (14.84) | (31.72) | (16.53) | (0.45) | (1.16) | (0.48) |
| **Remote x Post-Lockdown x Childcare** | 0.06 | 0.34 | 0.16 | -21.56** | -47.05 | -9.07 | 1.19 | -1.16 | 2.37** |
|  | (0.91) | (1.51) | (1.18) | (10.94) | (33.31) | (12.06) | (0.81) | (1.76) | (1.09) |
| *Panel B: Role of Chores (minutes/hr) ҂* | | |  |  |  |  |  |  |  |
| **Remote x Lockdown x Chores** | -0.80 | 0.46 | -2.45** | -67.95 | 48.53 | -93.39** | 0.54 | 0.57 | 3.06 |
|  | (0.92) | (1.98) | (1.01) | (52.47) | (108.76) | (44.75) | (1.74) | (2.42) | (2.17) |
| **Remote x Post-Lockdown x Chores** | 1.28 | 12.36 | -5.62** | -195.82** | -105.67 | -191.83** | -10.35 | -10.10* | -6.79 |
|  | (4.23) | (7.63) | (2.41) | (75.90) | (112.37) | (76.44) | (8.47) | (5.57) | (13.00) |
| *Panel C: Role of Whether Respondents Receive Any Help for Household Work (1 yes 0 no)* | | | | | | | |  |  |
| **Remote x Lockdown x Receiving any help** | 0.01 | 0.01 | 0.01 | 0.01 | 0.01 | 0.01 | 0.01 | 0.01 | 0.01 |
|  | (0.00) | (0.00) | (0.00) | (0.00) | (0.00) | (0.00) | (0.00) | (0.00) | (0.00) |
| **Remote x Post-Lockdown x Receiving any help** | 2.05 | -13.57 | 15.06 | 581.18* | 574.47 | 644.64* | 30.12 | 18.04 | 37.85 |
|  | (10.63) | (19.90) | (9.72) | (308.33) | (476.32) | (379.75) | (23.81) | (23.61) | (40.53) |
| *Panel D: Role of Whether Respondents have Remotely-Working Spouses (1 yes 0 no)* | | | | | | | |  |  |
| **Remote x Lockdown x Spouse working remotely** | 0.01 | 0.01 | 0.01 | 0.01 | 0.01 | 0.01 | 0.01 | 0.01 | 0.01 |
|  | (0.00) | (0.00) | (0.00) | (0.00) | (0.00) | (0.00) | (0.00) | (0.00) | (0.00) |
| **Remote x Post-Lockdown x Spouse working remotely** | 22.66* | 29.35 | 11.55 | 579.22* | 990.47** | -18.33 | 19.18 | 8.77 | 9.29 |
|  | (12.49) | (18.36) | (12.36) | (308.11) | (497.07) | (318.97) | (29.69) | (27.62) | (35.49) |
|  |  |  |  |  |  |  |  |  |  |
| Individual, Occupation, Time FEs | Yes | Yes | Yes | Yes | Yes | Yes | Yes | Yes | Yes |
| Occupation FE x Time FE | Yes | Yes | Yes | Yes | Yes | Yes | Yes | Yes | Yes |
| Control variables | Yes | Yes | Yes | Yes | Yes | Yes | Yes | Yes | Yes |
| N | 4308 | 2301 | 2007 | 4308 | 2301 | 2007 | 4308 | 2301 | 2007 |
| ҂ For each respondent, the time spent in childcare is calculated as the average of pre-Lockdown waves 1-2. The chore time is calculated in the same manner. | | | | | | | | | |
| Notes: Panels A-D are run separately because the indicators of household responsibilities are highly collinear. Each component of the interaction terms is also included in the regressions, but are not shown in the table. The reference time period is ‘Pre-lockdown’, between April-July 2018 and December 2019, prior to the COVID-19 pandemic. ‘Lockdown’ refers to March and June 2020, while ‘Post-lockdown’ refers to November 2020, six months after the end of the lockdown. Standard errors, shown in the parentheses, are clustered at the household level. *p<0.1 **p<0.05 ***p<0.01 | | | | | | | | | |

**Reviewer #2**

*Comments to the Author
In this paper the authors conducted an ad hoc survey targeting married women in Singapore, for whom they collect longitudinal information at 6 points in time (from 2018 to November 2020) on both them and their male partners. The aim is to analyze the effect of the COVID-19 pandemic and the subsequent period on monthly wages and to see if there have been differences in the effects of the pandemic between teleworkers and non-teleworkers across genders. The authors found a significant salary income growth among remotely-working male spouses, but not among female spouses.*

*The paper addresses a relevant topic related to the effects of the COVID-19 pandemic on wages and gender inequality. It is an interesting paper, and it is, overall, well written.*

*I have several comments related to the organisation of the paper and the need for clarification of some aspects. The motivation in the introduction should also be improved. There are aspects that in my opinion are key to properly motivate the article (such as telework figures in Singapore, the effects of telework on productivity, etc. ....) that should be explained in the Introduction (see my specific comments below).*

*Despite having a general favourable opinion of the paper, my main concern lies with the estimation results.* ***Firstly****, the authors analyse monthly salaries, but they do not control for the number of working hours despite having information on this issue.* ***Secondly****, it is no clear if the analysed individuals are employed consistently across all periods. For me, it would be surprising that all individuals and their partners were consistently employed throughout.* ***Third****, the definition of teleworkers is very rigid (only those workers who fully work from home), which implies that their main explanatory variable (working from home) is almost zero before the pandemic.* ***Lastly****, there are some inconsistencies between the coefficients obtained in the estimations for the total sample (pooled) and the separate estimations for men and women (see for example the coefficient of the variable "lockdown" in the three samples in Table 4). The coefficient is negative for the pooled sample (implying that during the lockdown the monthly wages of non-teleworkers decreased). However, when separate estimates are made for men and women, the coefficient is positive for both, indicating just the opposite. There can also be observed several inconsistencies for other coefficients. For these reasons, I believe that the authors should carefully review the regression results. I therefore consider that the article requires major revisions.*

**Response:**

Your comments have been incredibly helpful in improving the paper. We have endeavored to address all your comments, and will be happy to address any remaining comments or suggestions that you may have.

Regarding your **first** comment, please see our response to **R2C17**. We have now re-run all estimations to show the impact of remote work and lockdown on monthly income, monthly hours worked, and hourly wages.

RE your **second** comment: Please see our response to **R2C13**. Based on our sample eligibility criteria (*not* the survey criteria, we have edited the manuscript to make this part clearer), we intentionally kept only the individuals employed throughout our analytic periods to measure the effect of adopting remote work arrangements *conditioning on individuals’ continued employment.*

RE the **third** comment: Regarding the rigidity of the WFH identifier, please see our response to **R2C19.** We intentionally used a rigid definition of remote work. Because the jobs that had to be done outside of home (either partially or fully) would have experienced disruptions due to COVID-19 and the government-mandated Lockdown, we wanted to distinguish this group from those who could do all their work from home (i.e., full WFH workers). **While we kept the rigid definition of WFH in the revision,** we wholeheartedly acknowledge the value of your comment and have run the main DID estimations with a broader definition of remote work that you suggested. The results are displayed in **S8** **Table** in the **Supporting Information** in our response to **R2C19** below.

RE the **fourth** comment: As for the inconsistencies in the estimated coefficients, please see our detailed response below—e.g., our response to **R2C27**. They are either typos or driven by our past erroneous sample selection in the initial submission.

*MAIN COMMENTS (ORGANIZED FOLLOWING THE MANUSCRIPT STRUCTURE):*

*INTRODUCTION*

*R2C1. In the introduction I miss some figures (based on official statistics) on telework in Singapore before and after the pandemic, differentiating by gender.*

**Response:**

According to Ministry of Manpower (2021a), Singapore had one of the highest shares of employed persons working remotely in 2020—with 1,094,900, or approximately 49% out of total employed residents working from home (WFH). This rate was similar to the ones observed in Netherlands (47.4%), Denmark (53.0%), U.S. (50%) (Ministry of Manpower 2021a; Dalton and Groen, 2022).

**Across gender, 43.6% of men and 55.9% of women worked remotely in 2020.** In terms of occupation, managers (74.3%), professionals (74.5%), associate professionals and technicians (58.1%), and clerical support workers (51.9%) had the highest percentage of WFH employees. Industries with highest WFH operations included information & communication (77.6%), financial & insurance services (76.3%), and professional services (74.7%), while the administrative & support services (29.4%) and health & social services (28.1%)) accommodated lowest rates of WFH. While no official statistics track the extent of remote work done prior to 2020, the 2020 Conditions of Employment Survey (Ministry of Manpower, 2021b) reports on the proportion of establishments offering formal ‘flexible work arrangements’ that states that the share of companies with the arrangements grew from 46.8% in 2014 to 78.5% in 2020. Of note, the flexible work arrangements include not only the remote work arrangements but part-time work, flexible hours/staggered hours, job sharing, and compressed work-weeks.

Unfortunately, neither the trajectory of provision of flexible work arrangements nor the share of remote work conducted in each occupation or industry can be further broken down by gender as such statistics are not available. We have now added some of these gender-specific descriptions to the **Introduction** of the manuscript.

*R2C2. Although explained on page 14 of the manuscript, it would be useful to mention also in the introduction that the authors exploit a non-official ad-hoc survey approved by the National University of Singapore Institutional Review Board. Additionally, the purpose of the survey should be stated.*

**Response:**

We collected primary data—a first-hand survey—that was approved by the NUS IRB (Ref #: LN-17-048). The first wave of the survey was conducted in 2018 for the initial purpose of collecting a nationally representative data on married women in Singapore. Given the field of expertise of Dr. Poh Lin TAN who is the Principal Investigator of the survey, the focus of the survey was to collect information on the female respondents and their spouses on their family composition and characteristics (e.g., household size, number of children, birth of the children, living arrangements), marriage (e.g., marital satisfaction, division of chores/childcare/ home responsibilities, marital attitudes, household attitudes), fertility (e.g., pregnancy-related questions, decision to have children, access to childcare, expectation vs reality), and health (e.g., general health, sexual habits, menstrual cycles, fertility habits, lifestyle habits)—in addition to the basic demographic characteristics, work/employment statuses, and housing questions.

At the time of the COVID-19 outbreak, we added COVID-19 related and additional work/productivity related questions in the subsequent survey waves disseminated in 2020. Our goal was to leverage the panel nature of the survey and retrospective questions (i.e., questions that ask about past waves) to establish 6 waves from 2018 to Nov 2020 to analyze the effect of the COVID-19 and the varying extents of adoption of remote work arrangements on the labor market outcomes (i.e., salary income, hourly wages) of working parents in Singapore. We now have added the purpose of the survey in the manuscript (**Introduction**).

*R2C3. In the introduction, it should be specified that the salary income is measured on a monthly basis. I guess that the observed increase 'in monthly salary' reported by the authors is related to an increase in the number of working hours (that authors do not explain nor mention).*

**Response:**

We have now edited the **Introduction** so that we specify that the income refers to ‘monthly salary income’. To inspect whether the increased income stems from increased work hours, we have now re-run all estimations using three outcome variables: hourly wage, monthly income, and monthly hours worked. In fact, the increased monthly income seems to stem from the increased productivity (increased hourly wage) of individuals who adopted full remote work arrangements (i.e., See Table 4) and not from the increased work hours. Please see the tables and **Section 5** for updates and additional interpretations.

*R2C4. On page 4, last paragraph, the authors state, as an additional contribution of their article, the fact that “The current literature on the labor market impact of Covid19 primarily relies on evidence from Western or larger economies. Such evidence may not apply to non-Western smaller economies given the international heterogeneity in the institutional responses to Covid19, extents of adoption of remote work, and sociocultural contexts”. I suggest adding a short sentence highlighting the main differences between Western and non-Western countries in the fight against COVID-19.*

**Response:**

Per your advice, we have augmented the descriptions on the key East-West differences in COVID-19 interventions in the manuscript (**Introduction**) adding the details below:

The extant literature suggests a significant East-West divide in the fight against COVID-19. While many Western economies (e.g., Italy, UK, US) suffered from slow rollouts of COVID-19 interventions due to politically-driven and -managed government regulations and healthcare delivery systems, Asian countries (e.g., Singapore, South Korea, Taiwan, Vietnam, Hong Kong SAR) were quicker to centralize disease control and vaccine rollouts by bypassing bureaucratic structures of healthcare administration (Jamison and Wu, 2021; Liu et al., 2023; Shokoohi et al., 2020). Institutional differences played a part in the speed and the adoption of the COVID-19 intervention measures (Mellish et al., 2020), with the Western regimes built on the neoliberal policies of deregulation and privatization while many Eastern regimes are still relatively more centralized and regulates. Another difference lied in the West’s hospital-centered public health strategies versus the East’s community-centered strategies in rapid testing, treatment, and care rollouts (Navarro, 2021; Shokoohi et al., 2020).

Such East-West differences in the speed, scale, and channels of COVID-19 interventions, as well as the varying rates of public’s adoption of prevention/treatment tools, would have affected lay people’s labor force participation and production activities.

*R2C5. Figure 1 is mentioned on page 5, last paragraph, and the authors say that by the fourth quarter of 2020 the economy had almost bounced back to pre-pandemic activity levels (“By the last quarter of 2020, the economy had almost bounced back to pre-pandemic activity levels, which coincided with a steep decline in number of daily confirmed cases to mostly single digits (see Figure 1)”. However, looking at Figure 1, it appears that the growth rate of GDP in the fourth quarter of 2020 is negative (could it be a visual effect on the horizontal axis?). I assume that the growth rates shown in Figure 1 are year-on-year growth rates, but it would be useful to make this explicit in the figure. In addition, it would be useful to add employment growth, differentiated by gender.*

**Response:**

We wrote that the economy started to bounce back because the rate of deceleration of GDP slowed down significantly at this time. But, as you correctly pointed out, the *quarterly* real GDP growth in Q4 2020 was indeed negative, but only slightly: -0.5. The GDP growths became positive in 2021 Q1 and onward. We have edited our descriptions in the **Introduction** under **Figure 1**. We also listed that the GDPs show quarterly growths. Lastly, we added quarterly employment growths for the whole population (Panel B of Figure 1). The statistics were not available by gender.

*SECTION 2 – BACKGROUND*

*R2C6. I find it a bit confusing to mix the background section, which is usually a literature review, with aspects of the specific contribution of the article as well as the hypothesis statement of the paper. I suggest revising this section so that it is more standard with what is usually done in academic articles. A simple option would be to change the title of Section 2. For me, it would be more natural (it is a personal taste) to focus the background section on literature review and specify the hypotheses in the Estimation section, before the equation 1, to better link how the three hypotheses of the article will be tested through equation 1.*

**Response:**

We have streamlined the three sub-sections of the Background into two that focuses on reviewing the literature: 2.1 Remote work and labor market outcomes and 2.2 gender considerations of remote work, and included Singapore’s context-specific information (formerly our third sub-section) into the two sections. We have pulled out the three hypotheses into a new subsection—2.3. Hypothesis. Please see **Section 2** and its **subsections 2.1-2.3** in the manuscript for the changes. We considered the possibility of adding the hypotheses to the Estimation Section but decided against it, as the hypotheses are built on the insights from the literature and contextual specificity of Singapore detailed in **Section 2.**

*R2C7. On page 8, the authors say “In case of Singapore, based on the qualitative evidence that the share of respondents who reported productivity increases following the introduction of remote work rose from 15.1% in April 2020 to 22.5% in June 2020, and the share of respondents who were concerned about productivity declines fell from 78% to 40%”. In my opinion this should be in the Introduction to support the motivation of the article. Also, in relation to Hypothesis 1, the authors should explain the mechanism by which they assume that teleworking implies an improvement in wages. This should be explained more clearly.*

**Response:**

Per your advice, we have moved the information to the **Introduction** (first full paragraph).

As for the Hypothesis 1 (See **Section** **2.3 Hypothesis**), we have now added the mechanism by which remotely-working individuals incurred income gains: “Compared to individuals who worked outside, individuals who were able to shift to remote work during the lockdown period experienced income gains due to *increased productivity—facilitated potentially by added flexibility, reduced disruption from peers, and reduced (psychological) burden of commuting.* The gains persisted over time.” Based on the insights from the literature from the Western contexts (Baert et al., 2020; Bosua et al., 2013; EngageRocket, 2020; Kazekami, 2020), we suspect that the changes in salary income is driven primarily by changes in productivity—which are measured in terms of hourly wages in our revised estimations.

*R2C8. On page 9, first paragraph, the labour market in Singapore is partially discussed. Personally, I do not know the characteristics of the labour market in Singapore. I think that for the international reader, especially those from western countries, it would be very useful to provide some figures on the labour market from a gender perspective in Singapore. What is the employment rate of men and women? How is employment distributed between full-time and part-time between men and women? It would also be useful to show a table, even if* ***only in the appendix,*** *with the distribution of employment by occupations and industries differentiated by gender, in order to see which occupations and industries are more feminised in the case of Singapore.*

**Response:**

Per your guidance, we now have added a detailed description of the characteristics of the labor market in Singapore. According to *2020 Labour Force in Singapore* (Ministry of Manpower, 2021a), out of the total population of 1,152,800 of male Singaporean residents aged 25-64 in 2020, 92.0% were in the labor force. Among the total of 1,218,800 female Singaporean residents within the same age range, 76.6% were in the labor force. Leaving aside the unemployed persons (i.e., active job-seekers), employment rate for Singaporean residents aged 25-64 in 2020 was 87.9% out of a total for men and 73.2% for women. Among the employed male residents, approximately 92.5% worked full-time and 7.5% worked part time. For female residents, about 85.8% worked full-time and 14.2% in part-time positions. Overall, the Singapore’s labor market in 2020 portrayed clear gender differences—with men exhibiting greater labor force participations, larger employment rates, and better job security proxied by their attachment to full-time positions.

As for the occupation-specific distributions, we observe that out of the total of 1,198,100 employed male Singaporeans *across all ages* in 2020,^[1]^ 60.4% were in high-income, white-collar occupations (managers/ administrators/ professionals/ assoc. professionals/ technicians); 13.3% in the remaining white-collar occupations (clerical support, service & sales); 4.7% in skilled manual positions (craftsman and trade workers); and 21.8% in semi-skilled or low-skilled manual jobs (plant and machine operators/assemblers/cleaners/laborers/other related workers). Among the total of 1,024,600 employed females *across all ages* in the same year,^[2]^ 59.3% were in high-income, white-collar occupations; 30.1% in the remaining white-collar occupations; 0.8% in skilled manual positions; and 9.8% in semi-skilled or low-skilled manual jobs. While the heavy feminization of the clerical and services & sales positions was consistent with the occupational distribution in other countries, Singapore’s workforce tended to be predominantly white-collar—with high shares for both genders compared to their counterparts in other developed nations.

Regarding the industry-specific distributions, out of the total of 1,198,100 males across all ages in 2020, 11.3% worked in the manufacturing industry; 5.8%, construction; 81.7%, services; and 1.3%, others. Among the total of 1,024,600 females, 7.6% operated in manufacturing sector; 2.8%, construction; 89.1%, services; and 0.6%, others. While Singapore had more male workers in the manufacturing and construction sectors, the services sector encompassed more females. We have added some of these descriptions regarding the Singapore’s labor market—focusing on its unique features— in **Introduction**. We also added a fuller description of the labor market in Singapore in the **Supporting Information**, along with two tables detailing the evolution of the occupation- and industry- distributions across gender between 2010-2020 in the **S1 Table** and **S2 Table** in the **Supporting Information.** Thank you.

*R2C9. As the authors partially mention, women were more likely to leave employment or reduce hours worked than men. This is a phenomenon that has been noted especially for the literature based on western countries. Did this happen in Singapore as well ? Can the authors provide some figures to contextualise this phenomenon for Singapore?*

**Response:**

In case of Singapore, the employment rate did not drop considerably between 2019 and 2020 due to nation-wide COVID-19 support measures such as Jobs Support Schemes (JSS) for wage support, SGUnited Jobs and Skills Package and Jobs Growth Incentives that promoted hiring and retaining of locals during the pandemic (Chew, 2022). More specifically, the employment rates of men aged 25-64 drops very slightly from 88.8% to 87.9% between 2019-2020, and that of women drops from 73.3% to 73.2%. Between 2020 and 2021, both rates increased to be even slightly higher than that of 2019 (Ministry of Manpower, 2022).

Yet, we see divergences across genders in terms of work hours. The official statistics show that female Singaporean employed residents (9.4%) experienced greater reduction in working hours or more frequent implementation of temporary no-pay leaves than men (8.8%) in 2020 (Ministry of Manpower, 2021a). We have now added this information in **Section 2.2.**

*R2C10. A personal taste: I find the transition to section 3 a bit rough. Perhaps it would be appropriate to end with a few brief lines to give way more naturally to the data section.*

**Response:**

Per your advice, we have added at the end of Section two the following line, “*The following section details the data used to empirically evaluate the three hypotheses, key variables, and summary statistics of the final sample*,” to allow for a smoother navigation between sections (**Section 2.3**). Thank you.

*SECTION 3 – DATA AND METHODS*

*R2C11. When explaining the database, it is necessary to specify that the survey is based on ad-hoc survey (I imagine that was conducted by the researchers in the context of a research project endorsed by the National University of Singapore). Additionally, it is necessary to specify the purpose of the survey. Given that the survey started in 2018 it was impossible to know at that time that there was going to be a pandemic in 2020. What was the original objective of the survey?*

**Response:**

As elaborated in our response to R2C2, the survey’s baseline wave (wave 1) was conducted in 2018 for the initial purpose of collecting a nationally representative data on married women in Singapore (approved by the NUS IRB Ref #: LN-17-048). Given the field of expertise of Dr. Poh Lin TAN who is the Principal Investigator of the survey, the focus of the survey was to collect information on the female respondents and their spouses on their family composition and characteristics (e.g., household size, number of children, birth of the children, living arrangements), marriage (e.g., marital satisfaction, division of chores/childcare/ home responsibilities, marital attitudes, household attitudes), fertility (e.g., pregnancy-related questions, decision to have children, access to childcare, expectation vs reality), and health (e.g., general health, sexual habits, menstrual cycles, fertility habits, lifestyle habits)—in addition to the basic demographic characteristics, work/employment statuses, and housing questions.

At the time of the COVID-19 outbreak, we added COVID-19 related and additional work/productivity related questions in the subsequent survey waves disseminated in 2020. We now have added the purpose of the survey in the manuscript (**Section 3.1**)

*R2C12. It is important to describe briefly the type of information collected in the survey.*

**Response:**

The type of information collected in the survey is described in our response to Q11 above. At the onset of COVID-19 in late 2019, we added new questions on COVID-19, labor market outcomes (salary income, work hours, wages), and remote work arrangements in the subsequent online surveys disseminated in 2020—asking questions regarding respondents’ current states as well as past states (i.e., with retrospective questions asking about the past waves) to assess the effect of COVID-19. This information has been added to the **manuscript** (**Section 3.1**). Thank you!

*R2C1.) As the face-to-face surveys in 2018 were conducted on the street and only among women indicates (I imagine) that there is a proportion of female respondents who are NOT working. Were the surveys conducted during "non-working" hours? The authors state that the criteria for being surveyed are "Participants met the following inclusion criteria: currently married in 2018; either a Singaporean citizen or married to a Singaporean citizen, and able to read, write and speak in English". Therefore, being employed is not included as a criterion. Given that the survey collects wage information, were all female respondents employed in 2018? How is the distribution of the sample in terms of percentage of employment compared to what is observed in Singapore's official statistics? Were the husbands also employed?*

**Response:**

Yes, there is indeed a portion of the survey respondents who were not working at any waves, who were not working in 2018 (waves 1-2) but started working in 2020, or who were working in 2018 but stopped working in 2020. The inclusion criteria you mentioned (*"Participants met the following inclusion criteria: currently married in 2018; either a Singaporean citizen or married to a Singaporean citizen, and able to read, write and speak in English"*) was for the survey that was initially collected in 2018 for another purpose,^[3]^ and does *not* include the full sample selection criteria we used in our paper.

The original survey was collected using the above-mentioned criteria, and included 660 female and 660 male respondents between ages 24 and 59—3,960 person-wave observations for each gender in 6 waves. Among the total of 7,920 person-wave observations, 2,796 were unemployed or out of the labor force (1,269 men and 1,527 women). This suggests that 2,691 male respondents (i.e., 67.95% of the total of 3,960 male person-wave observations) and 2,433 female respondents (61.44% of the total of 3,960 females) were employed. Compared to the 88.94% employment among the male population aged 25-59 and 76.20% employment among the females reported in the Singapore’s official statistics in *2020 Labour Force in Singapore* report (Ministry of Manpower, 2021), our data undersampled employed persons.

We dropped from our final sample all person-wave observations for (1) individuals who did not work in waves 1-2, in 2018, and (2) individuals who worked in waves 1-2 but stopped working in waves 3-6 in 2020, and (3) individuals who stayed unemployed/out of the labor force for all 6 waves. This was to distinguish the effect of remote work adoption on earned income from that of job displacement/acquisition across waves. Including individuals in case (1) would have introduced an upward bias (positive bias) to the effect of remote work arrangements on income, and including case (2) would have generated a downward bias.

In short, in our final sample, **we intentionally kept only the individuals employed throughout our analytic periods to measure the effect of adopting remote work arrangements *conditioning on individuals’ continued employment.*** As our data initially undersampled employed persons, our sample selection strategy, in a way, helped bring our sample’s employment rates to be closer those reported in the official statistics. We further discuss this in our response to **R2C18** and **R2C23** below. We have now combined the descriptions on the survey collection criteria and the final sample selection criteria to avoid confusing the readers in the **manuscript** (**Section 3.1**).

*R2C1.) In Table 1, it is clear that the first interview was face-to-face, but the verbal explanation on page 12, last paragraph, should also be stated.*

**Response:**

Per your advice, we now have added the descriptions on survey collection—in the first paragraph of Section 3: Data—in **Table 1**, under notes.

*R2C15. I consider that the elaboration of an ad-hoc survey is in itself a contribution of the article and the fact that the authors conduct a survey is because the type of information collected in official surveys in Singapore is not sufficient for the purposes of the article. I do not know the characteristics of official surveys in Singapore, but at least in European countries the type of information collected by the authors is included in labour force surveys and other surveys such as living conditions surveys. Could the authors explain what kind of additional information their survey provides compared to official statistics in Singapore?*

**Response:**

At the first wave, the primary reason for collecting this dataset was to understand the impact on *family wellbeing and fertility intentions*. Such sensitive data are not generally available, certainly not from administrative sources (the ‘official statistics’). Moreover, publicly available data from the SG administrative sources do not provide panel data that tracks same individuals across time—especially at the periods leading up to and following the major points of COVID-19 interventions. Unavailability of publicly-available secondary data that contains such information incentivized us to collect a primary data consisting of multiple waves.

*R2C16. On page 13, second paragraph, the authors say “Waves 3 and 4 included retrospective questions which collected information from periods before and shortly after the lockdown”. However, looking at Table 1, I wonder if it could be a typo since Table 1 says that information in the Wave 3 is retrospective. Also, Table 1 states that the data for May 2020 is "actual", while the text states that it is retrospective.*

**Response:**

The sentence as well as Table 1 contained typographical errors. We edited **Table 1** as shown below. The surveys were collected three times between 2018 and 2020: (1) April 2018 (in-person), (2) May 2020 (online), and (3) November 2020 (online). The surveys disseminated 2020 included retrospective questions. For instance, the May 2020 survey asked about the respondents’ conditions in May as well as in Dec. 2019 and March-April 2020—slightly before and during the imposing of the lockdown. The November 2020 survey asked about conditions in November as well as in June 2020—shortly after the lockdown was lifted. Taking advantage of the temporal variations in the questions we asked, we constructed a total of 6 waves in our estimations: April 2018 (wave 1), December 2019 (wave 2), March-April 2020 (wave 3), May 2020 (wave 4), June 2020 (wave 5), and November 2020 (wave 6). Wave 2, 3, and 5 were reverse-engineered.

**Table 1. Key Dates of the COVID-19 Pandemic and Survey Data Collection**

|  | April-July 2018 | Dec 2019 | Jan 2020 | Mar-April 2020 | May 2020 | June 2020 | Nov. 2020 |
| --- | --- | --- | --- | --- | --- | --- | --- |
| Pandemic timeline | Pre-crisis |  | Singapore confirms first infection case on Jan 23 | Circuit breaker begins on 7 April | Circuit breaker cont. | Circuit breaker ends on June 1; Phases I/II commence | Phase II with relaxed restric-tions |
| Data collection timeline | In-person survey |  |  |  | Online survey |  | Online survey |
| Dates of Dissemi-nation | April 17-22, 2018 |  |  |  | May 15-20, 2020 | June 15-20, 2020 | Nov 20-25, 2020 |
| Survey waves | Wave 1 (actual) | Wave 2 (reverse-engineered) |  | Wave 3 (reverse-engineered) | Wave 4 (actual) | Wave 5 (reverse-engineered) | Wave 6 (actual) |

Notes:

1. The baseline survey in 2018 (wave 1) was conducted face-to-face in English without the presence of third parties, either in nearby public spaces, or alternatively, at the respondents’ homes at a time of their convenience. The baseline survey and the subsequent waves collected detailed information on the respondents (all female)’ and their spouses (all male)*.*
2. Due to the unexpected emergence of the pandemic, survey waves conducted in 2020 included both current and retrospective questions regarding respondents’ labor market outcomes and remote work arrangements immediately before- and shortly after- the Lockdown (Mar-April 2020). Based on all information collected across four points in time, a total of 6 waves were constructed in our data for respondents and their spouses.
3. This study, survey questionnaire, as well as all consent forms were pre-approved by the National University of Singapore Institutional Review Board (Ref #: LN-17-048). **Section 3.1 Data** contains further information.

*R2C17. Dependent variable. My main concern comes from the dependent variable because it measures the monthly wage and not the hourly wage. Given that women may have decided (voluntarily or not) to reduce their working hours due to the pandemic, it would be rational to observe a decrease in their monthly wages (because they work fewer hours). I think it would be important to make a differentiated analysis for full-time and part-time workers. I guess that the survey collects information on hours worked (Table A3 in the appendix). Information on hours worked is essential for the analysis carried out by the authors. Additionally, to contextualise the salary it would be important to provide the average salary of men and women in Singapore (according to official statistics).*

**Response:**

We agree that we need to estimate the effect of COVID-19 induced Lockdown and remote work arrangements on hourly wage (i.e., a proxy for productivity). Per your advice, we have revised the estimations so that our results show both hourly wages, monthly income, and monthly hours worked as outcomes (as indicated in **Section 3.2**). All Tables have been updated to reflect the changes in the outcome variables. Descriptions in **Sections 4-6** in the manuscript have been altered accordingly. We have now discussed the significance of the changes in salary income in our results vis-à-vis the average monthly income of men (SGD 4,719 in 2020, including employer-matched pension amount) and women (SGD 4,374 in 2020) in Singapore in **Section 6** **(p.36**): “Considering that the average monthly income was $4,719 for males and $4,374 for females in 2020 (Ministry of Labor, 2021a), the increased income generated by adopting WFH arrangements under lockdown/post-lockdown constitutes 8.2-9.5% growth for men while comprising mere 1.2-1.5% (statistically insignificant) growths for women.”

While we recognize the value of running separate analyses for full-time and part-time workers, our survey unfortunately contains no such information. We have suggested the FT vs. PT assessments as a potential topic for future research in **Section 6: Discussion**.

*R2C18. It is not clear what happens as regards employment situation across the 6 waves. Reading the paper, it seems that during the 6 waves all the people surveyed remain employed, but this would be surprising, even in times of economic expansion there are always flows in the labour market which mean that in certain periods people are not employed. How is it possible to collect monthly wages for 6 periods for married women and their husbands? Are both of them employed during all 6 periods?*

**Response:**

A part of your question can be answered by our sample eligibility criteria in the last paragraph of **Section 3.1: Data**. However, we have spotted an error in one of the criteria. We have revised it and added a fuller description, as shown below:

The original survey data had 660 female and 660 male respondents—who were present in all 6 survey ways. We dropped 68 person-wave observations who were separated or divorced, leaving 655 men and 655 women in waves 1-5, and 651 each in wave 6. We dropped 3,184 person-wave observations (all observations for 531 individuals across waves) if they did not work in waves 1-2, prior to COVID-19 and Lockdown, as changes across time in their salary earnings or wages cannot be measured. Further excluded were 360 person-wave observations (all observations for 60 individuals across waves)for having worked in waves 1-2 but not working in waves 3-6 in 2020, since the beginning of the COVID-19 pandemic. All remaining observations had reported their remote work statuses as well as monthly salary income, hours worked per month, and hourly wage (i.e., main explanatory variables) and covariates. In the end, our sample had 4,308 person-wave observations.

It is the underlined steps above that led our sample to include only those consistently married and employed across all six waves. The continuous marital status was important in that, if a respondent was divorced in a survey wave, she would have no response to provide for the husband. Because we explored the role of household work division across spouses as mediators in this study, we required the spouse information. As for the employment: We dropped from our sample individuals who were not working in waves 1-2 and started working in 2020, as well as those who were working in waves 1-2 but stopped in 2020s to **distinguish the effects of adopting remote work arrangements on earned income from the effects of job attainment/displacement.** Our sample still accommodated switching between jobs over time. Please refer to our response to **R2C23** below for a full description on the sample selection. We have now included these descriptions in the **manuscript** (**Section 3.1**). Thank you.

*R2C19. Classification of teleworkers. The authors define a teleworker as a worker who works fully from home. Because of this definition, the percentage of teleworkers before the lockdown is so low (close to zero in 2018, Figure A1). In my opinion, this is a very rigid definition. Do the results hold when partially relaxing the definition of teleworkers (including at least those who report working mostly from home)?*

**Response:**

We intentionally used a rigid definition of remote work to distinguish the labor market outcomes of individuals who fully transitioned into working from home from that of the rest (no or partial WFH)—who would have been subject to more work-related disruptions or health-risks following the COVID-19 outbreak. **Thus, we maintained the rigid definition—only including the full WFH workers—in the revised manuscript (Section 3.2**). Regardless, we acknowledge the value of your comment and have run the main DID estimations with a broader definition of remote work that you suggested. **S8** **Table** below displays another set of results with a binary remote work variable indicating respondents who work fully or partially from home. Comparing the results to that of Table 4 (using the rigid remote work definition) in the manuscript, we see several points of divergence.

In columns (4)-(6) of both **Table 4** and **S8** **Table**, WFH (fully or partially) during the Lockdown leads to an increased monthly income—but only for men and not for women. Yet, while the income gain is still significant in the post-lockdown phase for the full WFH workers (in **Table 4** in the manuscript), the gain dissipates when we include partial WFH workers in the table below. The income gain for remote workers in both tables comes from increased hourly wages, not due to increased work hours.

While the productivity gain seen during the Lockdown heightens in the post-Lockdown phase for the full WFH workers (columns 1-3 of **Table 4**), it reduces—although still statistically significant—when we include partial WFH in the definition (columns 1-3 in **S8** **Table**). Of note, the wage gain is not a result of the increased demands for remotely-conducted occupations: We have controlled for any changes in the occupation-specific demands by including occupation-specific time trends in all estimations. The change in the work environment and productivity seems to be what is driving the change in hourly wage. Despite that WFH is generally associated with a reduction of work hours in both tables, such a reduction applies only to female full WFH workers (columns 7-9 in **Table 4**). When we include partial WFH, the reduction in the work hours is experienced by both genders.

**Overall, we observe that the partial WFH workers’ labor market outcomes are somewhat different from that of the full WFH—with smaller gains in income and a greater reduction in work hours for partial WFH.** Including partial WFH workers in our binary explanatory variable on the remote work arrangements would diffuse the effect of full WFH. **S8** **Table** and these descriptions are now included in the **Supporting Information**.

| **S8 Table.** **Effects of Remote Work Arrangements on Hourly Wage, Monthly Income, and Monthly Work Hours** | | | | | | | | | |
| --- | --- | --- | --- | --- | --- | --- | --- | --- | --- |
|  | (1) | (2) | (3) | (4) | (5) | (6) | (7) | (8) | (9) |
|  | Y=Hourly Wage | | | Y=Monthly Income | | | Y=Monthly Hours Worked | | |
|  | All | Male | Female | All | Male | Female | All | Male | Female |
| (reference = Pre-Lockdown) | | |  |  |  |  |  |  |  |
| **Working Remotely x Lockdown** | 10.58*** | 7.32* | 14.33*** | 277.11*** | 276.40* | 172.54 | -9.56** | -9.18* | -12.46** |
|  | (2.99) | (3.81) | (4.30) | (102.09) | (154.73) | (112.08) | (3.79) | (5.07) | (5.57) |
| **Working Remotely x Post- lockdown** | 9.62*** | 9.96* | 11.28*** | 166.14 | 172.51 | 88.48 | -23.37*** | -17.54* | -31.88*** |
|  | (3.58) | (5.67) | (4.18) | (125.45) | (197.76) | (146.67) | (6.85) | (9.87) | (10.55) |
|  |  |  |  |  |  |  |  |  |  |
| Working Remotely  (1 yes 0 no) | 2.74 | 3.80 | 0.25 | -262.13** | -264.74 | -157.81 | -3.55 | -3.06 | 0.19 |
|  | (3.36) | (4.62) | (4.41) | (109.82) | (176.11) | (114.74) | (6.08) | (8.69) | (9.51) |
|  |  |  |  |  |  |  |  |  |  |
| Lockdown | 6.80** | 9.76** | 3.68 | -160.93* | -174.33 | -99.12 | -3.74 | -10.12* | 3.03 |
|  | (2.65) | (3.96) | (2.60) | (84.83) | (119.75) | (103.83) | (4.18) | (5.43) | (5.65) |
| Post-lockdown | 7.71** | 10.22** | 4.20 | 127.67 | 190.33 | 83.95 | 4.99 | -1.18 | 12.34 |
|  | (3.25) | (4.45) | (3.61) | (95.03) | (135.55) | (116.78) | (6.15) | (7.93) | (7.59) |
|  |  |  |  |  |  |  |  |  |  |
| Individual FE & Occu. FE | Yes | Yes | Yes | Yes | Yes | Yes | Yes | Yes | Yes |
| Occu. time trends | Yes | Yes | Yes | Yes | Yes | Yes | Yes | Yes | Yes |
| Control variables | Yes | Yes | Yes | Yes | Yes | Yes | Yes | Yes | Yes |
| N | 4308 | 2301 | 2007 | 4308 | 2301 | 2007 | 4308 | 2301 | 2007 |
| Notes: Remote is a binary variable for whether the respondent worked fully or partially from home in May 2020 during the COVID-19 lockdown. The reference time period is ‘Pre-lockdown’, between April-July 2018 and December 2019, prior to the COVID-19 pandemic. ‘Lockdown’ refers to March and June 2020, while ‘Post-lockdown’ refers to November 2020, six months after the end of the lockdown. Standard errors, shown in the parentheses, are clustered at the household level. | | | | | | | | | |
| *p<0.1 **p<0.05 ***p<0.01 | | | | | | | | | |

*R2C20. The authors say that during the pandemic, there were people who were able to hold multiple jobs. What percentage of people in the survey are affected by this situation? What is the incidence of moonlighting in Singapore according to official statistics?*

**Response:**

Unfortunately, our survey did not ask about the number of jobs. At the same time, our salary income question did not specify to report only from their primary job—which meant that our respondents reported their total monthly earnings from all jobs they held.

Instead, we could provide information for the entire working population in Singapore (with). According to the 2022 Labour Force in Singapore (Ministry of Manpower, 2022), out of 2,366,200 residents employed in Dec. 2020, 2.8 percent of employed persons in Singapore held multiple jobs.^[4]^ During the pandemic, the government—the National Wage Council in their wage and training guidelines and the Ministry of Manpower in an advisory on second-job arrangements (Ministry of Manpower 2020; TAFEP, 2020)—even encouraged firms to encourage their employees to hold multiple jobs. Aside from the COVID-19 induced fluctuations in 2020-2022, the share of workers practicing moonlighting has been small but on a steady uptrend since 2012. We now have included this information in the **manuscript** (**Section 3.2**)

*R2C21. The essential or non-essential nature of the industry where workers are employed is a very relevant variable for the study of the economic consequences of the pandemic on the labour market. However, the database does not include information on industries. I guess that this is because the questionnaire was originally designed for other purposes before the pandemic (in 2018). While the authors recognize this lack of information, they should put more effort to explain why they consider that not having information on industry would not affect their estimates. Is there any paper specifically addressing the case of Singapore that supports this statement?*

**Response:**

Indeed, our survey did not collect the industry information from our respondents. As elaborated in our response to **Q2** earlier, the original purpose of this survey at its initial rollout in 2018 was focused on family compositions, marriage, health, and fertility—and was later retrofitted to include work and COVID-19 related questions in 2020.

The concern is that the industries would indicate the essential or non-essential nature of work respondents carried out—which, in turn, had implications for their wage ratees as the essential workers experienced soaring demands during the pandemic. However, we believe the bias from missing industry fixed-effects is minimal in our study. While certain industries such as healthcare included more essential workers, not all jobs in the industries were essential. In other words, the essential/non-essential labels as well as any economic consequences of the labels are, in fact, occupation-specific rather than industry-specific. We account the job-specificity by including occupational fixed effects. More importantly, we account for changes in job demands between 2018-2020 (e.g., soaring demands for healthcare frontline workers during the COVID-19) by controlling for **occupation-specific time trends** in all estimations. The sentence on not having industry identifiers is now moved to **Section 6 (discussion)** as one of the study limitations. We have added to the section our rationale for thinking that this poses minimal risks for the current study.

*R2C22. The authors classify occupations as “professionals” and "non-professional”. I guess that this classification is made to differentiate between teleworkable and non-teleworkable occupations. In my opinion, technicians are also teleworkable occupations.*

**Response:**

We have collected information on survey respondents’ current occupation in 30 categories, as shown in **S3 Table** in the Supporting Information (attached below, for your reference). Categories 1-11, which are labeled as ‘professional’ occupations, refer to white-collar, office-based, high-income jobs. The ability to adopt full-remote work arrangements is not the criteria with which we identified professional occupations from the rest. We believe equating ‘professional jobs’ with ‘full remote jobs’ would not be appropriate to examine the impact of job-remoteability on income/wage between professional- and non-professional- subgroups.

Regardless, per your advice, we have now included professionals (categories 1-11) and associate professionals and technicians (categories 12-18) as ‘professionals’ in our heterogeneity analyses.

**S3 Table. Occupational Categories in the Survey**

| **Professionals** | |  | **Clerical Support Workers** |  |
| --- | --- | --- | --- | --- |
| 1 | Legislator, Senior Official and Chief Executives | 19 | Clerical Supervisor |  |
| 2 | Administrative and Commercial Manager | 20 | General and Keyboard Clerk |  |
| 3 | Production and Specialised Services Manager | 21 | Customer Services Officers and Clerk |  |
| 4 | Hospitality, Retail and related Services Manager | 22 | Numerical and Material-Recording Clerk |  |
| 5 | Science and Engineering Professional | 23 | Others ***PLEASE SPECIFY*** |  |
| 6 | Health Professional | **Service and Sales Workers** | |  |
| 7 | Teaching and Training Professional | 24 | Personal Service Worker | |
| 8 | Business and Administration Professional | 25 | Sales Worker | |
| 9 | Information and Communications Technology Professional | 26 | Personal Care Worker | |
| 10 | Legal, Social and Cultural Professionals | 27 | Protective Services Worker | |
| 11 | Others ***PLEASE SPECIFY*** | 28 | Customer Service Worker | |
| **Associate Professionals And Technicians** | | 29 | Others ***PLEASE SPECIFY*** | |
| 12 | Physical and Engineering Science associate professionals | 30 Occupation unknown/ Other | |  |
| 13 | Health Associate Professional |  |  |  |
| 14 | Business and Administration Associate Professional |  |  |  |
| 15 | Legal, Social, Cultural and related Associate Professional |  |  |  |
| 16 | Information and Communications Technician |  |  |  |
| 17 | Teaching Associate Professional |  |  |  |
| 18 | Others ***PLEASE SPECIFY*** |  |  |  |

*R2C23. Analysed sample: The analysed sample is unclear. The authors say that their final sample consists of 4638 observations, involving 781 people (362 women and 419 men). I find the description of the analysed sample very confusing as on page 12 they say that the potential participants are 3038, of which 657 people finally met the criteria to participate. It is understood that the people who finally fill in all the waves are 378 (which I understand are women and they report information about their male spouses). If women report information about men, how is it possible that there is more information from men (419) than from women (362)?*

**Response:**

The description—that the “potential participants are 3038, of which 657 people finally met the criteria to participate,”—is about the eligibility threshold for the survey, and does *not* refer to our final sample used for estimations in this paper. We have now removed the description to avoid confusing the readers, and adjusted our sample eligibility criteria as shown below so that every step could be justified:

The original survey data had 660 female and 660 male respondents—who were present in all 6 survey ways. We dropped 68 person-wave observations who were separated or divorced, leaving 655 men and 655 women in waves 1-5, and 651 each in wave 6. We dropped 3,184 person-wave observations (all observations for 531 individuals across waves) if they did not work in waves 1-2, prior to COVID-19 and Lockdown, as changes across time in their salary earnings or wages cannot be measured. Further excluded were 360 person-wave observations (all observations for 60 individuals across waves)for having worked in waves 1-2 but not working in waves 3-6 in 2020, since the beginning of the COVID-19 pandemic. All remaining observations had reported their remote work statuses as well as monthly salary income, hours worked per month, and hourly wage (i.e., main explanatory variables) and covariates. **Once all sample eligibility criteria were applied,** the **final sample** consisted of 4,308 person-wave observations in an unbalanced panel—with **384 men** (in waves 1-5, with a subsequent exclusion of 3 in wave 6) and **335 women** (in waves 1-5, with a subsequent exclusion of 3 in wave 6) all of whom were married and working throughout the 6 waves and were Singaporean citizens.^[5]^

Our final sample changed from what we used in the original submission due to our dropping **all observations of respondents** if they (1) did not work in waves 1-2 regardless, and (1) worked in waves 1-2 but did not work at all for the entirety of 2020 (waves 3-6). At the time of the initial submission, we made the error of only dropping their person-wave observations with missing information. We have realized that these individuals cannot be included in any wave as their salary income, work hours, and wages could not be tracked across time. *The necessary condition for our sample respondents was that they have records of income/wage before- and after- the start of the COVID-19/Lockdown.*

Going back to your question, we had more male spouses than female spouses in our final sample (384 men, 335 women). In our sample selection process, there existed a possibility for a respondent to be dropped while his or her spouse remained in the sample. For example, only the husband would remain in the sample if we excluded the wife for working in 2020 (waves 3-6), or vice versa. We have now included the step-by-step process on the final sample selection in the **manuscript** (**Section 3.1**).

*R2C24. Since this is an ad-hoc survey, it is very relevant to present information on whether or not the sample is representative of the total population. This is done in Table A2 (Comparison of Baseline Characteristics in the Sample and National Statistics of Married Resident Mothers Aged 30-34). However, it is striking that it compares only its sample of 24-59 year olds with the population aged 30-34. Such a table should compare similar age groups.*

**Response:**

We have now updated the table—it is now **S5** **Table** in the **Supporting Information**—with statistics that match the age range of 25-59. We have provided comparisons between our sample and the population and have listed the partial representativeness of our sample as a study limitation in **Section 6.**

*R2C25. Table 2. It would be necessary to show the data displayed in Table 2 for the 3 periods. It is also necessary to include the number of hours worked in each of the periods.*

**Response:**

We have now updated **Table 2** to show the three periods (pre-Lockdown, during-Lockdown) and included monthly salary income, monthly hours worked, and hourly wages as outcome variables.

*RESULTS*

*R2C26. When analysing the results displayed in Table 4 (DID model estimates) it would be useful to start by commenting on the independent coefficients of “working remotely”, “lockdown” and “post-lockdown”, and then comment on the corresponding interactions terms.*

**Response:**

We have now revised the manuscript so that we also describe the coefficients of ‘remote work’ and ‘lockdown/post-lockdown’—apart from the coefficients of their interaction terms (**Manuscript Section 5.2**). Given the DID estimation set-up, the coefficient of ‘remote work’ will estimate the state of respondents who fully WFH in the pre-lockdown period, and the coefficients of ‘lockdown/post-lockdown’ would refer to respondents who went through the two periods with zero remoteability (i.e., fully working outside).

*R2C27. However, I find the estimation results in Table 4 rather strange. For example, the coefficient of the “lockdown” variable is 189.070 for the male sample, 116.632 for the female sample, but the corresponding coefficient for the pooled sample is -50.246. How can the authors explain this difference?*

**Response:**

The negative coefficient for the pooled sample was an unfortunate typographical error that came out of the formatting period when we moved around the order of the variables presented on the table. We have fixed the issue and we see positive coefficients for the pooled sample as well as for males and females.

*R2C28. I also find striking the coefficient of "working remotely". Figure 2 implies that the monthly wage of teleworkers is higher than the wage of non-teleworkers (before and after the pandemic). However, in the estimates in Table 2, the coefficient of "working remotely" (which measures the difference between teleworkers' and non-teleworkers' wages in the pre-pandemic period), is negative for both men and women. I understand that once the other explanatory factors are controlled for, the sign may change, but this is something the authors should explain adequately in the manuscript.*

**Response:**

We now show pre-trends for hourly wages, monthly income, and monthly work hours in **Table 2, S7 Table** in the **Supporting Information,** and **Figure 2.** As you pointed out, while **Figure 2** implied that the hourly wage and the monthly income of WFH individuals were higher than that of non-WFH workers and the monthly work hours of WFH workers were lower than non-WFH workers’ during the pre-lockdown period, **Table 3** and **S7 Table** showed that the WFH individual’ income/wage were lower and hours were higher than those of non-WFH individuals in the pre-lockdown period (i.e., the coefficients of ‘working remotely’).

The difference between Figure 2 and Table 3/A7 is that the estimations done for Table 3/A7 included individual fixed-effects. Thus, the higher income of WFH respondents captured in the pre-lockdown period in **Figure 2** may suggest that there were differences in the innate abilities of individuals who chose WFH and non-WFH jobs. Indeed, in the pre-Lockdown period, WFH was a choice. It could have been that those who chose WFH pre-Lockdown were generally in higher-earning jobs or were more productive—thereby showing the higher income than those who chose non-WFH jobs. We now have added these discussions in **Section 5.1 (p.27,** *“We identified interesting discrepancies in comparing…”*) of the manuscript.

*R2C29. The authors say that “In our total sample (column 1), we see that fully remote work arrangements under lockdown is associated with an increase in monthly earned income by $245.60. The income further increases by $397.02 even after the lockdown is lifted (i.e., November 2020).” What is the theoretical explanation that causes this ~~wage~~income increase? I guess that this finding is related to the number of working hours, but the authors do not explain this result.*

**Response:**

Per your comments, we have revised the manuscript so that we show the impact of WFH not only on monthly salary income (our original outcome variable) but also on monthly work hours and hourly wage in all estimations (including **Table 4**, whose result was described in the quotation). We find that the increased income comes from the increased wage rates for the WFH individuals and not from the increase in the total hours worked (now detailed in **Section 5.2**). The growth in wage rate seems to have driven from increased performance/productivity of WFH individuals—and not from the increased demands of the WFH jobs during the pandemic. As elaborated earlier, we have controlled for any changes in the occupation-specific demands by including occupation-specific time trends in all estimations. At the same time, the number of working hours fell for WFH workers—although sharply and significantly for only female WFH workers. We have revised the interpretation of these results in **Section 6**.

*R2C30. On the other hand, the effect of the lockdown for teleworkers is the sum of the “lockdown” coefficient plus the interaction term with teleworking (-50 + 245) while for non-teleworkers the effect of the lockdown is just the “lockdown” coefficient (-50). Thus, the pandemic has caused an increase of teleworkers’ wages and a decrease for the non-teleworkers. Again, I think this result is related to the number of working hours. Teleworkers were able to work during the lockdown and non-teleworkers probably reduced the number of working hours (or even do not worked at all) due to the mandatory closures.*

**Response:**

We have revised **Section 5**.**1** and **Section 6** to better discuss the impacts of Lockdown on WFH vs. non-WFH workers as you demonstrated in this question. In our revised results in **Table 4**, we see that the impact of lockdown on monthly income is positive for WFH workers while positive but statistically insignificant for non-WFH workers. While both their wages increase in 2020, that of WFH workers soared substantially more. At the same time, WFH workers experienced a reduction in work hours while non-WFH workers experienced an increase in the hours. We have added these descriptions in **Section 5.1.**

Of note, the old results you mention in the question differ from the new results due to our changed sample. As stated in our response to **R2C18** above, our sample eligibility criteria have been more clearly established. Specifically, we dropped individuals’ all person-wave observations if (1) they were not working in 2018-2019 (waves 1-2) or (2) if they used to work before but stopped working in 2020 (waves 3-6). In our initial submission, we made the mistake of dropping only their observations for not-working periods. In the revised manuscript, we have dropped this person entirely from the sample so that we examine the effect of WFH arrangements *conditional* on that everyone had some working arrangements across all 6 waves (i.e., everyone kept working).

Per your comment on the work hours, we have revised all estimations to show the impact of WFH on monthly income, monthly work hours, and hourly wages.

*R2C31. The authors claim “Time fixed effects are in line with macroeconomic data showing a large dip in economic activity during the lockdown before recovering in November 2020 [Section 5.2]”. However, this statement is not seen in Table 4. For the pooled sample, the “lockdown” coefficient is negative, which would be in line with the previous statement, but for the separate samples, the coefficient of “lockdown” indicates the opposite, as I have mentioned above.*

**Response:**

Apologies for the confusion. The description should have been placed with Table 3 in the original estimation—where we show the estimated coefficients of the time fixed-effects. In our main results (Table 4), we intentionally do not explore the fixed-effects in depth. The pre-trends analytics for monthly income are now moved to **S7 Table (columns 1-3)** in the **Supporting Information**—as we have newly set the hourly wages as the main outcome variable of interest. In Table A7, we confirm that our descriptions still hold: The coefficients for the time fixed-effects in columns 1-3 align with macroeconomic data—showing a sudden decrease in income due to the dip in economic activities in March-June 2020 and a gradual recovery in November 2020. In the manuscript, we have re-arranged the location of our description to the section on pre-trends tables (**Section 5.1**).

*R2C32. Since it is necessary to revise estimations corresponding to the results presented in Table 4, it would also be necessary to revise the estimates presented in Tables 5 to 7.*

**Response:**

Yes, we have now revised all tables, and rewrote the manuscript so that all reporting of results and discussions match the updated results.

*MINOR COMMENTS:*

*- Homegeneise the expression Covid19. In some cases it appears as COVID-19 and in others as Covid19.*

**Response:** We have modified the paper so that we use Covid-19 consistently throughout the document.

*- Page 17. It says Figure 1, it should be* ***S1 Figure*** *(****Supporting Information****).*

**Response:** We have corrected the error on page 17.

**REFERENCES**

Baert S, Lippens L, Moens E, Weytjens J, Sterkens P. The Covid-19 Crisis and Telework: A Research Survey on Experiences, Expectations and Hopes. *IZA Discussion Paper No. 13229, 2020.*

Bosua R, Gloet M, Kurnia S, Mendoza A, Yong J. Telework, productivity and wellbeing: an Australian perspective. *Telecommunications Journal of Australia* 2013; 63(1): 11.1-11.12.

Chew, Hui Min. Resident unemployment could have doubled if not for COVID-19 support measures in 2021: MOF, In ChannelAsia, Feb 17 2022, accessible at: <https://www.channelnewsasia.com/singapore/covid-19-support-measures-jobs-economy-resident-unemployment-mof-report-2503701>

Dalton, M. & Groen, J. A. Telework during the COVID-19 pandemic: estimates using the 2021 Business Response Surve, Monthly Labor Review, Bureau of Labor Statistics US, (2022). Accessible at: <https://www.bls.gov/opub/mlr/2022/article/telework-during-the-covid-19-pandemic.htm>

EngageRocket. The Resilience of Singapore Workforce. 2020 [cited 2024 Jan 17]. Available from: <https://www.engagerocket.co/the-resilience-of-singapore-workforce-in-2020>

Jamison, D. T., & Wu, K. B. (2021). The East–West divide in response to COVID-19. *Engineering*, 7(7), 936–947. <https://doi.org/10.1016/j.eng.2021.05.008>

Kazekami S. Mechanisms to improve labor productivity by performing telework. *Telecommunications Policy*. 2020 Mar 1;44(2):101868.

Liu, Y., Saltman, R. B., & Yeh, M. J. (2023). From bureaucratic administration to effective intervention: Comparing early governmental responses to the COVID-19 virus across East Asian and western health systems. *Health Services Management Research*, 36(3), 193-204

Mellish, T. I., Luzmore, N. J., & Shahbaz, A. A. (2020). Why were the UK and USA unprepared for the COVID-19 pandemic? The systemic weaknesses of neoliberalism: a comparison between the UK, USA, Germany, and South Korea. *Journal of Global Faultlines*, 7(1), 9-45 1.

Ministry of Trade and Industry (2021). ECONOMIC SURVEY OF SINGAPORE 2020, Chapter 2. Accessible at: file:///C:/Users/spplz/Desktop/FullReport_AES2020.pdf

Ministry of Manpower, 2020. National Wages Council 2020/2021 Guidelines. Accessible at: <https://www.mom.gov.sg/newsroom/press-releases/2020/0330-national-wages-council-2020-2021-guidelines>

Ministry of Manpower (2021a). 2020 Labour Force in Singapore. Accessible at: <https://stats.mom.gov.sg/iMAS_PdfLibrary/mrsd_2020LabourfForce.pdf>

Ministry of Manpower (2021b), 2020 Conditions of Employment Survey, accessible at <https://stats.mom.gov.sg/iMAS_PdfLibrary/msd-coe-2020.pdf>

Ministry of Manpower (2022). 2022 Labour Force in Singapore. Accessible at: <https://stats.mom.gov.sg/iMAS_PdfLibrary/mrsd_2022LabourForce.pdf>

Navarro, V. (2021). Why Asian countries are controlling the pandemic better than the United States and Western Europe. *International Journal of Health Services*, 51(2), 261-264

Shokoohi, M., Osooli, M., & Stranges, S. (2020). COVID-19 pandemic: What can the West learn from the East? *International Journal of Health Policy and Management*. <https://doi.org/10.34172/ijhpm.2020.85>

Tripartite Alliance for Fair & Progressive Employment Practices (TAFEP), 2020. Supporting Employees on Second Job Arrangements During COVID-19. Accessible at: <https://www.tal.sg/tafep/resources/articles/2020/supporting-employees-on-second-job-arrangements-during-covid-19>

1. Official statistics from the Ministry of Manpower (2021a) does not divide the occupation-specific distributions by age groups. Thus, the total here differs from the one reported in the previous paragraph that falls within ages 25-64.
2. Ibid.
3. Please see our response to Q15 for the original purpose of the survey, at the time of the baseline (wave 1) in 2018.
4. Of note, Singapore had a total of 3,597,700 employed persons in Dec. 2020. Out of this, 1,231, 500 were foreigners and thus excluded from the calculation (Ministry of Trade and Industry SG, 2021)
5. The citizenship was not a requirement for this study, but was a requirement for survey that was initially carried out in 2018.
