# Supplementary material for: Unequal gains from remote work during COVID-19 between spouses: Evidence from longitudinal data in Singapore
Source: PLoS One. 2025 May 20;20(5):e0324113. doi: 10.1371/journal.pone.0324113 (PMC12091887; doi:10.1371/journal.pone.0324113)
Supplement: Attachment — Submitted filename: referee_report_sub2_jun2024b.docx [file pone.0324113.s020.docx]

**Unequal Gains from Remote Work during COVID-19 between Spouses: Evidence from Longitudinal Data in Singapore**

**Referee report**

**June 2024**

The manuscript has significantly improved from the previous version. I thank the authors for their detailed responses to my comments and suggestions.

Nonetheless, I consider that the paper requires further revision in several aspects.

**Main comments**

1) My first comment is regarding the explanatory variables included in the estimations. In particular, the human capital of individuals (measured by their educational level) is an essential determinant of wages and labour supply. Hence, all estimates should include individuals’ education among the explanatories. But the authors claim that the vector Xit only includes age groups, number of children, and presence of an infant under two years old. Additionally, the estimates for the whole sample should include among explanatories a dummy of gender.
2) When analysing their results, the authors make a lot of comparisons between the results for the female and the male subsamples. Nonetheless, such comparisons are simply based on the coefficients (and their significance level), but they are not supported by any statistical test for the equality of regression coefficients.
3) Authors should homogenize the denomination of the analysed indicators through the manuscript. Many times, when the authors refer to “hourly wages” they simply say “wages”. In addition, is it important to correct the inconsistencies along the manuscript as regards the analysed indicators, because in some parts of the manuscript they only refer to “monthly income” (which was the only indicator analysed in the first version of the manuscript), and the new parts of the manuscript refer to the three indicators analysed in the revised manuscript. For instance, on page 33 the authors say “In a sensitivity check, we consider whether monthly income increased monotonically with the extent of remote work arrangements individuals adopted”). In any case, I consider more appropriate the denomination of “monthly wage” than “monthly income”.
4) I am afraid that Table 4 contains a typo, as the coefficients and standard errors of the variables “lockdown” and “post-lockdown” are exactly the same (whereas in Table 5 the coefficients of these variables are different). Consequently, the analysis in the text referring to these coefficients should also be corrected.
5) In Table 6 there are also results that seem somewhat strange. It could may be a typo?. The coefficients of the interaction term between “remote”, “lockdown” and “receiving any help” (panel C, Table 6) and the corresponding to the interaction term between “remote”, “lockdown” and “spouse working remotely” (panel D) are 0.01 with a standard error of 0.00 in all estimates for panel C and D.
6) Authors claim that they estimate the causal effects of the adoption of remote work arrangements on wages and hours worked as a result of the pandemic. In my opinion this is not true since the authors select a sample of individuals that were employed across six points in time (waves) and compare the results for observations of “fully working from home” and “non fully-working from home”. Particularly, the authors say that “The nation-wide lockdown mandate, observed from 7 April 2020 to 1 June 2020, required workers whose occupational tasks could be carried out remotely to work from home regardless of personal preferences [28]. The mandate was strictly enforced, thereby alleviating issues of self-selection into remote work arrangements”. The mandatory fact of the adoption of telework would allow to analyse the causal effect of telework by defining a treatment group (those who were not working from home before the lockdown but working from home during the lockdown) and two potential control groups: 1) those who were working from home before the lockdown and also during the lockdown (because for them working from home is the result of their preferences), and 2) those who were not working from home before the pandemic nor during the lockdown. If the authors maintain their empirical strategy of comparing fully teleworkers with non-fully teleworkers, I consider they should not claim to be analysing the causal effect.
7) On page 17, the authors claim, *“As our data initially undersampled employed persons, our sample selection strategy, in a way, helped bring our sample’s employment rates to be closer those reported in the official statistics”.* I disagree with this statement because the authors are introducing a significant selection bias by excluding from the analysis all persons who have not been employed at some point in time.
8) Table 2. It seems quite strange that the percentage of teleworkers during the period of lockdown is lower than that in the pre-pandemic period (56.12%, 51.64% and 18.67% among females; the male sample display a similar path). In my opinion, the explanation for this phenomenon is somewhat superficial and not supported by their data (“…possibly due to job switches or changes in the number of jobs held.”). They should explore further to give a substantiated explanation. I am afraid it is due to the sample selection. I would expect an increase in the percentage of teleworkers over the total number of employed people in the May 2020 survey (without excluding any employed individual) compared to the corresponding percentage in the April 2018 survey.

**Other comments:**

- The objective of the paper described on page 4 (first paragraph) is not clear. I suggest reviewing its redaction.
- As I suggested in my first report, the authors have included the percentage of workers working from home during the pandemic. Still, they do not report the information on the corresponding figures before the pandemic (page 3). It would be convenient to report it. In Western countries, the percentage of workers working from home has increased significantly compared with the figures before the pandemic. I guess that something similar could have happened in Singapore.
- Database description:

The database description is more precise in this version of the manuscript.

Perhaps the description could be even clearer by explaining that the final sample consists of 335 females and 384 males who were employed during the 6 waves of the survey, which implies 2007 observations for women and 2301 observations for men.

When describing the database and the timing of the data collection (pages 14-15), the authors state that there were three surveys: April 2018, May 2020, and November 2020. However, on page 14, they claim they added questions about COVID-19 at the end of 2019. If I have understood correctly, they added the questions in the May 2020 survey collecting retrospective information about December 2019.

The manuscript also contains many inconsistencies regarding the time of the survey. It is important to homogenise the survey periods. For instance, in some parts of the manuscript, they say that the first survey was collected in April 2018, but in other parts, they say April-July 2018 (even in the axis of Figure 2).

It is clear in the Conclusions section that individuals surveyed are all females and that they report the information on their male spouses. I suggest indicating this in the main text (maybe on page 22 when referring to the participants in the survey).

- Page 9: When referring to the work of Atkin et al., it would also be helpful to indicate based on which data (country) the work of Atkin et al. was carried out (similar to what is done when referring to the work of Gibbs et al.).
- Page 21: The authors say that in their sample, the mean value of the number of children is 1.5 children. I suggest indicating its distribution instead of the mean value (for instance: no children, one child, two children, three or more). Despite this, it is unclear if all females in the sample have children because the authors do not mention this feature in the sample selection description, but they say, “Using an individual-level panel dataset of married spouses with children” in the Discussion section. The authors should clarify this.
- Table 2: The dependent variables (hourly wages, monthly earnings and monthly hours worked) should appear disaggregated for teleworkers and non-teleworkers.

It would also be helpful to display the distribution of workers following the classification used in the sensitivity analysis (whether working fully from home, mostly from home, half from home and half outside the home, mostly outside the home or fully outside the home.)

On the other hand, since the persons surveyed along the six waves are the same, it is strange that the nationality of persons surveyed in the post-lockdown period are somewhat different. Similarly, it is very strange that the percentage of women with children under two years is 1% in the pre-pandemic period but increases up to 40% in the lockdown period. It seems that the question in the questionary of April 2018 was different than that in May 2020 and November 2020.

- The analysis of the results should be more thorough. For instance, the authors claim, “The estimated coefficients of the standalone “remote work” variable suggest that the WFH arrangements lowered income for both genders (a decrease of $393.37 for men and $53.75 for women) even though the coefficient is significant only among men (columns 4-6).” These coefficients do not indicate a drop; they indicate that before the pandemic, teleworkers earned lower wages than non-teleworkers. Other sentences display similar inconsistencies.

**Minor comments**

- Page 7: The authors say, “Employment rate also turned positive in the last quarter of 2020.” Nevertheless, the data in Figure 1 seems negative in 2020.Q4, and it turns positive in the first quarter of 2021. There is also a typo; they should say “employment growth” (or “employment growth rate”) instead of “employment rate” (the employment rate is the percentage of employed persons over the total population aged over 16 years old).
- It is necessary to homogenise the vertical and horizontal axis in Figure 1 for GDP growth and employment growth.
- Page 11: The figures in the first sentence are not clear.
- Table 1: The note 2 under Table 1 says “across four points in time”. I understand that it should be “across three points in time”.
- Page 12 and 17: The expression “during this period” is duplicated in the sentence “During this period, schools and childcare centers were closed during this period as the law mandated ……. “ (page 12). The sentence “Further excluded were 360 observations (all observations for 60 individuals) for having worked in waves 1-2 but stopped working in waves in 2020” is duplicated. (page 17)
- Page 30: The title of section 5.2 should be actualized.
