# Supplementary material for: Unequal gains from remote work during COVID-19 between spouses: Evidence from longitudinal data in Singapore
Source: PLoS One. 2025 May 20;20(5):e0324113. doi: 10.1371/journal.pone.0324113 (PMC12091887; doi:10.1371/journal.pone.0324113)
Supplement: Attachment — Submitted filename: 0_Response to Reviewers - R&R2nd.docx [file pone.0324113.s023.docx]

**RESPONSE TO REVIEWERS**

PLOS ONE

Dear Editor,

Many thanks to you and the reviewers for helpful comments on the paper, Ref: **PONE-D-24-02155R1**, “Unequal Gains from Remote Work during COVID-19 between Spouses: Evidence from Longitudinal Data in Singapore.” In order to accommodate all comments and suggestions, we have undertaken major revisions to all sections of the paper.

**Academic Editor: José Alberto Molina**

Thank you for submitting your manuscript to PLOS ONE. After careful consideration, we feel that it has merit but does not fully meet PLOS ONE’s publication criteria as it currently stands. Therefore, we invite you to submit a revised version of the manuscript that addresses the points raised during the review process. Please submit your revised manuscript by **Aug 24 2024 11:59PM**. If you will need more time than this to complete your revisions, please reply to this message or contact the journal office at [plosone@plos.org](mailto:plosone@plos.org). Please include the following items when submitting your revised manuscript:

- A rebuttal letter that responds to each point raised by the academic editor and reviewer(s). You should upload this letter as a separate file labeled 'Response to Reviewers'.
- A marked-up copy of your manuscript that highlights changes made to the original version. You should upload this as a separate file labeled 'Revised Manuscript with Track Changes'.
- An unmarked version of your revised paper without tracked changes. You should upload this as a separate file labeled 'Manuscript'.

If you would like to make changes to your financial disclosure, please include your updated statement in your cover letter. Guidelines for resubmitting your figure files are available below the reviewer comments at the end of this letter.

If applicable, we recommend that you deposit your laboratory protocols in protocols.io to enhance the reproducibility of your results. Protocols.io assigns your protocol its own identifier (DOI) so that it can be cited independently in the future. For instructions see: [https://journals.plos.org/plosone/s/submission-guidelines#loc-laboratory-protocols](https://ddec1-0-en-ctp.trendmicro.com/wis/clicktime/v1/query?url=https%3a%2f%2fjournals.plos.org%2fplosone%2fs%2fsubmission%2dguidelines%23loc%2dlaboratory%2dprotocols&umid=3e98892c-d3d8-4329-bbb7-7244cc6ea052&auth=8d3ccd473d52f326e51c0f75cb32c9541898e5d5-444806a7363d48304281403e548e6df11e0af0fd). Additionally, PLOS ONE offers an option for publishing peer-reviewed Lab Protocol articles, which describe protocols hosted on protocols.io. Read more information on sharing protocols at [https://plos.org/protocols?utm_medium=editorial-email&utm_source=authorletters&utm_campaign=protocols](https://ddec1-0-en-ctp.trendmicro.com/wis/clicktime/v1/query?url=https%3a%2f%2fplos.org%2fprotocols%3futm%5fmedium%3deditorial%2demail%26utm%5fsource%3dauthorletters%26utm%5fcampaign%3dprotocols&umid=3e98892c-d3d8-4329-bbb7-7244cc6ea052&auth=8d3ccd473d52f326e51c0f75cb32c9541898e5d5-69957e20e1507ef3f1ec3cc4b65f98f48c448f15).

We look forward to receiving your revised manuscript.

Kind regards,

José Alberto Molina
Academic Editor
PLOS ONE

**Reviewer #1**

*Comments to the Author*

*The authors properly addressed the points I previously mentioned in most cases, and the paper becomes much clearer indeed. I have two additional comments and clarifications that come from graphs the authors added or modified.*

**Response:**

Your comments have been incredibly helpful in improving the paper. We have endeavored to address all your comments, and will be happy to address any remaining comments or suggestions that you may have.

*MAJOR POINTS*

*R1C1. It is quite interesting that female teleworkers reduce their hours worked although their hourly wage increases. Then, the natural question is* ***why they do so.*** *I imagined that it was because of more housework or childcare burdens. However, based on Tables S9-1 and S9-2, neither of them increases that much. For example, focus on the coefficient on “Working Remotely x Post-lockdown.” Table 4 says -49.34. First of all, is this -49.34 hours per month? That’s too large! They must increase some other activities because they have a lot more time available. However, Tables S9-1 and S9-2 say that they don’t really increase childcare time or time spent on household chores. We could say the latter increases a bit for some groups, but definitely not enough to spend 49.34 more hours per month. On which activity do they spend time? Is there any way to investigate this more using time use data you might have in your data? Otherwise, the authors’ argument “find that chore responsibilities imposed a negative effect on the female remote workers’ income” may not sound the first order importance given the huge size of the effect. Note that the same thing is true for the group “Working remotely (Fully from home)” even though the average results are driven by this group.*

**Response:**

Thank you for the comment. Regarding the usage of teleworkers’ increased time at hand—due to the female teleworkers’ reduced work hours by 49.34/month—during the post-lockdown period, we do believe that *most of the newly available time is put into childcare.* It should perhaps be highlighted that the results in **Table 4** are shown in *hours per month*, while the results in **Tables S9-1** and **S9-2** are shown in *minutes per hour*. Thus, assuming that the increase of 3.59 minutes/hour in childcare for female WFH workers (shown in **Table S9-1**) is measured over a full day, this translates into an increase of around 86.16 minutes/day, or **43.08 hours/month.** This number is very close to the reduction of work hours by 49.34/month. We have now added these points to **6.Discussion Section.**

We unfortunately are not able to empirically explore other usages of the newly available time of WFH workers from the reduced work hours. In our survey questionnaire, we did not ask beyond the spouses’ time spent on childcare and chores. This prevents us from exploring changes in time usages in other non-work activities. Anecdotally, WFH workers during the lockdown recall being extremely busy due to strict public health-related administrative duties at home (e.g., filling out health declaration forms daily for all family members if anyone in the family has COVID-19). Another aspect of at-home responsibilities that took up a considerable amount of time for WFH workers was elderly care, which is not surprising given that about 55 percent of senior population aged 65 or above in Singapore reside with their children in 2020 (Wing et al. 2022). While the responsibilities for both of these activities heightened significantly during the pandemic, such activities would not have been captured in our survey data as it centered on time usages in childcare and chores (cooking/cleaning, as listed in the survey). We now have included this point as a part of the limitation of the study in the **Discussion Section**.

*R1C2. The same effect size issues are applied for the results – in* ***Table 4****, how can male teleworkers' wage increase by $20/hour given their pre-lockdown mean is $30 from Table 2? I understand that it is not fair to just say that the effect size is too large to be trusted because it might be true, but I encourage authors to describe how hourly wages change in a firm in Singapore in general. Is it realistic to have a trend in wages in Figure 2 in terms of the magnitude? For example, some news articles reporting a huge increase in wages in some specific firms in post-COVID period would help readers believe these results. There must be some news or anecdotal evidence if the effect is actually such a big size.*

**Response:**

Thank you for the question. Initially, we suspected that the increase in the hourly wage was in part due to the inflation in 2020. Thus, we tried using real monthly income and real hourly wage, converting them into 2019 dollars using Singapore Consumer Price Index (<https://www.singstat.gov.sg/whats-new/latest-news/cpi-highlights>). The results—analogous to Table 4—are displayed below. As can be seen in the table below, the COVID-19 induced inflation does not seem to be the main driver behind the steep increase in the hourly wage. Specifically, we observe that WFH arrangements in the post-lockdown period is associated with an increase of hourly wages by $20.06/hour for male WFH workers and by $12.21/hour for female WFH workers—*very similar* to the increase of $20.04/hour for male WFH workers and $12.20/hour for female WFH workers shown in **Table 4** of the manuscript.

| **Table 4A.** **Effects of Remote Work Arrangements on Real Hourly Wage, Real Monthly Income, and Monthly Work Hours** | | | | | | | | | |
| --- | --- | --- | --- | --- | --- | --- | --- | --- | --- |
|  | (1) | (2) | (3) | (4) | (5) | (6) | (7) | (8) | (9) |
|  | Y=Real Hourly Wage | | | Y=Real Monthly Income | | | Y=Monthly Hours Worked | | |
|  | All | Male | Female | All | Male | Female | All | Male | Female |
| (reference = Pre-Lockdown) | | | |  |  |  |  |  |  |
| **Working Remotely x Lockdown** | **8.45***** | **7.26*** | **8.74*** | **274.95**** | **391.62**** | **67.68** | **-9.54**** | **-6.99** | **-12.68**** |
|  | **(3.14)** | **(3.71)** | **(5.18)** | **(106.64)** | **(177.98)** | **(120.02)** | **(3.72)** | **(5.11)** | **(5.21)** |
| **Working Remotely x Post- lockdown** | **14.80***** | **20.06**** | **12.21**** | **269.47*** | **450.65**** | **53.83** | **-34.87***** | **-20.61*** | **-49.34***** |
|  | **(4.83)** | **(8.81)** | **(5.78)** | **(148.76)** | **(225.96)** | **(185.67)** | **(9.49)** | **(11.12)** | **(15.15)** |
|  |  |  |  |  |  |  |  |  |  |
| Working Remotely (1 yes 0 no) | -1.44 | -2.37 | -0.87 | -267.33** | -396.97** | -55.06 | 5.67 | -5.05 | 17.44* |
|  | (3.55) | (5.48) | (4.43) | (103.73) | (185.84) | (118.91) | (5.38) | (7.30) | (9.11) |
|  |  |  |  |  |  |  |  |  |  |
| Lockdown | 9.41*** | 10.96*** | 8.79*** | -109.14 | -173.85 | -10.80 | -5.27 | -12.53** | 1.05 |
|  | (2.53) | (3.89) | (2.43) | (75.92) | (106.30) | (91.10) | (4.00) | (5.04) | (5.49) |
| Post-lockdown | 7.93*** | 9.39** | 6.46** | 143.56 | 158.71 | 139.10 | 4.36 | -4.11 | 12.85* |
|  | (2.81) | (4.06) | (3.11) | (90.27) | (128.58) | (105.49) | (5.66) | (6.51) | (7.63) |
|  |  |  |  |  |  |  |  |  |  |
| Individual FE, Occupation FE, Time FE | Yes | Yes | Yes | Yes | Yes | Yes | Yes | Yes | Yes |
| Occupation FE x Time FE | Yes | Yes | Yes | Yes | Yes | Yes | Yes | Yes | Yes |
| Control variables | Yes | Yes | Yes | Yes | Yes | Yes | Yes | Yes | Yes |
| N | 4308 | 2301 | 2007 | 4308 | 2301 | 2007 | 4308 | 2301 | 2007 |
| Notes: Wage and income have been converted to 2019 dollars. Remote is a dichotomous variable for whether the respondent worked fully from home in May 2020 during the COVID-19 lockdown. The reference time period is ‘Pre-lockdown’, between April-July 2018 and December 2019, prior to the COVID-19 pandemic. ‘Lockdown’ refers to March and June 2020, while ‘Post-lockdown’ refers to November 2020, six months after the end of the lockdown. Standard errors, shown in the parentheses, are clustered at the household level. | | | | | | | | | |
| *p<0.1 **p<0.05 ***p<0.01 | | | | | | | | | |

We believe the increase in hourly wage is also somewhat mechanical in nature. For the reasons elaborated in **R1Q1** above, the monthly work hours of remote workers decreased significantly by 20.61/month for WFH men and 49.34/month for WFH women. At the same time, real monthly income increased for both genders that adopted full WFH arrangements—thereby leading to the increased hourly wage, *which we calculated using the monthly income and monthly hours worked*. This does not necessarily mean that the actual hourly wage rate in their primary jobs soared.

Additionally, Singapore government launched several COVID-19 relief policies that enabled full-time workers adopting WFH arrangements to *retain* *their jobs and monthly income* even if they could not work as many hours from home—due to loss of productivity, blurred boundaries between work and responsibilities at home, etc. One such policy is Singapore’s Job Support Scheme (JSS), which encouraged employers to retain employees by funding 75% of the salaries—capped at the first SGD $4,600—of Singaporeans and permanent residents during April and May 2020 and by launching a tier-based funding (75% for industries directly hit by COVID-19, 25-50% for the rest of the industries) for the remaining 15 months until March 2021 (IRAS Singapore, n.d.; Seow, 2020). Furthermore, while the total monthly work hours decreased, it could have been that the remote workers started multiple part-time, high-paying jobs starting in 2020. Case in point, the government of Singapore encouraged individuals to seek multiple jobs during the pandemic to enhance their economic security (Ministry of Manpower 2020). Both of the policies mentioned above would have helped maintain/increase monthly income despite a reduction in the self-reported work hours—thereby increasing the hourly wage we calculated. We now have added these elaborations on the magnitude of the increase in the hourly wage of WHF workers in the **6. Discussion Section.**

Lastly, as for the magnitude of the hourly wage increase (i.e., $20.04/hour for male WFH workers) in the post-lockdown period, we believe the increase should be compared not to the average hourly wage of total male sample ($30/hour) shown in **Table 2** but instead to the average hourly wage of male WHF workers, now shown in **Table S10** to be about **$35/hr.**

**Reviewer #2**

*Comments to the Author*

*The manuscript has significantly improved from the previous version. I thank the authors for their detailed responses to my comments and suggestions.*

*Nonetheless, I consider that the paper requires further revision in several aspects.*

**Response:**

Your comments have been tremendously helpful in improving the paper. We have endeavored to address all your comments, and will be happy to address any remaining comments or suggestions that you may have.

*MAIN COMMENTS:*

*R2C1. My first comment is regarding the explanatory variables included in the estimations. In particular, the human capital of individuals (measured by their educational level) is an essential determinant of wages and labour supply. Hence, all estimates should include individuals’ education among the explanatories. But the authors claim that the vector Xit only includes age groups, number of children, and presence of an infant under two years old. Additionally, the estimates for the whole sample should include among explanatories a dummy of gender.*

**Response:** ­­­­­­­­­­­­

Thank you for the comment. While we fully agree that the human capital of individuals is an important determinant, the reason that it is not shown in the results is that it has been differenced out as we deployed the fixed-effect estimator. In fact, the effects of all demographic traits that are time-invariant across the analytic period (education, race, and gender) could not be estimated. What remains in the vector ***X****_it_* are time-varying ones.

We have decided to keep the fixed-effect estimator as the main estimation model used in our paper because we believe it is crucial that we account for individual fixed-effects in the model: Individuals’ income, wage, and work hours (our outcome variables) as well as the extent of remote work arrangements they have adopted (explanatory variable) are dictated by their choice of occupation, which in turn is under the influence of unobserved preferences and abilities. Not accounting for such preferences/abilities will introduce bias to the model.

*R2C2. When analysing their results, the authors make a lot of comparisons between the results for the female and the male subsamples. Nonetheless, such comparisons are simply based on the coefficients (and their significance level), but they are not supported by any statistical test for the equality of regression coefficients.*

**Response:**

Thank you. We have now specified whether the cross-gender differences in the estimated coefficients are statistically significant in **Tables 4-7** in the manuscript. Descriptions of the results have been updated accordingly.

*R2C3. Authors should homogenize the denomination of the analysed indicators through the manuscript. Many times, when the authors refer to “hourly wages” they simply say “wages”. In addition, is it important to correct the inconsistencies along the manuscript as regards the analysed indicators, because in some parts of the manuscript they only refer to “monthly income” (which was the only indicator analysed in the first version of the manuscript), and the new parts of the manuscript refer to the three indicators analysed in the revised manuscript. For instance, on page 33 the authors say “In a sensitivity check, we consider whether monthly income increased monotonically with the extent of remote work arrangements individuals adopted”). In any case, I consider more appropriate the denomination of “monthly wage” than “monthly income”.*

**Response:**

In the second version of the manuscript, additional outcome variables were introduced to provide more nuanced analyses on what was causing the changes in monthly income (i.e., whether the income changed due to increased/decreased work hours or hourly wages), per first reviewer’s suggestions.

We now have revised the text to clearly specify and distinguish ‘monthly income’, ‘hourly wages’, and ‘monthly work hours’. We also ensured that we mention all three outcome variables in the description of the tables.

*R2C4. I am afraid that Table 4 contains a typo, as the coefficients and standard errors of the variables “lockdown” and “post-lockdown” are exactly the same (whereas in Table 5 the coefficients of these variables are different). Consequently, the analysis in the text referring to these coefficients should also be corrected.*

**Response:**

Thank you very much for spotting the typological errors. These were indeed—very unfortunate—formatting errors. We now have revised **Table 4** to display the correct coefficients. Text was revised as well.

*R2C5. In Table 6 there are also results that seem somewhat strange. It could may be a typo?. The coefficients of the interaction term between “remote”, “lockdown” and “receiving any help” (panel C, Table 6) and the corresponding to the interaction term between “remote”, “lockdown” and “spouse working remotely” (panel D) are 0.01 with a standard error of 0.00 in all estimates for panel C and D.*

**Response:**

Thank you. Sincere apologies for the formatting errors, and we greatly appreciate your locating our mistakes. Yes, they should have been updated into non-zero numbers. We now have correctly specified the estimated coefficients in **Table 6**, Panel C and D. Text has been updated as well.

*R2C6. Authors claim that they estimate the causal effects of the adoption of remote work arrangements on wages and hours worked as a result of the pandemic. In my opinion this is not true since the authors select a sample of individuals that were employed across six points in time (waves) and compare the results for observations of “fully working from home” and “non fully-working from home”. Particularly, the authors say that “The nation-wide lockdown mandate, observed from 7 April 2020 to 1 June 2020, required workers whose occupational tasks could be carried out remotely to work from home regardless of personal preferences [28]. The mandate was strictly enforced, thereby alleviating issues of self-selection into remote work arrangements”. The mandatory fact of the adoption of telework would allow to analyse the causal effect of telework by defining a treatment group (those who were not working from home before the lockdown but working from home during the lockdown) and two potential control groups: 1) those who were working from home before the lockdown and also during the lockdown (because for them working from home is the result of their preferences), and 2) those who were not working from home before the pandemic nor during the lockdown. If the authors maintain their empirical strategy of comparing fully teleworkers with non-fully teleworkers, I consider they should not claim to be analysing the causal effect.*

**Response:**

Thank you for the elaborations. Per your advice, we have done away with the claim that we measure the causal effect.

*R2C7. On page 17, the authors claim, “As our data initially undersampled employed persons, our sample selection strategy, in a way, helped bring our sample’s employment rates to be closer those reported in the official statistics”. I disagree with this statement because the authors are introducing a significant selection bias by excluding from the analysis all persons who are unemployed.*

**Response:**

Thank you. Indeed, we dropped from our final sample all person-wave observations for (1) individuals who *did not* work in waves 1-2, in 2018-2019, and (2) individuals who worked in waves 1-2 but *stopped working* in waves 3-6 in 2020, and (3) individuals who stayed *unemployed/out of the labor force* for all 6 waves. As stated in the manuscript, in **Section 3.1 Data**, this sample eligibility criteria was set to distinguish the effect of remote work adoption from that of job displacement/acquisition across waves. Including individuals who *did not* work in waves 1-2, in 2018-2019, would have introduced an upward bias (positive bias) to the effect of remote work arrangements because if such individuals engaged in WFH jobs in 2020, their income and wages would have experienced a steep jump from 0. This is not necessarily be the labour market outcome associated with the adoption of WFH arrangements. Rather, this would be the outcome associated with transitioning from the non-working state to the working state. Similarly, including in the sample individuals who worked in waves 1-2 but *stopped working* in waves 3-6 in 2020 would have generated a downward bias. Moreover, individuals without jobs in 2020 cannot be categorized based on the extent of WFH they have adopted—which is our main regressor of interest. In essence, we are measuring the effect of adopting remote work arrangements *conditional on individuals’ continued employment* *throughout the analytic period.* We now have added these descriptions in **Section 3.1 Data*.***

While we wholeheartedly acknowledge your concern that dropping individuals who become unemployed or stopped working in 2020 may introduce selection bias, we would like to highlight that this group comprises a small share of the total sample. Specifically, we dropped 360 person-wave observations across 6 survey waves (i.e., approximately 60 unique individuals), which is about 8.3% of our final sample (N=4,308). Unlike in many other countries, COVID-19 induced unemployment was not a widely-observed phenomenon in Singapore—especially for the Singaporean nationals, permanent residents, and long-term visa holders.^[1]^ This was due to government policies that ensured continued employment during the pandemic. One such policy is Singapore’s Job Support Scheme (JSS), which encouraged employers to retain employees by funding 75% of the salaries—capped at the first SGD $4,600—of Singaporeans and permanent residents during April and May 2020 and by launching a tier-based funding (75% for industries directly hit by COVID-19, 25-50% for the rest of the industries) for the remaining 15 months until March 2021 (IRAS Singapore, n.d.; Seow, 2020). We have now added these in our discussion of the study limitations in the **7. Conclusion Section**.

Lastly, as for the statement to which you referred (*“As our data initially undersampled employed persons, our sample selection strategy, in a way, helped bring our sample’s employment rates to be closer those reported in the official statistics”*), per your advice, we have now removed the statement from the manuscript.

*R2C8. Table 2. It seems quite strange that the percentage of teleworkers during the period of lockdown is lower than that in the pre-pandemic period (56.12%, 51.64% and 18.67% among females; the male sample display a similar path). In my opinion, the explanation for this phenomenon is somewhat superficial and not supported by their data (“…possibly due to job switches or changes in the number of jobs held.”). They should explore further to give a substantiated explanation. I am afraid it is due to the sample selection. I would expect an increase in the percentage of teleworkers over the total number of employed people in the May 2020 survey (without excluding any employed individual) compared to the corresponding percentage in the April 2018 survey.*

**Response:**

Per your advice, we have examined more closely whether individuals transition from WFH arrangements in the pre-pandemic period to non-WFH arrangements during/post-lockdown, and why. As we studied the effect of WFH arrangements on the labor market outcomes of individuals *conditional* on the fact that individuals stay employed throughout the analytic period, a possible reason for the switch from WFH to non-WFH states could be—as we stated in the manuscript—due to *job switching*. In our final sample, out of a total of 267 unique respondents (1,602 person-wave observations in 6 survey waves) transitioned from WFH to non-WFH states in 2020, none of them switched *occupational* *categories*. Of note, we had asked about respondents’ occupations in 33 aggregate occupational categories shown in **S3 Table**. Given the aggregated categories, this did not mean the individuals did not switch jobs or job-tasks within their original industry/occupational category.

It should also be reminded that, given the definition of our WFH variable, the ‘non-WFH’ state did *not* mean working outside. Our ‘WFH’ indicator identified those who worked fully from home, while leaving out those working at least partially outside. As you know, our actual WFH question included five levels of WFH adoption (working fully outside, mostly outside, half from home and half outside, mostly from home, and fully from home) as shown in **Table 5**. Out of 267 unique individuals, 141 went from working fully remotely in the pre-pandemic period (WFH) to working mostly from home (non-WFH)—while staying in the same occupational categories. 73 out of 267 individuals transitioned from working fully remotely to working ‘half from home and half outside.’ 24 out of 267 respondents moved from working fully remotely to working mostly outside. Lastly, 29 out of 267 respondents transitioned from full WFH to working fully outside.

Curiously, the occupational distributions of these groups of individuals that ended up in varying stages of non-WFH were not too different. A plurality of each group consisted of individuals in white-collar occupations (e.g., in business and administration professions, ICT professions, clerical, and legal, social, and cultural professions), even though the groups that transitioned to slightly more non-WFH states included some individuals in sales, customer services, healthcare, hospitality, and production. It could have been that there were demand-driven reassignment of manpower within companies or reconfiguration of job tasks to account for COVID19 related labor market hardships (e.g., manpower shortages in certain industries and certain occupations) that changed the extent to which respondents could engage in full WFH arrangements. Alternatively, it could have been that respondents started working multiple jobs to financially support themselves in 2020—which was actually a practice encouraged by the government of Singapore (Ministry of Manpower 2020). Unfortunately, we were not able to check this because we did not directly ask in the survey the number of jobs held at each point in time (while asking for the total salary income and total work hours across all jobs). We now have added these additional details in **3.3 Summary Statistics Section**.

*OTHER COMMENTS*

*R2C9. The objective of the paper described on page 4 (first paragraph) is not clear. I suggest reviewing its redaction.*

**Response:**

The paragraph is now revised. Thank you.

*R2C10. As I suggested in my first report, the authors have included the percentage of workers working from home during the pandemic. Still, they do not report the information on the corresponding figures before the pandemic (page 3). It would be convenient to report it. In Western countries, the percentage of workers working from home has increased significantly compared with the figures before the pandemic. I guess that something similar could have happened in Singapore.*

**Response:**

Unfortunately, the share of workers working from home before the pandemic is not publicly available in Singapore. Reports on remote work arrangements start from 2020 and onward. Instead of remote work, Monetary Authority of Singapore (2023) kept track of the share of establishments offering ‘flexible work arrangements’—which is a broader notion that encompasses remote work arrangements—and reported that such establishments jumped from less than 50 percent in 2014 to over 90 percent in 2021. We have now added this information in the manuscript, in **Introduction**.

*R2C11. Database description: The database description is more precise in this version of the manuscript. Perhaps the description could be even clearer by explaining that the final sample consists of 335 females and 384 males who were employed during the 6 waves of the survey, which implies 2007 observations for women and 2301 observations for men. When describing the database and the timing of the data collection (pages 14-15), the authors state that there were three surveys: April 2018, May 2020, and November 2020. However, on page 14, they claim they added questions about COVID-19 at the end of 2019. If I have understood correctly, they added the questions in the May 2020 survey collecting retrospective information about December 2019. The manuscript also contains many inconsistencies regarding the time of the survey. It is important to homogenise the survey periods. For instance, in some parts of the manuscript, they say that the first survey was collected in April 2018, but in other parts, they say April-July 2018 (even in the axis of Figure 2). It is clear in the Conclusions section that individuals surveyed are all females and that they report the information on their male spouses. I suggest indicating this in the main text (maybe on page 22 when referring to the participants in the survey).*

**Response:**

RE your first suggestion: Thank you, but we have the exact statement in the last sentence of Section **3.1 Data** (“*In the end, our final sample consisted of 4,308 person-wave observations in an unbalanced panel with 384 men (in waves 1-5, with a subsequent exclusion of 3 respondents in wave 6) and 335 women (in waves 1-5, with a subsequent exclusion of 3 respondents in wave 6)—all of whom were married and employed during the entire 6 waves*”).

As for the description on **page 14**, as you correctly pointed out, we added the COVID-19 questions for subsequent survey waves, first of which came in May 2020. We have now revised the description to avoid confusion. The inconsistencies regarding the timing of the survey have also been revised (stating April-July 2018 instead of April 2018). Lastly, we revised the description in Section 3.1 Data to clearly state how the male spouses’ information was collected. Specifically, we now write “*The survey followed 660 female participants—all of whom were married, Singaporean residents, and were able to read, write, and speak in English—who reported information on themselves as well as their 660 spouses (all male). As we pulled the information on the respondents’ spouses into distinct observations, our data consisted of a total of 3,960 person-wave observations for each gender in 6 waves.*”

*R2C12. Page 9: When referring to the work of Atkin et al., it would also be helpful to indicate based on which data (country) the work of Atkin et al. was carried out (similar to what is done when referring to the work of Gibbs et al.).*

**Response:**

Thank you. The sources of data for both papers are now stated on page 9.

*R2C13. Page 21: The authors say that in their sample, the mean value of the number of children is 1.5 children. I suggest indicating its distribution instead of the mean value (for instance: no children, one child, two children, three or more). Despite this, it is unclear if all females in the sample have children because the authors do not mention this feature in the sample selection description, but they say, “Using an individual-level panel dataset of married spouses with children” in the Discussion section. The authors should clarify this.*

**Response:**

In our sample, the number of children ranges between 0 and 7. The sentence in the Discussion section has been revised—removed ‘with children’—to more accurately depict our sample. Not everyone has children at all points in the analytic period. We now show the distribution of children instead of the mean value in **Table 2**, using the groupings you suggested.

*R2C14. Table 2: The dependent variables (hourly wages, monthly earnings and monthly hours worked) should appear disaggregated for teleworkers and non-teleworkers. It would also be helpful to display the distribution of workers following the classification used in the sensitivity analysis (whether working fully from home, mostly from home, half from home and half outside the home, mostly outside the home or fully outside the home.) On the other hand, since the persons surveyed along the six waves are the same, it is strange that the nationality of persons surveyed in the post-lockdown period are somewhat different. Similarly, it is very strange that the percentage of women with children under two years is 1% in the pre-pandemic period but increases up to 40% in the lockdown period. It seems that the question in the questionary of April 2018 was different than that in May 2020 and November 2020. The analysis of the results should be more thorough. For instance, the authors claim, “The estimated coefficients of the standalone “remote work” variable suggest that the WFH arrangements lowered income for both genders (a decrease of $393.37 for men and $53.75 for women) even though the coefficient is significant only among men (columns 4-6).” These coefficients do not indicate a drop; they indicate that before the pandemic, teleworkers earned lower wages than non-teleworkers. Other sentences display similar inconsistencies.*

**Response:**

Given the number of additional variables for which we provided the summary statistics (i.e., outcome variables by WFH statuses and worker distribution for detailed WFH statuses), we have pulled them out into a separate table, **S10 Table: Descriptive Statistics for Labour Market Outcomes by Detailed WFH Categories.** Descriptions in the manuscript have also been updated accordingly.

| **S10 Table. Descriptive Statistics for Labour Market Outcomes by Detailed WFH Categories** | | | | | | |
| --- | --- | --- | --- | --- | --- | --- |
|  | Male | | | Female | | |
|  | Pre-Lockdown | Lockdown | Post- Lockdown | Pre-Lockdown | Lockdown | Post- Lockdown |
| *Outcome variables, disaggregated* | | |  |  |  |  |
| Hourly wages (SGD), for full WFH | 34.95 | 50.17 | 61.37 | 27.83 | 44.83 | 41.57 |
|  | (18.10) | (50.87) | (59.86) | (19.89) | (88.03) | (52.50) |
| Monthly income (SGD), for full WFH | 6275.86 | 6604.27 | 7000.00 | 4410.90 | 4510.60 | 4645.16 |
|  | (3248.60) | (3591.88) | (3704.35) | (2165.03) | (2443.45) | (2761.54) |
| Monthly Hours Worked, for full WFH | 181.92 | 164.36 | 161.30 | 170.43 | 155.11 | 139.87 |
|  | (29.01) | (66.22) | (75.37) | (57.27) | (91.01) | (79.97) |
| Hourly wages (SGD), for partial or non-WFH | 28.19 | 36.28 | 37.20 | 23.38 | 31.01 | 31.08 |
|  | (23.20) | (55.19) | (42.20) | (10.68) | (40.81) | (27.98) |
| Monthly income (SGD), for partial or non-WFH | 4983.26 | 4779.18 | 5627.91 | 3925.17 | 3859.05 | 4322.22 |
|  | (2804.37) | (2817.60) | (3111.57) | (1708.60) | (1785.45) | (2124.79) |
| Monthly Hours Worked, for partial or non-WFH | 190.80 | 180.16 | 185.57 | 170.28 | 164.68 | 171.05 |
|  | (53.38) | (82.82) | (72.77) | (26.16) | (64.88) | (79.64) |
| *Independent variable, disaggregated* | | |  |  |  |  |
| Working Fully Outside (%) | 24.48 | 25.52 | 35.17 | 11.04 | 12.74 | 23.80 |
|  | (43.02) | (43.62) | (47.81) | (31.37) | (33.35) | (42.65) |
| Working Mostly Outside (%) | 8.33 | 9.20 | 17.06 | 5.37 | 6.17 | 10.24 |
|  | (27.66) | (28.92) | (37.67) | (22.57) | (24.07) | (30.36) |
| Working Half from Home (%) | 13.80 | 14.67 | 22.31 | 13.73 | 13.83 | 23.80 |
|  | (34.51) | (35.40) | (41.69) | (34.44) | (34.54) | (42.65) |
| Working Mostly from Home (%) | 15.63 | 16.06 | 15.75 | 13.74 | 15.62 | 23.49 |
|  | (36.33) | (36.73) | (36.47) | (33.20) | (36.32) | (42.46) |
| Working Fully from Home (%) | 37.76 | 34.55 | 9.71 | 56.12 | 51.64 | 18.67 |
|  | (48.51) | (47.57) | (29.65) | (49.66) | (50.00) | (39.03) |
| Total person-wave observations | 768 | 1152 | 381 | 670 | 1005 | 332 |

As for the nationality (I believe you are referring to race, where we identified respondents into Chinese, Indian or Malay), they change slightly over time due to our having an unbalanced panel. As we described in Section 3.1 Data (pp.16-17), our sample selection criteria led to a slight reduction of both genders in the final wave—which would have caused the race distribution to change slightly in the post-lockdown period.

As for the variable on ‘having children under two years (yes/no)’, we used the same question in the questionnaire—which asked respondents in each survey wave to identify age of all their children. Given that the survey respondents were all married women most of whom were in their prime childbearing years and that the original purpose of the survey was to collect information on the respondents’ fertility, children and spouses, plans to have children, etc. (please see **Section 3.1 Data**, first paragraph), it is not surprising to see a plurality of them (37-43%) of them having given birth since the start of the pandemic. It should also be reminded that there is a temporal gap of 2 years between the ‘pre-lockdown’ and ‘lockdown’ periods—which is significantly wider than the gap of few months between the ‘lockdown’ and ‘post-lockdown’ periods. The steep increase could have also been spurred by Singapore’s Baby Bonus Scheme—a payment of SGD $11,000 bonus for having first two children (each) and $13,000 for the third and thereafter—intended to boost births during the pandemic (Government of Singapore, 2024).

Lastly, **5. Results Section** have been revised to include more careful analysis of the findings. Thank you.

*MINOR COMMENTS*

*R2C15. Page 7: The authors say, “Employment rate also turned positive in the last quarter of 2020.” Nevertheless, the data in Figure 1 seems negative in 2020.Q4, and it turns positive in the first quarter of 2021. There is also a typo; they should say “employment growth” (or “employment growth rate”) instead of “employment rate” (the employment rate is the percentage of employed persons over the total population aged over 16 years old).*

**Response:**

As you have correctly pointed out, employment growth turns positive in the following quarter—in the first quarter of 2021. Yes, this is the employment growth rate, not the rate of employment. We have edited the manuscript to correct the errors.

*R2C16. It is necessary to homogenise the vertical and horizontal axis in Figure 1 for GDP growth and employment growth.*

**Response:**

We now have homogenized the axes in panels A-C, Figure 1.

*R2C17. Page 11: The figures in the first sentence are not clear.*

**Response:**

We have now edited the sentence.

*R2C18. Table 1: The note 2 under Table 1 says “across four points in time”. I understand that it should be “across three points in time”.*

**Response:**

We have fixed the typo, thank you.

*R2C19. Page 12 and 17: The expression “during this period” is duplicated in the sentence “During this period, schools and childcare centers were closed during this period as the law mandated ……. “ (page 12). The sentence “Further excluded were 360 observations (all observations for 60 individuals) for having worked in waves 1-2 but stopped working in waves in 2020” is duplicated. (page 17)*

**Response:**

The sentence on page 12 has been revised. The sentence on page 17 has been removed to avoid redundancy.

*R2C20. Page 30: The title of section 5.2 should be actualized.*

**Response:**

Since we have changed the contents of the analyses to focus not only on monthly income but hourly wages and work hours (per other reviewer’s request), we have slightly modified the title of section 5.2 to more accurately reflect the section. Thank you.

**REFERENCES**

Government for Singapore, 2024. Baby Bonus Scheme, in Made for Families. Accessible at: <https://www.madeforfamilies.gov.sg/support-measures/raising-your-child/financial-support/baby-bonus-scheme>

IRAS Singapore. Jobs Support Scheme (JSS). [website] Accessible at: <https://www.iras.gov.sg/schemes/disbursement-schemes/jobs-support-scheme-(jss)>

Ministry of Manpower. National Wages Council 2020/2021 Guidelines [Internet]. 2020 [cited 2024 Apr 26]. Available from: <https://www.mom.gov.sg/newsroom/press-releases/2020/0330-national-wages-council-2020-2021-guidelines>

Monetary Authority of Singapore, 2023. Annual Report 2023/2024. Chapter on Labour Market and Inflation. Accessible at: <https://www.mas.gov.sg/publications/annual-report/2024/annual-report-2023-2024>

Seow, Joanna. 2020. askST: 7 things to know about Jobs Support Scheme payouts. In Strait Times. Accessible at: <https://www.straitstimes.com/singapore/manpower/jobs-support-scheme-payouts-start-7-things-to-know>

Wing, W. K., Lin, W. W., & Huimin, F. (2022). Demographic Profile of Seniors in Singapore. In Statistics Singapore Newsletter, Issue 1. Accessible at: <https://www.singstat.gov.sg/-/media/files/publications/population/ssn122-pg6-9.ashx>

1. Of note, our survey interviewed Singaporeans residents only.
