# Supplementary material for: Unequal gains from remote work during COVID-19 between spouses: Evidence from longitudinal data in Singapore
Source: PLoS One. 2025 May 20;20(5):e0324113. doi: 10.1371/journal.pone.0324113 (PMC12091887; doi:10.1371/journal.pone.0324113)
Supplement: Attachment — Submitted filename: 0_Response to Reviewers - R&R_3rd.docx [file pone.0324113.s025.docx]

**RESPONSE TO REVIEWERS**

PLOS ONE

Dear Editor,

Many thanks to you and the reviewers for helpful comments on the paper, Ref: **PONE-D-24-02155R2**, “Unequal Gains from Remote Work during COVID-19 between Spouses: Evidence from Longitudinal Data in Singapore.” In order to accommodate all comments and suggestions, we have undertaken major revisions to all sections of the paper.

**Academic Editor: José Alberto Molina**

Thank you for submitting your manuscript to PLOS ONE. After careful consideration, we feel that it has merit but does not fully meet PLOS ONE’s publication criteria as it currently stands. Therefore, we invite you to submit a revised version of the manuscript that addresses the points raised during the review process. Please submit your revised manuscript by **Aug 24 2024 11:59PM**. If you will need more time than this to complete your revisions, please reply to this message or contact the journal office at [plosone@plos.org](mailto:plosone@plos.org). Please include the following items when submitting your revised manuscript:

- A rebuttal letter that responds to each point raised by the academic editor and reviewer(s). You should upload this letter as a separate file labeled 'Response to Reviewers'.
- A marked-up copy of your manuscript that highlights changes made to the original version. You should upload this as a separate file labeled 'Revised Manuscript with Track Changes'.
- An unmarked version of your revised paper without tracked changes. You should upload this as a separate file labeled 'Manuscript'.

If you would like to make changes to your financial disclosure, please include your updated statement in your cover letter. Guidelines for resubmitting your figure files are available below the reviewer comments at the end of this letter.

If applicable, we recommend that you deposit your laboratory protocols in protocols.io to enhance the reproducibility of your results. Protocols.io assigns your protocol its own identifier (DOI) so that it can be cited independently in the future. For instructions see: [https://journals.plos.org/plosone/s/submission-guidelines#loc-laboratory-protocols](https://ddec1-0-en-ctp.trendmicro.com/wis/clicktime/v1/query?url=https%3a%2f%2fjournals.plos.org%2fplosone%2fs%2fsubmission%2dguidelines%23loc%2dlaboratory%2dprotocols&umid=3e98892c-d3d8-4329-bbb7-7244cc6ea052&auth=8d3ccd473d52f326e51c0f75cb32c9541898e5d5-444806a7363d48304281403e548e6df11e0af0fd). Additionally, PLOS ONE offers an option for publishing peer-reviewed Lab Protocol articles, which describe protocols hosted on protocols.io. Read more information on sharing protocols at [https://plos.org/protocols?utm_medium=editorial-email&utm_source=authorletters&utm_campaign=protocols](https://ddec1-0-en-ctp.trendmicro.com/wis/clicktime/v1/query?url=https%3a%2f%2fplos.org%2fprotocols%3futm%5fmedium%3deditorial%2demail%26utm%5fsource%3dauthorletters%26utm%5fcampaign%3dprotocols&umid=3e98892c-d3d8-4329-bbb7-7244cc6ea052&auth=8d3ccd473d52f326e51c0f75cb32c9541898e5d5-69957e20e1507ef3f1ec3cc4b65f98f48c448f15).

We look forward to receiving your revised manuscript.

Kind regards,

José Alberto Molina
Academic Editor
PLOS ONE

**Reviewer #1**

*All comments have been addressed.*

**Response:**

Thank you very much for your guidance.

******************************************************************************

**Reviewer #2**

*Comments to the Author*

*The manuscript has improved from the previous version, and the authors have addressed the majority of my comments and suggestions. I thank the authors for their detailed responses. I have some additional comments, clariﬁcations, and corrections of several inconsistencies that require further revision.*

**Response:**

Thank you very much for your guidance throughout the review process. We have modified our paper per your comments, and are happy to address suggestions that you may have.

*MAIN COMMENTS:*

**R2Q1:** RE Table 2 - As I suggested in my previous report, the most relevant information for the paper's objectives should appear in the main text. In particular, part of the information that is now displayed in the new Table S10 and Table S9 should be included in Table 2. I agree that Table 2 is too long, but in my opinion the numbers in parenthesis (are they SD? it is not indicated what they are) could be eliminated. It is not usually to display the SD for frequencies. Logically, this comment does not apply to the endogenous variables. Hence, the endogenous variables should appear disaggregated for teleworkers and non-teleworkers (the ﬁrst 6 rows from Table S10). Also, the time dedicated to childcare and chores should appear disaggregated for teleworkers and non- teleworkers.
**Response:**

Thank you. Yes, the numbers in parenthesis are standard deviations. For binary indicators (frequencies), we have now removed the standard deviations. We added rows in Table 2 to show the summary statistics for the endogenous variables (i.e., the outcome variables and the time use in chores and childcare) separately for teleworkers and non-teleworkers. Description of the table was updated accordingly.

**R2Q2:** The explanation oﬀered by the authors as regards the decrease in the percentage of teleworkers in the lockdown period and the post-lockdown period is not convincing. There is not only a switch from “Working Fully from Home” to “Working Mostly from Home”, but the most preoccupant part is that their data show an increase in the percentage of workers who declare “working Fully outside” or “mostly outside” (from 32.81% in the pre-lockdown period to 34.72% in lockdown and 52.23% in post-lockdown among males). Something similar occurs for females since the percentage of women who declare “working fully outside” or “mostly outside” increases from 16.41% in the pre-lockdown period to 18.91% in the lockdown and 34.04% in the post-lockdown period. This characteristic of their sample selection should be indicated as an additional limitation of their conclusions. Additionally, the authors start their motivation in the abstract with the sentence, “The COVID-19 outbreak and the rise of remote work generated distinct labor market outcomes for workers based on their occupation and the ability to adapt to changes”, when their data show precisely the contrary pattern.
**Response:**

In our manuscript submitted for the last round of review, we stated that there was a slight drop in the shares of full WFH between the pre-lockdown to during lockdown for both genders (37.8% to 34.6% for males, 56.1% to 51.6% for females). The share drops significantly for both genders in the post-lockdown (down to 9.7% for males, and 18.7% for females). Given our definition of the full WFH indicator, a switch from full WFH to an alternate state includes multiple possibilities: switching from (1) full WFH to (2) mostly from home, (3) half-and-half, (4) mostly outside, or (5) fully outside. Regardless of whether individuals went from (1) to (2), (1) to (3), (1) to (4), or (1) to (5) across the analytic period, none of these individuals changed their (aggregate) occupational categories (See **Table A3**). In other words, if an individual was a health professional in pre-lockdown or during lockdown, even if this person switched from fully WFH state to working mostly outside in post-lockdown, this person stayed a health professional. For these individual, the reason for the change in the extent of WFH adoption may be due to position change within their broad occupational category (e.g., health professional), or a change in the nature of job tasks demanded in their position. Another important change in Singapore’s labor market is the government’s endorsement and encouragement of the multiple-job holding practice—which used to be discouraged or banned before the pandemic. Even if an individual who was a clerical support worker (e.g., general and keyboard clerk) in the pre-pandemic period stayed a clerical support worker during lockdown and in post-lockdown, if this person got a second job that required outside delivery services, for instance, this person would mark himself as ‘not fully WFH’ (i.e., working at least partially outside). While the change in the number of jobs held by individuals in each time period cannot be checked as our survey did not include such a question, this is indeed a possibility that could partially explain the reason for a sudden rise in the share of ‘not fully WFH’ workers in the post-lockdown period.

Here, because we only took into consideration those that switched from (1) to another state (2-5), our explanation does *not* apply to individual who stayed (4 or 5) across the analytic periods. In other words, as you rightfully noted, we had not provided an explanation for the sudden increase in the share of those that stayed (4 or 5) in the three time periods. Now, part of the sudden increase in this group’s share in post-lockdown may simply driven by the lifting of the policy. During the lockdown, those whose job could be done remotely *had* to be done remotely. When this policy was lifted, many resumed outside operations (partially, mostly, or fully). The rest of the switching may be due to job sorting, though we are unable to investigate whether or why individuals choose/transition into jobs that could potentially pose health risks (i.e., jobs that are done mostly or fully outside). We now include this characteristic of the sample selection as a limitation in the **Conclusion** of the manuscript, **at the end of page 50.**

We also have revised the starting sentence of the abstract to “The COVID-19 outbreak and the rise of remote work *could have generated* distinct labor market outcomes for workers based on their occupation and the ability to adapt to changes.” We meant to start with a conjecture (not a conclusive statement), which then would be tested by our research question. Thank you for the guidance.

| Table A3: Occupational Categories in the Survey | | | |
| --- | --- | --- | --- |
| **Professionals** | |  | **Clerical Support Workers** |
| 1 | Legislator, Senior Official and Chief Executives | 19 | Clerical Supervisor |
| 2 | Administrative and Commercial Manager | 20 | General and Keyboard Clerk |
| 3 | Production and Specialised Services Manager | 21 | Customer Services Officers and Clerk |
| 4 | Hospitality, Retail and related Services Manager | 22 | Numerical and Material-Recording Clerk |
| 5 | Science and Engineering Professional | 23 | Others ***PLEASE SPECIFY*** |
| 6 | Health Professional | **Service and Sales Workers** | |
| 7 | Teaching and Training Professional | 24 | Personal Service Worker |
| 8 | Business and Administration Professional | 25 | Sales Worker |
| 9 | Information and Communications Technology Professional | 26 | Personal Care Worker |
| 10 | Legal, Social and Cultural Professionals | 27 | Protective Services Worker |
| 11 | Others ***PLEASE SPECIFY*** | 28 | Customer Service Worker |
| **Associate Professionals And Technicians** | | 29 | Others ***PLEASE SPECIFY*** |
| 12 | Physical and Engineering Science associate professionals | 30 | Occupation unknown/ Other ***PLEASE*** |
| 13 | Health Associate Professional |  |  |
| 14 | Business and Administration Associate Professional |  |  |
| 15 | Legal, Social, Cultural and related Associate Professional |  |  |
| 16 | Information and Communications Technician |  |  |
| 17 | Teaching Associate Professional |  |  |
| 18 | Others ***PLEASE SPECIFY*** |  |  |

**R2Q3:** The decrease in the percentage of people working from home is coherent with the occupational changes observed during the lockdown and the post-lockdown periods. In their data, the percentage of professionals and associates professionals (the most teleworkable occupations according to the international empirical evidence) decreases during the lockdown and the post- lockdown period. In contrast, the percentage of service workers clearly increases. Once more, this characteristic of their sample selection is contrary to the empirical evidence of the pandemic on the labour market: the pandemic provoked employment losses in non-teleworkable occupations. Could it be a problem of attrition in their database? The authors claim that they exclude individuals who were not employed in any of the 6 waves from the panel. Does the same apply to “missing individuals”? In that case, it could be feasible that some individuals who have been excluded from the panel are employed individuals (maybe teleworkers) who were not available to respond to the survey.

**Response:**

Singapore’s labor market is unique in that, as can be seen from the table below (extracted from the Ministry of Manpower’s Labour Force in Singapore 2020, Table 35), the managerial, professional, and associate professional job holders comprise a stark majority of the nation’s population ranting between 52%-60% between 2010 and 2020. Clerical support job-holders and service job holders—which often comprise a majority in other developed countries—together make up only 20% of the nation’s labor force. Given the magnitude of the professionals and associate professionals in Singapore, a decrease in their share during the lockdown would translate into an increase in the share of clerical or service workers—as the former professionals and associate professionals would move into these jobs. Since we did not see an increase in the share of clerical workers under lockdown, the effect of the out-migration from the professional/associate professional jobs would be seen in the (increased) number of service job holders.

Furthermore, in 2020, the government of Singapore tried to support service-oriented sectors by subsidizing workers’ wages under the Jobs Support Scheme (JSS) (Ministry of Finance SG, 2020), as the services industry was expected to be most impacted by the pandemic. This policy would have further facilitated the growth in the number of service workers. We have now added some of these explanations in the manuscript, in describing **Table 2** results.

| **Table: Employed residents aged fifteen years and over by occupation, 2010-2020 (June)** | | | | | | | | | | | |
| --- | --- | --- | --- | --- | --- | --- | --- | --- | --- | --- | --- |
| **(in thousands)** | **2010** | **2011** | **2012** | **2013** | **2014** | **2015** | **2016** | **2017** | **2018** | **2019** | **2020** |
| *Panel A: RAW NUMBERS* | | | | | | | | | | | |
| Total | 1,962.9 | 1,998.9 | 2,040.6 | 2,056.1 | 2,103.5 | 2,147.8 | 2,165.3 | 2,175.3 | 2,203.7 | 2,230.4 | 2,222.6 |
| (1) managers and administrators | 352.4 | 367 | 359 | 360.5 | 351.2 | 355.8 | 337.5 | 348.8 | 341.6 | 364.9 | 368.3 |
| (2) professionals | 375.7 | 348.5 | 369.9 | 371.9 | 383.3 | 404.1 | 419.8 | 437.6 | 449.2 | 469.4 | 504.2 |
| (3) associate professionals and technicians | 303.4 | 337.1 | 340.4 | 359.5 | 391.5 | 408.3 | 438.1 | 436.2 | 465 | 467.9 | 458.2 |
| (4) clerical support workers | 243.6 | 249.3 | 265.7 | 271.8 | 268.4 | 267.7 | 244.9 | 247.9 | 241 | 237.2 | 215.6 |
| (5) service and sales workers | 234.5 | 234.8 | 252.5 | 244.7 | 244.3 | 251.6 | 262.4 | 248 | 262.7 | 257.7 | 252.3 |
| (6) craftsmen and related trade workers | 86.5 | 89.5 | 90.6 | 82.2 | 82.2 | 83.9 | 79.2 | 76 | 70 | 67.8 | 62.5 |
| (7) plant, machine operators, and assemblers | 152.7 | 157.5 | 148.7 | 146.9 | 145.9 | 146.1 | 151.1 | 151.5 | 147.9 | 151.4 | 151.7 |
| (8) cleaners, labourers, and related workers | 145.5 | 145.7 | 145.3 | 150.2 | 166 | 160.3 | 160.7 | 160.5 | 156.4 | 149.4 | 149.5 |
| (9) others | 68.7 | 69.6 | 68.6 | 68.7 | 70.8 | 70 | 71.6 | 68.9 | 69.8 | 64.8 | 60.4 |
| *Panel B: PERCENTAGE* | | | | | | | | | | | |
| (1) + (2) + (3) | 52.5 | 52.7 | 52.4 | 53.1 | 53.5 | 54.4 | 55.2 | 56.2 | 57.0 | 58.4 | 59.9 |
| (4) | 12.4 | 12.5 | 13.0 | 13.2 | 12.8 | 12.5 | 11.3 | 11.4 | 10.9 | 10.6 | 9.7 |
| (5) | 11.9 | 11.7 | 12.4 | 11.9 | 11.6 | 11.7 | 12.1 | 11.4 | 11.9 | 11.6 | 11.4 |
| (6) + (7) | 12.2 | 12.4 | 11.7 | 11.1 | 10.8 | 10.7 | 10.6 | 10.5 | 9.9 | 9.8 | 9.6 |
| (8) + (9) | 10.9 | 10.8 | 10.5 | 10.6 | 11.3 | 10.7 | 10.7 | 10.5 | 10.3 | 9.6 | 9.4 |
| Source: Ministry of Manpower, *Labour Force in Singapore 2020*, Table 35. Accessible at: https://stats.mom.gov.sg/iMAS_PdfLibrary/mrsd_2020LabourfForce.pdf | | | | | | | | | | | |

Lastly, while we excluded individuals who were not employed in any of the 6 waves, we kept in our data individuals who stopped responding in the subsequent waves as long as they responded at least twice (including the baseline survey). In other words, as long as individuals were panel responders—which allowed us to conduct panel estimations—we kept their responses even if they stopped responding in the later waves.

**R2Q4:** I miss a brief explanation (similar to what the authors explained in the responses to my previous report) regarding the huge increase in the percentage of women having an infant aged two years or younger (from 1% to 40% during lockdown/post-lockdown).

**Response:**

The survey responses in our data do suggest that (1) a large share of mothers had children just over age 2 in the first two waves (2018-2019), and (2) there were lots of births in 2019 and 2020, which contributed to the growth in the share of mothers with infant(s) aged two or younger. Some of the increase in childbirth could be understood in light of the fact that the majority of female respondents in our data were married and in their late 20s and 30s—at the peak of their fertility timeline. There also were a few government policies encouraging childbirth that were newly introduced in 2019-2020, including the Baby Support Grant (e.g., a one-time grant introduced for eligible parents who gave birth, which complemented the pre-existing Baby Bonus Scheme) (Raguraman, 2023), enhanced CPF Housing Grant that made housing more affordable for first-time homebuyers and thereby encouraging family formation (Liew, 2024), increased preschool subsidies (Ministry of Social and Family Development SG, 2023), and the Intra-Uterine Insemination co-funding scheme to financially support couples facing fertility challenges (Ministry of Health, 2025). However, we do acknowledge that the rise in the share of mothers with infant over two years is indeed steep and is potentially suggestive of selection in the sample. We now have included this as part of the limitation in the final section of the manuscript.

Another possibility is respondents’ misreporting of the age of their children in the baseline wave. Even if this could be a case, we believe that our main results (i.e., effects of remote work arrangements and the government-mandated lockdown) are not affected in any significant manner as the presence of an infant variable was used as one of many control variables in our estimations.

**R2Q5:** On page 24, the authors claim that “For instance, while similar shares of men and women were in professional (about 80%) and associate professional (10%) jobs, more women were in clerical jobs (8% as opposed to 2% among men) which could be easily adopt full remote work arrangements.” This statement is not true since the international literature has shown that professionals and associate professionals are more teleworkable than clerical workers.

**Response:**

Apologies for the confusion. In the statement, we were not comparing clerical jobs to professional/associate professional jobs. Instead, we meant to compare clerical jobs (highlighting their greater remoteability) to service jobs that often require in-person contacts. We now have revised the sentence to better communicate our message.

**R2Q6:** As regards the comments in Table 2, it should be noted that the gender gap in the time devoted to childcare has been reduced during the lockdown and the post-lockdown, while the gender gap in the time devoted to chores has increased.

**Response:**

We now include this observation in our description of the summary statistics in Table 2 (first paragraph, page 26).

**R2Q7:** Finally, I consider that Table 2 should contain tests of diﬀerences in means between males and females.

**Response:**

We now include results from the test of statistically significant (p<0.05) differences between males and females—specific to each analytic period.

**R2Q8 -** **Explanatory variables (Page 27):** After reading the explanation of the independent variables included in the vector X_it_, it seems that it includes industries. However, in the conclusions section, the authors clarify that they do not have information on industry. This aspect should also be clariﬁed in the description of the X_it_ vector.

**Response:**

Indeed, our survey did not include questions on industries. In the previous version of the manuscript, we stated that the vector of covariates ($\boldsymbol{X}_{it}$) did *not* include industries—containing only time-invariant sociodemographic characteristics (age groups, number of children, and presence of an infant). Additionally, we included individual-, occupation-, and time- fixed effects as well as an interaction between occupation and time fixed-effects to control for the occupation-specific time trends. We further elaborated in the manuscript that no industry fixed-effects were include in the estimations.

**R2Q9 –** **Results:** There are some inconsistencies in the interpretation of the results. As I indicated in my second report, the interpretation of the “Remote work” coeﬃcient in Table 4 is not correct. Since the reference category in estimates is the pre-lockdown period, the coeﬃcient of “remote work” measures the diﬀerence in hourly wages, monthly income, and monthly hours worked between teleworkers and non-teleworkers in the pre-lockdown period. Please revise the comments on this coeﬃcient.

**Response:**

Thank you for pointing out the error in our interpretation. We have now revised the description of the estimated coefficient of ‘remote work’ in Table 4.

**R2Q10:** The interpretation of the coeﬃcients of the interaction terms (third paragraph on page 33) seems somewhat confusing. In my opinion, it would be more precise to start the explanation with the authors' comments in the second paragraph on page 34, where they correctly interpret the magnitude of the eﬀect of the lockdown (and the post-lockdown) on teleworkers and non-teleworkers. Attending to the authors' correct statement on page 34, during the lockdown period, male WFH workers experience income gains of $204.93 (and $596.46 during post- lockdown), while non-WFH workers experience income losses by -$182.50 (and income gains by $150.04 in the post-lockdown). The interaction term "Working remotely x Lockdown" allows to test the null hypothesis that the eﬀect of the lockdown does not depend on the type of workers (teleworkers vs. non-teleworkers). The coeﬃcient of the interaction term tests whether the lockdown has had a diﬀerential eﬀect on teleworkers and non-teleworkers. Hence, the value of the interaction term ($387.43 for men) measures the (statistically signiﬁcant) diﬀerential eﬀect of the lockdown on the monthly wages between male teleworkers and non-teleworkers ($204.93 - (-$182.50)=$387.43). Comments on other interaction terms and those in Table 5 have to be revised in the same line.

**Response:**

We have now revised and rearranged the order of the paragraphs per your advice.

**R2Q11:** On page 38, the authors claim, “This was consistent with the pattern seen in Table 4, once again suggesting the sub-par nature of jobs that were done from home before COVID-19 and the global popularization of remote work”. This explanation should be indicated when analysing the results in Table 4.

**Response:**

We have now added the explanation in our description of the results in Table 4.

**R2Q12:** Regarding Table 6, the authors indicate that “The standalone components of the 3-way interaction were included in the estimations but were not shown”. The table, including the 3- way interactions, should be displayed as a supplementary table.

**Response:**

We now show the estimated coefficients for the three-way and two-way interactions and their standalone variables in S11 Table in the Appendix.

**R2Q13:** On page 38, the authors highlighted, “Interestingly, among women, those working mostly outside also experienced a rise in monthly income during the lockdown.” Nevertheless, the coeﬃcient is not statistically signiﬁcant, so they cannot conclude the above statement.

**Response:**

In this statement, we were referring to Table 5, column 6 (the first coefficient). The coefficient for women working ‘mostly outside’ during the lockdown is statistically significant.

**R2Q14:** On page 44, in the discussion section, it is not clear where the ﬁgures for “the increased monthly income generated by adopting WFH arrangements under lockdown/post-lockdown constitutes 8.2-9.5% growth for men while comprising mere 1.2-1.5% (statistically insigniﬁcant) growths for women” come from.

**Response:**

We have further elaborated how we derived the numbers in the Discussion Section.

**R2Q15:** On page 46, the authors state that “Despite the above-mentioned diﬀerences across gender, the mediator analyses lends support for Hypothesis 3 regarding the role of chore burdens, availability of external help for housework, and availability of WFH spouses on dictating the extent of income and productivity(wage) gains of the remotely working individuals.” It would also be convenient to indicate that they do not ﬁnd evidence regarding the role of childcare.

**Response:**

We now have revised the sentence to indicate that the mediating role of childcare responsibilities was not significant.

**R2Q16:** Among the study's limitations, it should also be recognised that the sample is underrepresented in terms of employment.

**Response:**

In the limitation, we now state that, *“…Our data oversamples respondents who are highly-educated, professional, and high-income. As we set sample eligibility to individuals who stay employed throughout the survey waves, we underrepresents those who become unemployed or are out of the labor market in 2020. These may have overestimated the labor market outcomes of full WFH workers in our study, future research endeavors addressing the current data limitations and re-evaluating our research questions would help strengthen our findings.”*

**Other comments:**

**R2Q17:** It is necessary to homogenise the monetary units across the paper, which appear as SGD, S$, or $.

**Response:**

The manuscript has been revised to maintain a homogeneity of the monetary units.

**R2Q18:** On page 22, I guess there is a typo: substitute “they were fully WFH: For both genders,” with “they were fully WFH. For both genders,”

**Response:**

The typo has now been corrected.

**R2Q19:** Review the web links in the references list. For instance, the link https://ddec1-0-en-ctp.trendmicro.com:443/wis/clicktime/v1/query?url=https%3a%2f%2fwww.ilo.org%2fresource%2fbrief%2fworking%2dhome%2destimating%2dworldwide%2dpotential&umid=a880cef5-4a99-4b32-8382-32bbeef4057f&auth=8d3ccd473d52f326e51c0f75cb32c9541898e5d5-7fdfc8c15b7a7ec6a34be9b849dafc80b8b79288 does not exist any more.

**Response:**

All reference links have now been checked, and any inactive ones have been updated.

**R2Q20:** The authors refer to references 8 and 10 on page 3. I am afraid that the reference 10 (A D Michael; Groen,Jeﬀrey. Bureau of Labor Statistics. [cited 2024 Apr 26]. Telework during the COVID-19 pandemic: estimates using the 2021 Business Response Survey : Monthly Labor Review: U.S. Bureau of Labor Statistics) does not say nothing about Singapore.

**Response:**

We have now corrected the reference.

**R2Q21:** The wording of the last sentence in the ﬁrst paragraph on page 7 is confusing. I guess there is a typo, and I wonder if it should be two sentences instead of one.

**Response:**

Per your advice, we have revised the paragraph.

**R2Q22:** The dates of the second interview on page 15 (second paragraph) are inconsistent: May 2020 vs. May-June 2020.

**Response:**

It should have been May 2020. We have now revised the dates.

**R2Q23:** Page 17: Substitute “61.44% of the total of 3,960 females” for “61.44% of the total of 3,960 female person-wave observations”.

**Response:**

We have revised the sentence accordingly.

**R2Q24:** It should be indicated under Tables which statistical test is used to contrast the equality of the coeﬃcients of the male and female subsamples.

**Response:**

For testing the statistical equality of the coefficients from the regressions of the male and female subsamples, we used the *Seemingly Unrelated Estimation Test*, which combine and compare parameter estimates from distinct regression models while accounting for the potential correlations between the error terms of the models. We have now added these details under Tables.

**R2Q25:** On page 41, indicate panel A, panel B, and panel C when commenting on the results from Table 6 (the word “panel” is missing in some of the parenthesis).

**Response:**

The word “panel” is added to all parts in the paragraph on page 41.

**REFERENCES**

Liew, Isabelle. $4.5 billion in housing grants given out from 2020 to 2023: HDB. In The Strait Times, Feb 12, 2024. [Internet] Accessible at: <https://www.straitstimes.com/singapore/housing/45-billion-in-housing-grants-given-out-from-2020-to-2023-hdb>

Ministry of Finance SG. More than 140,000 employers will receive over $7 billion payouts to cover wages of over 1.9 million local employees. Press Releases on 15 Apr 2020. [internet]. Accessible at: <https://www.mof.gov.sg/news-publications/press-releases/more-than-140-000-employers-will-receive-over-7-billion-payouts-to-cover-wages-of-over-1.9-million-local-employees>

Ministry of Health Singapore. 2025. Marriage and Parenthood Schemes. [internet] Accessible at: <https://www.moh.gov.sg/managing-expenses/schemes-and-subsidies/marriage-and-parenthood-schemes>

Ministry of Social and Family Development Singapore. 2023. Progress of the Early Childhood Sector in the Past Decade. [Internet] Accessible at: <https://www.msf.gov.sg/media-room/article/progress-of-the-early-childhood-sector-in-the-past-decade>

Raguraman, Anjali. 13,000 parents to receive $3,000 Baby Support Grant payout by July. In *The Strait Times*, Jun 21, 2023. [internet] Accessible at: <https://www.straitstimes.com/singapore/13000-parents-to-receive-3000-baby-support-grant-payout-by-july>
