# Supplementary material for: Unequal gains from remote work during COVID-19 between spouses: Evidence from longitudinal data in Singapore
Source: PLoS One. 2025 May 20;20(5):e0324113. doi: 10.1371/journal.pone.0324113 (PMC12091887; doi:10.1371/journal.pone.0324113)
Supplement: Attachment — Submitted filename: 0_Response to Reviewers - R&R_4th.docx [file pone.0324113.s026.docx]

**RESPONSE TO REVIEWERS**

PLOS ONE

Dear Editor,

Many thanks to you and the reviewers for helpful comments on the paper, Ref: **PONE-D-24-02155R3**, “Unequal Gains from Remote Work during COVID-19 between Spouses: Evidence from Longitudinal Data in Singapore.” In order to accommodate all comments and suggestions, we have undertaken major revisions to all sections of the paper.

**Reviewer #1**

*All comments have been addressed.*

**Response:**

Thank you very much for your guidance.

******************************************************************************

**Reviewer #2**

*Comments to the Author*

*The manuscript has improved from the previous version, and the authors have addressed the majority of my comments and suggestions. I thank the authors for their detailed responses. I have some additional comments, clarifications, and corrections of several inconsistencies that require further revision.*

**Response:**

Thank you very much for your guidance throughout the review process. We have made our best efforts to address your questions below.

*MAIN COMMENTS:*

**R2Q1:** *In the descriptive data in Table 2, the authors should comment that in pre-lockdown, lockdown and post-lockdown, WFH workers earn more than non-WFH workers (both in terms of monthly salary and hourly wage). This analysis is especially relevant because the estimates in Table 4 show the opposite result. According to the estimates in Table 4, before the lockdown period WFH workers earned less than non-WFH workers. Authors should provide an explanation for this contrary result between the descriptive analysis and their estimates.*

**Response:**

Thank you. Per your advice, we added a description regarding the difference between WFH and non-WFH workers in terms of monthly salary and hourly wage (p.22).

Regarding your second comment (“*According to the estimates in Table 4, before the lockdown period WFH workers earned less than non-WFH workers.*”), we believe you are referring to the negative coefficients of “Working Remotely’. However, because our DID estimation includes individual fixed-effects, and because our ‘Working Remotely’ variable varies across time, the coefficient of this variable measures the average within-person effects of adopting WFH across all time periods (not a cross-sectional difference). Thus, we cannot directly compare this coefficient with the difference in sample averages for WFH and non-WFH workers in the descriptive statistics (**Table 2**).

That being said, in the last paragraph of Section 5.2, we already addressed the issue you raise here: The regression-based pre-trends assessment in **Table 3** and **Table S7** showed that—based on the estimated coefficients of (Working Remotely x Apr-Jul 2018)—the WFH workers earned lower hourly wage and lower monthly income than non-WFH workers in the pre-lockdown period of April-July 2018. These indeed contrast the results from the summary statistics. The results in the descriptive statistics, shown in Table 2, are sample averages, while the results in Table 3/Table S7 are regression-based that includes individual fixed-effects. Based on this, we infer that the divergence between the summary statistics (Table 2) and the regression-based results (Tables 3/S7) stem from our inclusion/exclusion of the individual fixed-effects. We now include these explanations in the **Results Section** (Section 5.1, last paragraph, p.33).

**R2Q2:** *Due to the above comment, I am concerned about the econometric model specification (equation 1, page 27) that has significant consequences for interpreting the results. I am afraid there is a misunderstanding in interpreting the coefficients of the “lockdown” and “post-lockdown” variables. Apart from the “lockdown” dummy (equal to 1 for waves 3, 4, and 5) and the “post-lockdown” dummy (equal to 1 for wave 6), the estimates include time-fixed effects, occupation dummies, and the interactions between occupation and time-fixed effects. Regarding time-fixed effects, I guess the authors have included 5 dummies for the 6 waves, and the reference category is wave 1 (April-July 2018). If this is the case, the “lockdown” coefficient would measure the differential only against the first wave and not against the entire pre-lockdown period, as the authors point out. But additionally, since the “lockdown” dummy includes 3 waves (3, 4, and 5), the coefficient of “lockdown” measures the difference between wave 4 and wave 1 (because part of the effect of the lockdown is captured by the time-fixed effects that are not displayed in Table 4). Furthermore, the model includes interactions between occupation (4 categories?) and time-fixed effects (they do not indicate which category is the reference in estimates; I guess that is the first one, “Professionals”). These interaction terms make the interpretation of the coefficients even more complicated, since now, the reference category of the occupation also conditions the reference category for the period. The authors must clarify this point. If my interpretation were proper, all comments should be reviewed.*

**Response:**

You are absolutely right. As we are conducting panel analyses, and our outcome variables (salary income, hours worked, and hourly wage) do need to control for any within-occupation or within-wave specificities as well as occupation-specific changes across the waves, we believe the fixed-effects we have in our estimation are necessary. That being said, as you rightfully pointed out, our interpretation of the coefficients of the main regressors need to take into account the fact that our panel estimation include these fixed-effects.

For an individual i holding a job s in wave t, our estimation model is as follows:

$Y_{its}$=$\beta_{0}+\beta_{1}\left( {Remote}_{its} \right)+\beta_{2}\left( {Lockdown}_{t} \right)+ \beta_{3}\left( {Remote}_{its} \right)\left( {Lockdown}_{t} \right)+ \beta_{4}\left( {PostLockdown}_{t} \right)+ \beta_{5}\left( {Remote}_{its} \right)\left( {PostLockdown}_{t} \right)+\boldsymbol{X}_{it}\beta_{6}+$ $\lambda_{i}$ $+$ $\delta_{t}$ $+ \gamma_{s}+{(\delta}_{t}*\gamma_{s})+ \epsilon_{its}$ ***.***

Given our equation, we have now changed the interpretation of the coefficients in the following manner—to best describe the effects in the presence of the fixed-effects:

- $\boldsymbol{\beta}_{\boldsymbol{3}}\left( \boldsymbol{Remote}_{\boldsymbol{its}} \right)\left( \boldsymbol{Lockdown}_{\boldsymbol{t}} \right)$**:** This is the average treatment effect on the treated (ATT), or the treatment effect under lockdown for individuals who adopted remote work arrangements, relative to their pre-lockdown earnings, compared to their non-remote counterparts—*net of* fixed individual traits, wave-specific idiosyncrasies, occupation-specific trends, and occupation-specific time trends (hereafter called ‘occupation- and wave-specific confounding’)
- $\boldsymbol{\beta}_{\boldsymbol{5}}\left( \boldsymbol{Remote}_{\boldsymbol{its}} \right)\left( \boldsymbol{PostLockdown}_{\boldsymbol{t}} \right)$**:** This is the average treatment effect on the treated (ATT), or the treatment effect under post-lockdown for individuals who adopted remote work arrangements, relative to their pre-lockdown earnings, compared to their non-remote counterparts—net of occupation- and wave-specific confounding.
- $\boldsymbol{\beta}_{\boldsymbol{2}}\left( \boldsymbol{Lockdown}_{\boldsymbol{t}} \right)$: This captures changes in outcome during lockdown (March-June 2020) for non-remote workers, relative to pre-lockdown—reflecting the general pattern across waves within the ‘lockdown’ period, after controlling for wave-level idiosyncrasies and occupation-specific trends in each wave.
- $\boldsymbol{\beta}_{\boldsymbol{4}}\left( \boldsymbol{PostLockdown}_{\boldsymbol{t}} \right)$**:** This represents changes in outcome during post-lockdown (November 2020) for non-remote workers, relative to pre-lockdown—after controlling for wave-level idiosyncrasies and occupation-specific trends in each wave.
- $\boldsymbol{\beta}_{\boldsymbol{1}}\left( \boldsymbol{Remote}_{\boldsymbol{its}} \right)$**:** In the presence of individual fixed-effects, and because our ‘Working Remotely’ variable varies across time, the coefficient of this variable measures the average within-person effects of adopting WFH across all time periods (not a cross-sectional difference). In other words, this represents the effect of individuals’ adopting remote work across periods, compared to when they were not working remotely—net of wave-specific remote effects, time shocks, occupation effects, and occupation-specific time trends

Of note, we tried re-estimating the results without the occupation fixed-effects, wave fixed-effects, and occupation-specific wave trends. Results are shown in **S12 Table** in the **Appendix**. Once again, comparing the results to those shown in **Table 4**, we confirm that the estimated coefficients are robust to inclusion/exclusion of the occupation- and wave- fixed-effects as well as the occupation-specific time trends—in terms of direction and statistical significance.

| **S12 Table.** **Effects of Remote Work Arrangements on Hourly Wage, Monthly Income, and Monthly Work Hours** | | | | | | | | | |
| --- | --- | --- | --- | --- | --- | --- | --- | --- | --- |
|  | (1) | (2) | (3) | (4) | (5) | (6) | (7) | (8) | (9) |
|  | Y=Hourly Wage | | | Y=Monthly Income | | | Y=Monthly Hours Worked | | |
|  | All | Male | Female | All | Male | Female | All | Male | Female |
| (reference = Pre-Lockdown) | | |  |  |  |  |  |  |  |
| **Working Remotely x Lockdown** | 8.74*** | 6.88* | 9.97** | 308.28*** | 487.28*** | 92.39 | -10.02*** | -7.64 | -13.15*** |
|  | (3.13) | (3.67) | (4.82) | (115.39) | (185.12) | (125.69) | (3.71) | (4.95) | (4.88) |
| **Working Remotely x Post- lockdown** | 14.81*** | 19.40** | 13.25** | 294.41** | 536.94** | 87.64 | -35.19*** | -20.43* | -49.74*** |
|  | (4.80) | (8.69) | (5.37) | (149.60) | (220.02) | (179.49) | (9.46) | (10.97) | (14.97) |
|  |  |  |  |  |  |  |  |  |  |
| Working Remotely (1 yes 0 no) | -2.05 | -2.33 | -2.59 | -305.18*** | -507.96*** | -62.20 | 6.49 | -4.17 | 18.99** |
|  | (3.49) | (5.24) | (4.26) | (113.57) | (193.19) | (122.96) | (5.44) | (7.10) | (9.10) |
|  |  |  |  |  |  |  |  |  |  |
| Lockdown | 8.85*** | 10.24*** | 7.24*** | -160.01** | -247.58** | -7.34 | -5.40 | -11.39** | 0.92 |
|  | (2.50) | (3.82) | (2.17) | (76.40) | (109.36) | (80.85) | (3.74) | (4.56) | (4.99) |
| Post-lockdown | 7.24*** | 8.56** | 4.63* | 91.71 | 82.23 | 151.23 | 4.34 | -2.80 | 13.00* |
|  | (2.71) | (3.94) | (2.80) | (85.56) | (122.13) | (95.95) | (5.46) | (6.12) | (7.32) |
|  |  |  |  |  |  |  |  |  |  |
| Individual FE | Yes | Yes | Yes | Yes | Yes | Yes | Yes | Yes | Yes |
| Occupation FE, Time FE, Occupation-specific Time Trends | No | No | No | No | No | No | No | No | No |
| Control variables | Yes | Yes | Yes | Yes | Yes | Yes | Yes | Yes | Yes |
| Mean Dependent Variable | 35.43 | 37.37 | 33.24 | 4900.88 | 5488.48 | 4227.20 | 172.83 | 180.36 | 164.19 |
| N | 4308 | 2301 | 2007 | 4308 | 2301 | 2007 | 4308 | 2301 | 2007 |
| Notes: Remote is a dichotomous variable for whether the respondent worked fully from home in May 2020 during the COVID-19 lockdown. The reference time period is ‘Pre-lockdown’, between April-July 2018 and December 2019, prior to the COVID-19 pandemic. ‘Lockdown’ refers to March and June 2020, while ‘Post-lockdown’ refers to November 2020, six months after the end of the lockdown. † denotes that the male-female differences in the estimated coefficients are statistically significant (p<0.05). Standard errors, shown in the parentheses, are clustered at the household level. | | | | | | | | | |
| *p<0.1 **p<0.05 ***p<0.01 | | | | | | | | | |

**R2Q3:** *As I indicated in my previous report, it is also important that authors carefully review the interpretation of the interaction terms as there continue to be inconsistencies in their comments. I point out a single example, but it is necessary to review all of them. Page 46: “In the post-lockdown period, female WFH workers’ monthly hours dropped by as much as 49.34 hours/month”. According to estimates showed in Table 4, in the post-lockdown period, female WFH workers’ monthly hours dropped by as much as 36.49 hours/month, and an increase in hours work of 12.85 for female non-WFH. Hence, in the post-lockdown period the difference between WFH workers y non-WFH workers is -49.34 hours/month. Logically, this interpretation would also need to change if my second comment were right.*

**Response:**

Per your advice in this question and the earlier ones, we now have carefully revised all our interpretations in the Results Section and in the Discussion Section.

Of note, we believe there is a typo in your estimation. The total effect on female WFH workers’ monthly hours in post-lockdown (using Table 4, column 9) would be -49.34+ 17.44 = -31.90 hours/month, not -49.34+12.85= -36.49 hours/month. In other words, the total effect would need to sum the coefficient of the interaction term (effect of WFH in lockdown/post-lockdown) and of the ‘remote’ indicator (effect of WFH in pre-lockdown). In the meantime, -36.49 hours/month would refer to the effect of WFH in post-lockdown, relative to being non-WFH in pre-lockdown—conflating time and treatment.

**R2Q4:** *Page 43: The authors compare their estimated coefficients with the official mean wages for men and women in Singapore (SGD $4,719 and SGD $4,374, respectively). This is not correct, their coefficients are only comparable with the average values of their data, which, as the authors themselves point out, are not comparable with the total universe because their data is a subsample of married men and women.*

**Response:**

We fully agree with your comment. Thus, we have now removed the population average monthly income values from the sentence. Instead, we calculated the sample average of the outcome variables (specified in red in Table 4, pasted below), and discussed the percent increase from the sample averages. Now, the sentence reads: “*Considering that the average monthly income* ***in our sample was SGD $5,488.48 for males and SGD $4,277.20 for females****, the increased monthly income generated by adopting WFH arrangements under lockdown/post-lockdown constitutes roughly 7.1-8.1% growth for men (i.e., an increase by SGD $387.43 under lockdown and SGD $446.42 in post-lockdown from the* ***mean of SGD $5,488.48****, from Table 4, column 5) while comprising mere 1.2-1.6% (i.e., an increase by SGD $65.97 under lockdown SGD $52.52 in post-lockdown from the* ***mean of SGD $4277.20****, from Table 4, column 6) growths for women.*” (p.46)

| **Table 4.** **Effects of Remote Work Arrangements on Hourly Wage, Monthly Income, and Monthly Work Hours** | | | | | | | | | | |
| --- | --- | --- | --- | --- | --- | --- | --- | --- | --- | --- |
|  | (1) | (2) | (3) | (4) | (5) | (6) | (7) | (8) | (9) |  |
|  | Y=Hourly Wage | | | Y=Monthly Income | | | Y=Monthly Hours Worked | | | |
|  | All | Male | Female | All | Male | Female | All | Male | Female |  |
| (reference = Pre-Lockdown) | | |  |  |  |  |  |  |  |  |
| **Working Remotely x Lockdown** | 8.43*** | 7.24* | 8.73* | 272.34** | 387.43** | 65.97 † | -9.54** | -6.99 | -12.98** |  |
|  | (3.14) | (3.71) | (5.18) | (106.74) | (178.28) | (120.13) | (3.72) | (5.11) | (5.21) |  |
| **Working Remotely x Post- lockdown** | 14.78*** | 20.04** | 12.20** | 267.06* | 446.42** | 52.52 † | -34.87*** | -20.61* | -49.34*** |  |
|  | (4.83) | (8.81) | (5.78) | (148.84) | (226.18) | (185.72) | (9.49) | (11.12) | (15.15) |  |
|  |  |  |  |  |  |  |  |  |  |  |
| Working Remotely (1 yes 0 no) | -1.43 | -2.35 | -0.86 | -265.18** | -393.37** | -53.75 † | 5.67 | -5.05 | 17.44* † |  |
|  | (3.55) | (5.48) | (4.43) | (103.81) | (186.13) | (119.01) | (5.38) | (7.30) | (9.11) |  |
|  |  |  |  |  |  |  |  |  |  |  |
| Lockdown | 9.37*** | 10.91*** | 8.76*** | -116.93 | -182.50* | -17.30 † | -5.27 | -12.53** | 1.05 † |  |
|  | (2.53) | (3.89) | (2.43) | (75.96) | (106.38) | (91.15) | (4.01) | (5.04) | (5.49) |  |
| Post-lockdown | 7.89*** | 9.35** | 6.42** | 135.76 | 150.04 | 132.54 | 4.36 | -4.11 | 12.85* † |  |
|  | (2.81) | (4.06) | (3.11) | (90.31) | (128.67) | (105.55) | (5.66) | (6.51) | (7.63) |  |
|  |  |  |  |  |  |  |  |  |  |  |
| Individual FE, Occupation FE, Time FE | Yes | Yes | Yes | Yes | Yes | Yes | Yes | Yes | Yes |  |
| Occupation FE x Time FE | Yes | Yes | Yes | Yes | Yes | Yes | Yes | Yes | Yes |  |
| Control variables | Yes | Yes | Yes | Yes | Yes | Yes | Yes | Yes | Yes |  |
| Mean Dependent Variable | 35.43 | 37.37 | 33.24 | 4900.88 | 5488.48 | 4227.20 | 172.83 | 180.36 | 164.19 |  |
| N | 4308 | 2301 | 2007 | 4308 | 2301 | 2007 | 4308 | 2301 | 2007 |  |
| Notes: Remote is a dichotomous variable for whether the respondent worked fully from home in May 2020 during the COVID-19 lockdown. The reference time period is ‘Pre-lockdown’, between April-July 2018 and December 2019, prior to the COVID-19 pandemic. ‘Lockdown’ refers to March and June 2020, while ‘Post-lockdown’ refers to November 2020, six months after the end of the lockdown. † denotes that the male-female differences in the estimated coefficients are statistically significant (p<0.05). Standard errors, shown in the parentheses, are clustered at the household level. | | | | | | | | | | |
| *p<0.1 **p<0.05 ***p<0.01 | | | | | | | | | | |

*MINOR COMMENTS:*

**R2Q5:** *Page 3. Indicate the source for the reported data for Netherlands (47.4%), Denmark (53.0%), and U.S. (50%).*

**Response:**

Thank you for spotting the missing source. Upon investigation, we realized that the share for European countries and the US were referred to individual working remotely among the ones who wished to do so. We now have cited more accurate sources showing the correct percentages of remote workers out of the total employed: 35.2% in the US (Bick et al. 2020), and 25% Finland, 23.1% Luxembourg (Eurostat, 2020).

**R2Q6:** *Page 4. Substitute “mediating roles of housework” by “mediating roles of household responsibilities” in the sentence “Lastly, we explore potential explanations for these gender differences by exploring the mediating roles of housework.”*

**Response:**

We have made the adjustment.

**R2Q7:** *Page 7. Substitute “the rate of deceleration of GPD slowed down” by “the rate of decline of GPD slowed down”.*

**Response:**

We have made the adjustment.

**R2Q8:** *Figure 1. Labels in horizontal axis in panel A and panel B should be homogenised. In addition, the numbers in vertical axis appear with % in panel A but not in panel B.*

**Response:**

We have homogenized the horizontal axis in panel A and B to better fit the analytic period of our study. The vertical axis in both panels now show %.

**R2Q9:** *Page 15, first paragraph. Delete “hourly wages” in “(monthly salary income, monthly work hours, hourly wages)” (the questionary does not ask about hourly wages).*

**Response:**

We have changed the sentence.

**R2Q10:** *Page 16. The authors claim that they interviewed 660 persons, and then they dropped 5 persons that were not married. Then, they drop those not employed persons across the six waves. If the number of women in the final sample is 335, this implies that they dropped 320 women (49% of 655). The final sample for men is 384, meaning they dropped 271 men (41% of 655). Nevertheless, in the Conclusions section, the authors claim that “the number of respondents we dropped from our sample due to unemployment in 2020 comprised only 8.3% (360 person-wave observations across six survey waves, or 60 unique individuals)of the total sample of 4,308 person-wave observations.”*

**Response:**

The statements still stand. In the first portion of your comment, you are talking in terms of unique individuals in our sample. However, given that we have to drop some individuals in only some of the survey waves (keeping them in other waves), we have to count in terms of person-wave observations.

Our sample is an unbalanced panel data, where we have multiple records of the same individuals across 6 panel waves. We discuss the number of drops in terms of person-wave observations in our paper:

“*From the 7,920 person-wave observations, we dropped 68 observations who were separated or divorced, leaving 655 men and 655 women in waves 1-5, and 651 each in wave 6. The continuous marital status was important in that, if a respondent was divorced in a survey wave, this person would have no response to provide for the husband. Because we explored the role of household work division across spouses as mediators in this study, we required the spouse information. Next, we dropped 3,184 observations (all observations for 531 individuals across waves) for not working in waves 1-2 in 2018-2019, prior to COVID-19 and Lockdown, as changes across time in their monthly income or hourly wages could be measured.* ***We further dropped from our final sample 360 person-wave observations for individuals who worked in waves 1-2 but stopped working in waves 3-6 in 2020 and individuals who stayed unemployed/out of the labor force for all 6 waves.*** *This was to distinguish the effect of remote work adoption from that of job displacement/acquisition across waves. Including individuals who did not work in 2018-2019 and started working in 2020 would have introduced an upward bias to the effect of remote work arrangements. If such individuals engaged in WFH jobs in 2020, their income and wages would have experienced a steep jump from zero. This would not necessarily be a labour market outcome associated with the adoption of WFH arrangements. Rather, this would be the outcome associated with transitioning from the non-working state to the working state. Similarly, including in the sample individuals who worked in waves 1-2 but stopped working in 2020 would have generated a downward bias. Lastly, individuals without jobs in 2020 would not be able to be categorized based on the extent of WFH they have adopted (i.e., our main regressor). In short, we intentionally kept only the employed individuals to measure the effect of adopting remote work arrangements conditional on individuals’ continued employment throughout the analytic period. Our sample accommodated switching between jobs.*” (p.17).

In the bold statement, we describe that we dropped 360 person-wave observations (approximately 60 unique individuals given that there are 6 waves, but this is not an exact number since we have an unbalanced panel data). Magnitude-wise, this portion comprises about 8.3% of the final sample, which has 4,308 person-wave observations. These numbers match what we describe in the Conclusion section.

**R2Q11:** *Page 27, equation 1. The variables Lockdown and Postlockdown should not include temporal subscripts.*

**Response:**

We believe the Lockdown and the Post-Lockdown variables *do* need the temporal subscripts. Because these are time indicators—specifying particular moments in our analytic period—these indicators change value across time (from 1 to 0, and vice versa) for person-wave observations. We changed the description of these time indicators as follows, to be clearer:

*“*${Lockdown}_{t}$ *was a binary temporal indicator,* ***taking on the value 1 if in March-June 2020 (pooled), 0 if otherwise****, and* ${PostLockdown}_{t}$ ***took on the value 1 if in November 2020, 0 if otherwise.****” (p.28)*

**R2Q12:** *Page 25. Typo. “another: A plurality” should be “another. A plurality”.*

**Response:**

We have made the adjustment.

**R2Q13:** *Page 52. Typo: Include a space in “60 unique individuals)of”*

**Response:**

We now have added a space.

**REFERENCES**

Bick, A., Blandin, A., & Mertens, K. (2020). Work from Home after the COVID-19 Outbreak. Federal Reserve Bank of Dallas, working paper 2017. <https://doi.org/10.24149/wp2017r1>

Eurostat (2020). Employed Persons Working from Home as a Percentage of the Total Employment, by Sex, Age, and Professional Status. Employment-LFS Series. Accessible at: <https://doi.org/10.2908/LFSA_EHOMP>
